# Machine learning-assisted analysis of serum metabolomics and network pharmacology reveals the effective compound from herbal formula against alcoholic liver injury

**DOI:** 10.1186/s13020-025-01094-1

**Published:** 2025-04-11

**Authors:** Jiamu Ma, Peng Wei, Xiao Xu, Ruijuan Dong, Xixi Deng, Feng Zhang, Mengyu Sun, Mingxia Li, Wei Liu, Jianling Yao, Yu Cao, Letian Ying, Yuqing Yang, Yongqi Yang, Xiaopeng Wu, Gaimei She

**Affiliations:** 1 Beijing University of Chinese Medicine, Fangshan District, Beijing, 100029 China; 2 Analysis and Test Center, Chinese Academy of Tropical Agricultural Sciences, Haikou, 571101 China

**Keywords:** Effective compounds, Traditional Chinese medicine, Network pharmacology, Serum metabolomics, Machine learning, Compound-target interaction

## Abstract

**Background:**

The popularity of herbal formulas is increasing worldwide. Nevertheless, the effective compound is challenging to identify due to its intricate composition and multiple targets.

**Methods:**

An integration machine learning-assisted approach was established, whereby the particular action mechanism and direct target were obtained through the correlation of compounds, targets, and metabolites. The association between a compound and an action pathway was selected from the shortest path of the “compound-target-pathway-disease” network, which was analyzed using the Floyd-Warshall algorithm. Subsequently, an investigation was conducted into the relationship between metabolites and action pathways, as well as targets, through the analysis of serum metabolomic profiling and the selection of metabolite biomarkers by random forest. In order to accurately identify the direct acting target as well as the most effective compound, the relationship between the compounds and their targets was investigated using a feature-based prediction model conducted by AdaBoost. The binding mode of the effective compound and the direct-acting target was verified by molecular docking, dynamics simulations, and western blotting. In this study, Baiji Wuweizi Granule (BWG) was employed to elucidate the effective compound against alcoholic liver injury (ALD).

**Results:**

BWG exerted an influence on the serum metabolomic, resulting in the identification of seven potential biomarkers. Furthermore, six effective compounds and the PI3K-AKT signalling pathway were identified through a co-analysis with the shortest path from compound to ALD in the “compound-target-pathway-disease” network. It was postulated that the effective compounds would bind with key targets from the PI3K-AKT signaling pathway, as indicated by the prediction model of compound-target interaction (R^2^ > 0.95). The dominant bonding type for the effective compounds and key targets was hydrogen bond. These results indicated that AKT1 was the notable target for BWG, and that 2,3,4,7-tetramethoxyphenanthrene was the marker compound for BWG against ALD. The present study provides evidence that the protective effect of BWG on ALD can be mediated by the PI3K-AKT signaling pathway.

**Conclusions:**

Our findings demonstrate the value of a machine learning-assisted approach in identifying the key compound, target and pathway that underpin the efficacy of an herbal formula. This provides a foundation for future clinical and fundamental research.

**Supplementary Information:**

The online version contains supplementary material available at 10.1186/s13020-025-01094-1.

## Introduction

Alcohol-related damage is a critical public health issue caused by excessive alcohol consumption, with the most common causes being gastrointestinal disorders such as alcoholic liver disease (ALD) and gastric injury [1, 2]. Numerous epidemiological researches have demonstrated the close correlation between alcohol consumption and ALD, while the relationship between alcohol consumption and stomach damage risk is not statistically significant [2, 3]. ALD is the first line to defense against alcohol-related damage to the body. As a result, early intervention in the course of ALD is important for the prevention and treatment of alcohol-related gastrointestinal disorders. Despite of the alcohol abstinence and nutritional support, there is still a shortage of effective therapies and medications. According to the clinical recommendation, hepatoprotective medications employed for the treatment of alcoholic liver injury, like silymarin and polyene phosphatidylcholine [4–6]. Though they have been shown to enhance liver biochemical indicators through a range of mechanisms, lacking of compelling experiment evidence, wide range of action pathway, and also the potential of drug interaction restrict the in-depth application of these medicals.

Homology of medicine and food from traditional Chinese medicine (TCM) is a promising source for discovering effective materials on ALD nowadays with higher safety and proven effectiveness [7, 8]. Because of the complexity of compounds and the characteristics of multi-target actions, it is a great challenge to accurately identify the effective compound of TCM herbal formula [9, 10]. Baiji Wuweizi Granules (BWG), which is composed with *Astragali Radix* (AR), *Bletillae Rhizoma* (BS), *Schisandrae Chinensis Fructus* (SC), and *Citri Reticulatae Pericarpium* (CP), is taken as a case for insight. According to our previous study, BWG has protective effect on liver disease and gastric disease [11, 12]. Whereas, the effective compounds to ALD have not elucidated due to the complex compounds consisting. Unknown effective compounds and the acting mechanism limit the application of BWG.

Recently, metabolomics analysis has become a reliable method to explain the protective effect of early drug discovery. Metabolic profiling comprehensively maps all biochemical reactions in the system of drug intervention. Untargeted method, like serum metabolomics, aims to select promising targets for disease, followed by the screening of possible interactions [13]. Nevertheless, it is a great challenge to characterize the previous targets and distinct metabolites as biomarker which limit the exploration of underlying mechanisms. Combination of network-based analysis of compound, target, metabolite, and pathways provides another insight to face up to this challenge. Analysis of network provides a more microscopic future to herbal formula [14, 15]. Topological analysis of “compound-target-pathway” network is recognized to be a promising approach for screening potential valuable compound and target. Unlike the conventional topological analysis, for example, degree, closeness, mainly focus on the importance of single node, shortest path of node pair is another significant network topological parameter to explain the biological network structure and dynamics [16]. Study on shortest path of compound-disease is able to find the closest interaction between them, meanwhile reporting nodes and edges along also provide a fresh view of explaining biological interaction [17, 18]. To make up for the regrets of several core targets of screened pathways may be missed during analytical process, describing the relationship between compounds and targets become an important step.

Computational methods provide a new hope to address these necessities. Facing the growing demands of identifying correlations among big data of biomedical system, computational methods, including machine learning, molecular docking, and molecular dynamics, has been a popular and efficient method to infer the dynamics of complex biology behaviors [19–22]. The identification of effective compound from herbal formula was divided into three parts, including relationships among compound, target and pathway, metabolite, pathway and target, as well as the relations between pathway and disease, which analysis were all assisted by computational methods. Thus, machine learning is applied in selecting key feature of biomedical system, which consist of metabolite, compound, target, as well as pathway. Molecular docking is able to provide a static view of compound-target interaction, meanwhile the interactions in motion, fluctuation in residues, and movement in any specific domain or separation of compound-target complex can be learned by molecular dynamics simulation.

In this study, the integrated approach was validated based on the protective effect of BWG on ALD as well as the quantification of main compounds of BWG firstly. Then analysis of serum metabolomics is adapted for identifying the differential metabolites which provides the basic information of metabolism pathway and also the metabolite biomarker. A “compound-target-pathway-disease” network (CTPDN) is conducted on network pharmacology as following, which offers a visual way for screening the effective compounds and distinct acting targets. To figure out the relationship of effective compounds and key targets of pathways that are not selected in CTPDN, a prediction model of compound-target interaction has been generated. Finally, the interaction between effective compounds and key targets are validated by molecular docking, molecular dynamics and western blotting as well. This study provides the evidence of BWG’s protective effect on ALD as well as the identification of its effective compounds. Taken together, the established network-based approach provides an accurate and efficient method of identifying the effective compound, its specific target, and the action pathway, laying the foundation for elucidating the composition basis and mechanism of herbal formula.

## Materials and methods

### Materials and reagents

BS, AR, SC and CP were purchased from Yuzhou Kaixuan Pharmaceutical Co., Ltd. Folin-Ciocalteo reagent (M25IS16394A), vanillin (F08IS206772), and hesperidin (M05M8S35241) were purchased from Shanghai yuanye Bio-Technology Co., Ltd (Shanghai, China). Militarine (112061–202001) and schisandrin (110857–201815) were purchased from National Institutes for Food and Drug Control (China). Formononetin (AF20052952) and nobiletin (AF20032911) were purchased from Chengdu Efa Biotechnology Co., Ltd (Sichuan, China).

C57BL/6 J mice were purchased from Beijing Vital River Laboratory Animal Technology Co., Ltd. (Beijing, China). Silymarin was purchased from MEDA Pharmaceuticals GmbH. (Germany). Lieber-DeCarli diet was purchased from Trophc Animal Feed High-Tech Co., Ltd. (Jiangsu, China). Antibodies for AKT1, STAT1, CAT, were obtained from Proteintech Inc. (Hubei, China). Antibodies for pAKT1, β-actin, goat anti-mouse IgG (H + L), goat anti-rabbit IgG (H + L) secondary antibodies were obtained from ImmunoWay Biotech. Co. (USA). Glutamic pyruvic transaminase (ALT) kit and glutamic pyruvic transaminase (AST) kit, BCA kit, malondialdehyde (MDA) kit, superoxide dismutase (SOD) kit, triglyceride (TG) kit, total cholesterol (TC) kit were purchased from Nanjing Jiancheng Bioengineering Institute. All chemicals were of analytical grade.

### Preparation of Baiji Wuweizi Granule

The extraction of BWG was same as the team’s published work [11]. In brief, 2.0 g SC of extracted by reflux with 24.0 mL of 60% ethanol for 1 h, and this process shall be carried out a total of three times. The optimized water extraction process could be described as follows: 6.0 g AR, 3.0 g BS, 2.0 g SC and 3.0 g CP were weighed, 168 mL water was put into the box and lasting for 1.5 h boiling, and the process was carried out a total of two times.

### Molecular network analysis via UHPLC-MS/MS

The detection of BWG compounds by UHPLC-MS/MS was referenced as our published work [11]. The molecular network of BWG was created on the GNPS website (http://gnps.ucsd.edu). The precursor ion mass tolerance was set to 2.0 Da and a MS/MS fragment ion tolerance of 0.5 Da. A network was then created where edges were filtered to have a cosine score above 0.7 and more than 6 matched peaks. Further, edges between two nodes were kept in the network if and only each of the nodes appeared in each other’s respective top 10 most similar nodes. Finally, the maximum size of a molecular family was set to 100, and the lowest scoring edges were removed from molecular families until the molecular family size was below this threshold. All matches kept between network spectra and library spectra were required to have a score above 0.7 and at least 6 matched peaks.

### Content analysis of main components

#### Total phenolics content

Total phenolics content was determined by Folin-Ciocalteo method with a little modification [23]. Briefly, 0.5 mL BWG sample solution (1.0 mg/mL) was added to 2.0 mL of 0.1 mol/L Folin-Ciocalteo reagent. Then 2.0 mL of 7.5% (w/v) Na_2_CO_3_ solution after reacting for 3 min. The mixed solution was put in dark for 60 min and the absorbance was detected at 750 nm. Results were expressed as gallic acid equivalent (GAE) (mg GAE/L).

#### Total flavonoids content

Total flavonoids content was determined by AlCl_3_ colorimetric method [23]. Results were expressed as rutin equivalent (RE) (mg RE/mL).

#### Total sugar content

Total sugar content was determined by phenol–sulfuric acid method with slightly modified. The optimization process of this method was shown in Supplementary material 1.1. In brief, 0.25 mL BWG sample solution (10.0 mg/mL) was mixed with ethanol until the concentration reached 80% (v/v). After stored overnight, the mixture was centrifuged at 7000 r/min for 15 min and the supernatant was discarded. Then the precipitate was dissolved in 5 mL water and the test solution was made. 2.0 mL test solution was mixed with 1.2 mL of 5% phenol solution, and 5.5 mL of sulfuric acid was slowly added in this mixture. After reacting at 90 ℃ water bath for 15 min, the mixed solution was cooled and measured at 485 nm. Results were expressed as glucose equivalent (Glc) (mg Glc/L).

#### Total triterpenoids content

Total triterpenoids content was determined by vanillin-perchloric acid method [24]. Results were expressed as oleanolic acid equivalent (OA) (mg OA/L).

#### Quantification of five compounds by HPLC–DAD

The content of quality evaluation compounds of BWG (hesperidin, militarine, formononetin, nobiletin, and schisandrin) were determined by the HPLC–DAD method. The equipment and chromatographic conditions were described in detail in Supplementary material 1.2.

### Animal experiments

#### Animals and model treatment

Male C57BL/6 J mice (7–8 weeks old, 18–22 g) were purchased from Charles River Laboratory (Beijing, China). The mice were housed in a temperature and humidity controlled room (23 ± 2 and 55 ± 5 relative humidity) with a 12 h light/dark cycle. All experimental protocols were approved by the committee on Ethics of Animal Experiments (BUCM-4–2022060501–2122) at Beijing University of Chinese Medicine, China. All animal experiments were carried out in accordance with the ethical standards laid down in the 1964 Declaration of Helsinki and its later amendments.

Mice were fed with a Lieber-DeCarli diet containing up to 4% alcohol for 42 days (EtOH-fed) or Lieber-DeCarli diet alone (pair-fed). All mice were randomly divided into six groups after adaptive feeding for one week. Control (Con) and Model (Mod) group were received normal saline (0.2 mL/d), gastric irrigation. Positive group (Pos) were received silymarin of 100 mg/kg. Low dosage BWG (BWG-L), medium dosage BWG (BWG-M) and high dosage BWG (BWG-H) were received BWG of 175 mg/kg、350 mg/kg, and 700 mg/kg, which calculated by 2.5 times, 5 times and 10 times of the daily usage of people, respectively [11]. The doses of the silymarin and BWG were converted into corresponding doses based on the body surface area of humans and animals.

#### Histology analysis

The histology was performed as the previous study [25]. In brief, half of the largest lobe of liver tissue was fixed in 4% paraformaldehyde solution for more than 24 h. Then the tissue was stained with hematoxylin and eosin. The liver stertosis status morphology was examined under a light microscope (Nikon, Japan).

#### Biochemical index determination

Serum ALT and AST, liver tissue MDA, SOD, TG and TC levels were determined by using commercial kits from Nanjing Jiancheng Bioengineering Institute (Nanjing, China) according to the manufacturer’s instructions.

### Serum metabolomics analysis

#### Sample preparation

100 μL serum samples of Con, Mod, and BWG-H (which was called as BWG in metabolomics analysis) groups were precipitated by adding 300 μL cold methanol. After 1 min vortexing, the mixed sample was centrifugation at 12,000 ×*g* for 15 min at 4 ℃, and the supernatant was prepared for the UPLC-MS/MS analysis. The quality control samples (QC) were prepared by mixing equal volumes of samples from Con, Mod and BWG-H group.

#### Metabolomics analysis

For the serum metabolomics analysis, samples were analyzed by using Ultimate 3000 UPLC system (Dionex, USA) coupled online to a Q Exactive Plus MS instrument (Thermo Fisher Scientific, MA, USA). Chromatographic separations were performed on a Waters HSS T3 UPLC column (2.1 × 100 mm, 1.8 μm). The detailed setting chromagraphic and mass spectromatic programs were shown in Supplementary material 1.3.

#### Data analyses

The raw data were processed by Xcalibur 4.1 (Therma, MA, USA) and MS-DIAL 5.1.2 (http://prime.psc.riken.jp/compms). A list of data information consisted of mass-to-plasma charge ratio, retention time and peak area were collected. The list features with more than 80% missing values were removed, while the remaining missing values were replaced by the mean of that feature. Features were then filtered by the RSDs greater than 20% compared to the QC sample and the 40% features were variance filtered by standard deviation (SD). The peak area of the features was normalized by the sum, while the peak area was log (base 10) transformed and mean centred scaled. PLS-DA, t-test, and orthogonal partial least squares discriminant analysis (OPLS-DA) were performed to determine the differences in the raw data comparing the Con and Mod groups, as well as the Mod and BWG groups. Differential metabolites were screened under the conditions of variable importance projection (VIP) > 1, *p* < 0.05, and the absolute value of fold change (FC) > 1. Differential metabolites were identified by PubChem (https://pubchem.ncbi.nlm.nih.gov/), HMDB (https://hmdb.ca/) and MassBank (https://massbank.eu/MassBank/). Metabolic pathways were constructed by KEGG, which had *p* < 0.05 were deemed significant metabolic pathways potentially influenced by BWG. Biomarker selection was performed by classical univariate receiver operating characteristic (ROC) curve and random forests ROC curve analyses. The analyses were performed by MetaboAnalyst 5.0 (https://www.metaboanalyst.ca/).

### Machine learning-assisted network pharmacology analyzing

#### Collection of nodes from CTPDN

Nodes from CTPDN were divided into four parts that each elements of compounds from BWG, targets, signaling pathways, and disease were incorporate into the construction of the network. The collection of these nodes as well as their criteria were summarized as follows.

Nodes of compounds were collected from the Traditional Chinese Medicine Systems Pharmacology Database and Analysis Platform (TCMSP) and Traditional Chinese Medicine Integrated Database (TCMID) [26, 27]. Common amino acids, monosaccharide and polysaccharide were excluded in this study. Some other important compounds were collected from publications as a complement. Oral bio-availability (OB) ≥ 30% and drug-likeness value (DL) ≥ 0.18 were used as prerequisite parameters to select potential functional compounds of all.

Nodes of targets were the intersection of disease targets and compound-related targets. The potential gene targets of diseases were screened with the key words of disease name on Genecards and OMIM [28, 29]. Compounds got their relative gene targets with the help of Drugbank and TCMSP [30]. Correction and unification of target names were carried out by the Uniprot database. Then the same targets oriented from diseases and drugs were taken into intersection by Venn diagram analysis. The same targets were imported into STRING 11.2 for protein–protein interaction (PPI) network, while the species was limited to “Homo sapiens” and other setting was as default [31]. Cytpscape 3.9.1 and Molecular Complex Detection (MCODE) app were employed to visualize the PPI network and hub gene group [32], which the targets from hub gene group were eventually selected into the CTPDN network.

Nodes of signaling pathways were collected by the analysis results of GO and KEGG enrichment, which provided a way for systematically discern the multiple therapeutic mechanisms of TCM formula with comorbid disease [33]. “Bioconductor” in R language was used to analysis GO and KEGG pathways for further study. All settings were set as default. Raw data of pathways could reposition to the results of KEGG enrichment. Firstly, focusing on the research topic, the significant pathways (*p* < 1 × 10^–8^) were selected as further analysis, then those pathways without clustering gene targets were deleted, and the pathways had no relation with liver disease or gastric mocusa disease based on the publications were also excluded from this study. Considering about lowing the fake positive possibility, *q* value was adopted to define the importance of pathways [34]. A list of pathway name, *q* value, concluding targets was organized for further analysis. As for the nodes of disease consisted of liver injury disease and gastric mocusa disease.

#### Obtaining weighted edges of CTPDN

Edges of CTPDN included three types which were compound-target interaction, target-pathway interaction and pathway-disease interaction. The obtaining of these types of interaction as well as their weight were shown as following.

Compound-target interaction were defined by the combing capacity of molecular docking which Discovery Studio 4.0 (DS 4.0) was employed. The 3D structures of compounds were obtained from PubChem. Crystallographic structures of target proteins were downloaded from RCSB Protein Data Bank. With deleting the water and macromolecule, pose cluster radius of top hits were set as 0.5 before docking. Other settings were all set as default. In case the compound may have rigid structure with high personal energy that would lead to difficult docking, “-CDOCKER INTERACTION ENERGY” was considered to be a better choice as decision score. Due to some compounds may rigid resulting in the docking failure, score of compound-target interaction was weighted by:

1 $$S=\frac{{s}_{i}}{{s}_{m}}$$

which *s*_*i*_ is the CD interaction value of binding between each compound and target, *s*_*m*_ is the CD interaction value of binding between original macromolecule and target. Score reflects strength of capacity to candidate compound compare to the original macromolecule.

The target-pathway interaction were obtained by the extraction cluster of PPI network. Due to the complex relations among proteins, the weight score of relatives between targets were referenced the reciprocal of combine score from PPI network. The pathway-disease interaction was defined according to the publications while the weight was defined by the *q* value.

#### Construction of “compound–target–pathway-disease” network

Since compound taking effect mainly depended on combination with protein targets and then acting on relative pathways, we modeled CTPDN, which was proposed to have the direction from compound as beginning node, going along with target and pathway, eventually ended in disease. Floyd-Warshall algorithm is a method to find the shortest path between two random nodes with weighted edges. The algorithm was run to obtained the shortest path from disease to compound in this network. On the other words, this algorithm was used and modified to figure out the value of candidate compounds to the disease. Therefore, the picked compound was believed be the top rank in the formula based on the dynamic planning theory [35].

Compounds from TCM formula, hub targets group, core relative pathways and diseases were set as nodes and paired with their corresponding relations. With their names importing, the significant biological relatives between nodes, including compound-target interaction (CTI), target-target interaction (TTI), target-pathway interaction (TPI) and pathway-disease interaction (PDI), were set as edge containing weight importing either.

All weighted scores for calculating shortest path length among CTPDN were standardized prior to the Floyd-Warshall algorithm running. Base standardization equations for equalize the weight data of different types, the length of sub path on the other words, was as follows:

2 $${L}_{j}=\left\{\begin{array}{c}\frac{1}{{S}_{j}}, if two nodes has interaction\\ +\infty , if two nodes has no interaction\end{array}\right.$$

where *S*_*j*_ is the calculated score from edges of CTI, TTI and TPI, *j* denotes to the type of various interactions. To bring up with, the score of PDI was defined as 1.

Then to overcome the barriers evaluating different types of data, every type of edges is normalized as follows:

3 $${W}_{j}=\left\{\begin{array}{c}1, pathway- disease interaction\\ \frac{1}{1+{e}^{-{L}_{j}}}, otherwise\end{array}\right.$$

where *L*_*j*_ denotes the length between nodes as well as the importance of interaction between them.

Next, we represented the edges of CTPDN with an adjacency matrix with the form:

4 $$A=\left[\begin{array}{cc}\begin{array}{cc}\begin{array}{c}\begin{array}{cc}\begin{array}{c}0\\ \vdots \end{array}& \begin{array}{c}\cdots \\ 0\end{array}\end{array}\\ \begin{array}{cc}\begin{array}{c}{W}_{j}\\ \vdots \end{array}& \begin{array}{c}\ddots \\ \ddots \end{array}\end{array}\end{array}& \begin{array}{c}\begin{array}{cc}\begin{array}{c}{ W}_{j}\\ \vdots \end{array}& \begin{array}{c}\cdots \\ \ddots \end{array}\end{array}\\ \begin{array}{cc}\begin{array}{c}0\\ \ddots \end{array}& \begin{array}{c}\ddots \\ 0\end{array}\end{array}\end{array}\end{array}& \begin{array}{cc}\begin{array}{c}\begin{array}{cc}\begin{array}{c}inf\\ \ddots \end{array}& \begin{array}{c}\cdots \\ \ddots \end{array}\end{array}\\ \begin{array}{cc}\begin{array}{c}\ddots \\ \ddots \end{array}& \begin{array}{c}\ddots \\ \ddots \end{array}\end{array}\end{array}& \begin{array}{c}\begin{array}{cc}\begin{array}{c}{W}_{j+1}\\ \ddots \end{array}& \begin{array}{c}\cdots \\ \vdots \end{array}\end{array}\\ \begin{array}{cc}\begin{array}{c}\ddots \\ \ddots \end{array}& \begin{array}{c}{W}_{j}\\ \vdots \end{array}\end{array}\end{array}\end{array}\\ \begin{array}{cc}\begin{array}{c}\begin{array}{cc}\begin{array}{c}inf\\ \vdots \end{array}& \begin{array}{c}\ddots \\ \ddots \end{array}\end{array}\\ \begin{array}{cc}\begin{array}{c}{W}_{j+1}\\ \vdots \end{array}& \begin{array}{c}\ddots \\ \cdots \end{array}\end{array}\end{array}& \begin{array}{c}\begin{array}{cc}\begin{array}{c}\ddots \\ \ddots \end{array}& \begin{array}{c}\ddots \\ \ddots \end{array}\end{array}\\ \begin{array}{cc}\begin{array}{c}\ddots \\ \cdots \end{array}& \begin{array}{c}\ddots \\ \cdots \end{array}\end{array}\end{array}\end{array}& \begin{array}{cc}\begin{array}{c}\begin{array}{cc}\begin{array}{c}0\\ \ddots \end{array}& \begin{array}{c}\ddots \\ 0\end{array}\end{array}\\ \begin{array}{cc}\begin{array}{c}\ddots \\ \cdots \end{array}& \begin{array}{c}\ddots \\ \cdots \end{array}\end{array}\end{array}& \begin{array}{c}\begin{array}{cc}\begin{array}{c}\ddots \\ \ddots \end{array}& \begin{array}{c}inf\\ \vdots \end{array}\end{array}\\ \begin{array}{cc}\begin{array}{c}0\\ \cdots \end{array}& \begin{array}{c}{W}_{j+1}\\ \vdots \end{array}\end{array}\end{array}\end{array}\end{array}\right]$$

where 0 means the node correspond itself, *inf* is the feature of + ∞ in length, ${W}_{j}$ denotes the four types of interaction in this paper.

**Fig. 1:**
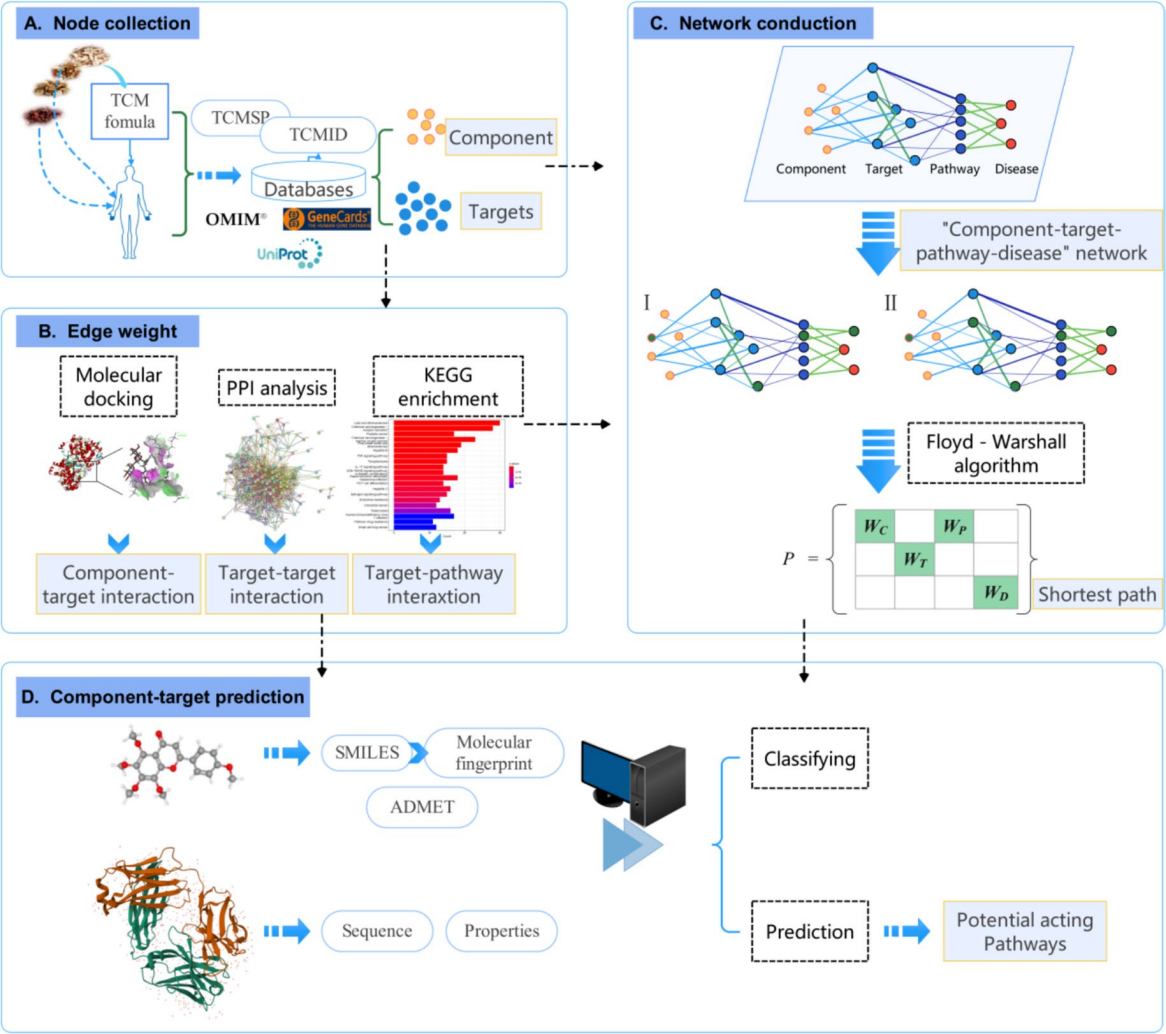
Process of machine learning-assisted network pharmacology analyzing and the construction of compound-target interaction predicting model. **A** Data for analysis mainly source from open access database. **B**, **C** For each TCM formula, the CTPDN network organized by 4 types of nodes and 4 levels of interactions. The value of each compound to the disease is shown as shortest path in CTPDN, which has two forms including compound-target-pathway-disease, and compound-target-target-pathway-disease. **D** A feature-based method for predicting compound-target interaction was developed by machine learning, and the key target from potential acting pathways were tested

In order to simplify the question for capturing the candidate functional compound from TCM, thought of dynamic planning was employed for identifying the path and its sum length through CTPDN. Floyd-Warshall algorithm is usually applied for solving multi-source issues in dynamic planning. Considering about the constructure of CTPDN, there are two kinds of approach to walk the path, which are shown in Fig. 1C. The core equation of Floyd-Warshall algorithm is shown as below:

5 $${P}_{k}=\text{min}(\sum ({N}_{c}, {N}_{d}))$$

where *N*_*c*_ denotes arbitrarily candidate compound as the beginning node for the path, *N*_*d*_ denotes the disease as the end node. We concatenate *P*_*k*_ as path length, recording every node along the shortest path between start node and final node. That are two cases of the shortest path, for example the compound-single target-pathway-disease, and compound-target group-pathway-disease.

### Prediction of compound-target interaction according to the feature of target and molecular fingerprint of compound

#### Feature-based compound-target interaction analyses

The descriptors of compounds were formed as two parts, one was molecular fingerprint descriptors, and the other was the prediction value of absorption, distribution, metabolism, excretion and toxicity (ADMET). Firstly, canonical SMILES of each core compound was collected from PubChem, then they were transferred into MACCS and ECFP6 separately by RdKit in Python 3.9.1. ADMET model on DS 4.0 was performed to access the evaluate the drug-like properties of candidate key compounds mentioned. Total 5 inspects were taken into account, including aqueous solubility (AS), blood–brain barrier (BBB), Cytochrome P450 2D6 inhibition (CYP2D6), hepatotoxicity and plasma protein binding (PPB).

As for the descriptors of core target were divided into two different types, which were represented of full length sequence and of Motif sequence. Protein property parameters including the number of diverse amino types, theoretical pI, extinction coefficients, estimated half-life, instability index, aliphatic index and grand average of hydropathicity. Briefly, the FASTA sequence was collected from UniProt, and the Motif was analyzed by MEME Suite. Then the ProtParam platform was introduced to calculate the property parameters. There were 4 datasets for generating the prediction model according to the permutation and combination of compound fingerprint and type of protein sequence. They could be summarized as MACCS-target full length sequence (MF), MACCS-target functional length sequence (MM), ECFP6-target full length sequence (EF), and ECFP6-target functional length sequence (EM).

The CTI pairs of datasets were formed as same as the compound-target interaction predicted in molecular docking. All pairs no matter was positive or negative results were collected. The positive result presented as “1”, the negative result presented as “0” shown in response value in the dataset. Then the descriptors of compound and corresponding target were listed as features. After deleting all the features in same, the 4 kinds of dataset were prepared to be modelled with permutation and combination of two compound descriptors and two target descriptors.

As the dataset was heterogeneous which could cause data noise, normalization was adapted into the dataset before the model construction. Here we selected Adaboost as classification algorithm for predicting CTI, some other kinds of classical classification algorithms were introduced for comparing in this study. Adaboost is one of the most popular algorithms for generating and boosting ensembles as its adaptability and simplicity.

The core formula for this algorithm was below:

6 $$f(x)=sign(\sum_{k=1}^{K}{\alpha }_{k}{G}_{k}(x))$$

where *k* means the number of weak classifier, *G*_*k*_(*x*) denotes the k-th weak classifier, and *α*_*k*_ denotes the weight of *G*_*k*_(*x*).

To avoid model overfitting, tenfold cross validation was adopted before training. Then the parameters were set as: max number of splits was 20, learner number was 30, and the learning rate was 0.1. Precision, recall, F1 score, AUC and MCC were chosen to be the model performance value. Their calculation equation was shown as below:

7 $$Precision=\frac{TP}{TP+FP}$$

8 $$Recall=\frac{TP}{TP+FN}$$

9 $$F1 score=2\times \frac{Precision\times Recall}{Precision+Recall}$$

10 $$AUC=\frac{{\sum }_{in{s}_{i}\in positive}{rank}_{{ins}_{i}}-M\times (M+1)/2}{M\times N}$$

11 $$MCC=\frac{TP\times TN-FP\times FN}{\sqrt{(TP+FP)\times (TP+FN)\times (TN+FP)\times (TN+FN)}}$$

where TP, TN, FP, FN means the true positive, true negative, false positive, and false negative, respectively. AUC denotes the meaning of the area under the ROC curve.

#### Identification of compound-target interaction by Adaboost model

With the result from previous prediction of key targets from pathways were selected according to the pathways from KEGG and published papers. After the same processing steps, the sequences of selected key targets were formed. Model was adapted for computing the drug-target interaction between key compound and key targets (see Fig. 1D).

### Molecular docking and dynamics simulation

The crystal structure of targets (PI3K, AKT1, CAT, and STAT) were obtained from the Research Collaboratory for Structural Bioinformatics Protein Data Bank (RCSB PDB). The 3D structure of compounds was obtained from PubChem (https://pubchem.ncbi.nlm.nih.gov/). Docking simulations between the compounds and targets were performed by “-CDOCKER” to analyze the conjugation between the ligand and the protein. All parameters were set as default. The molecular docking was performed on Discovery Studio 4.0.

The best molecular docking conformation, recognizing the lowest docking energy of target-compound complex, was selected as the initial conformation for dynamic simulations. GROMACS 2018.33 was used to perform simulations. The CHARMM force field was constructed using the Biomolecular Modeling and Simulation Platform (https://charmm-gui.org/). The simulation system was filled up with water by TIP3P model in a cubic box. Dynamics simulation was carried out for 50 ns with a time step of 0.1 ns. All simulation were run under isothermal isobaric ensemble with thermodynamic temperature of 310.15 K and a pressure of 101.325 kPa. The temperature and pressure are controlled by the V-Resale and Parrinello Rahman methods. Lennard Hones function calculated thr van der Waals force while the non bonding cutoff distance was set as 1.2 nm. The Lincs algorithm constrains the bond lengths of all atoms, and the particle grid Ewald (PME) method calculated short-range electrostatic interactions. Results were visualized using PyMol software.

### Western blotting

The liver tissue was lysed by RIPA lysis buffer containing protease inhibitor and phosphatase inhibitor. The total protein concentration was determined by the BCA protein quantification kit (NCM, WB6501). An equal amount of protein (35 μg) was loaded onto 10% SDS-PAGE and then transferred onto 0.44 μm PVDF membranes. The membranes were blotted with 5% skimmed milk powder in TBST buffer for 120 min at room temperature and the incubated at 4℃ overnight with primary antibodies: anti-AKT1 (1:8000, Proteintech, 80,457–1-RR), anti-STAT1 (1:8000, Proteintech, 10,144–2-AP), anti-Catalase (1:10,000, Proteintech, 21,260–1-AP), anti-pAKT1 (1:2500, Immunoway, YM8304), anti-β-actin (1:10,000, Immunoway, YM3028). Then, the membranes were incubated with horseradish peroxidase (HRP)-conjugated secondary antibodies (1:10,000, RS0001; 1:10,000, RS0002) for 60 min at room temperature. The total protein was normalized to β-actin. The bands’ gray intensity was analyzed by ImageJ (NIH image software).

### Statistical analysis

**Fig. 2:**
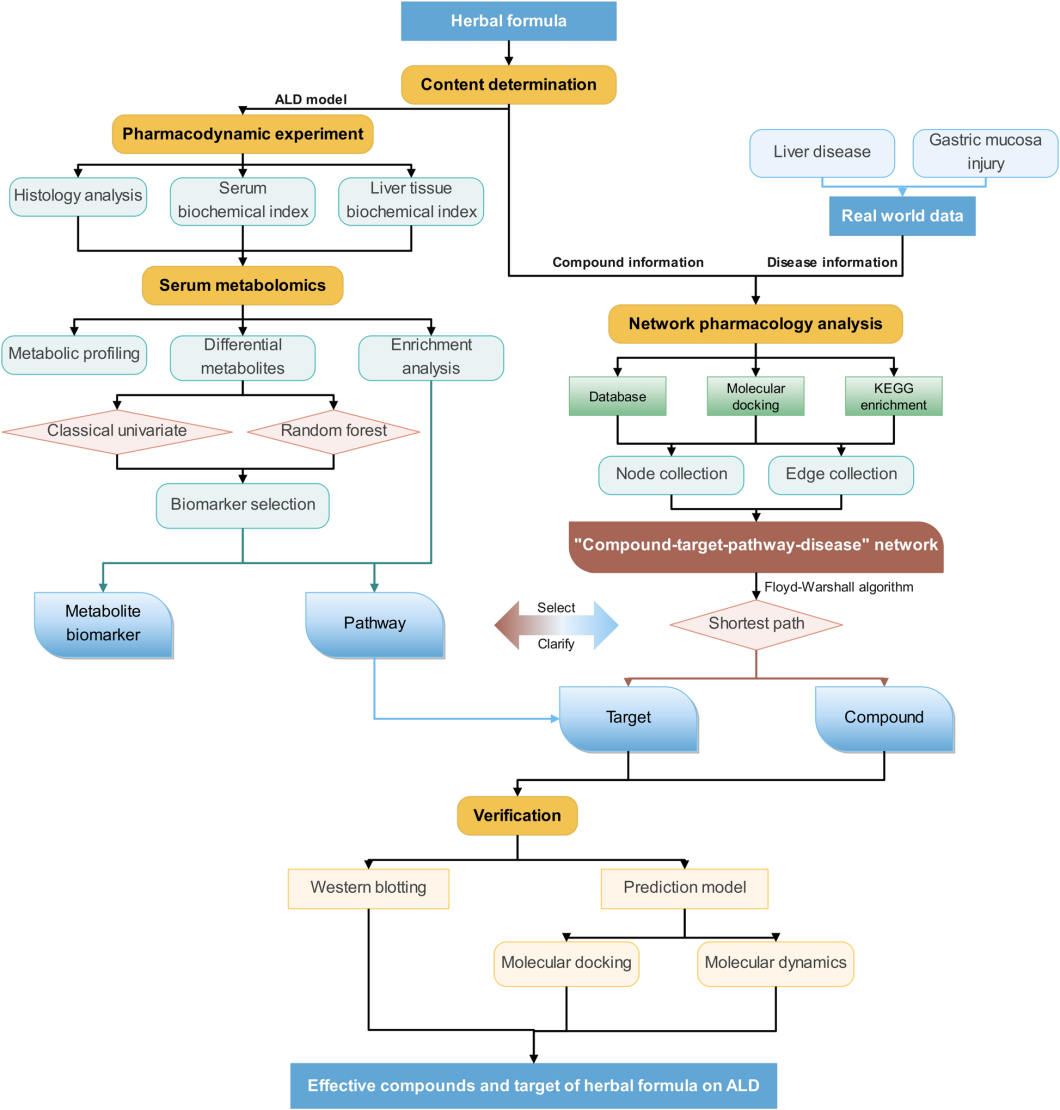
Workflow for identifying effective compounds from herbal formula on ALD

The work flow of this study are shown as Fig. 2. Results of pharmacology part were analyzed by one-way analysis of variance (ANOVA) followed by the least significant difference (LSD) method and Dunnett’s test for multiple comparisons. *p* < 0.05 was considered statistically significant. Statistical and data analyses was performed using SPSS software (Version 20.0; SPSS Inc., Chicago, IL, USA).

## Results

### Quantification of main components of BWG

**Fig. 3:**
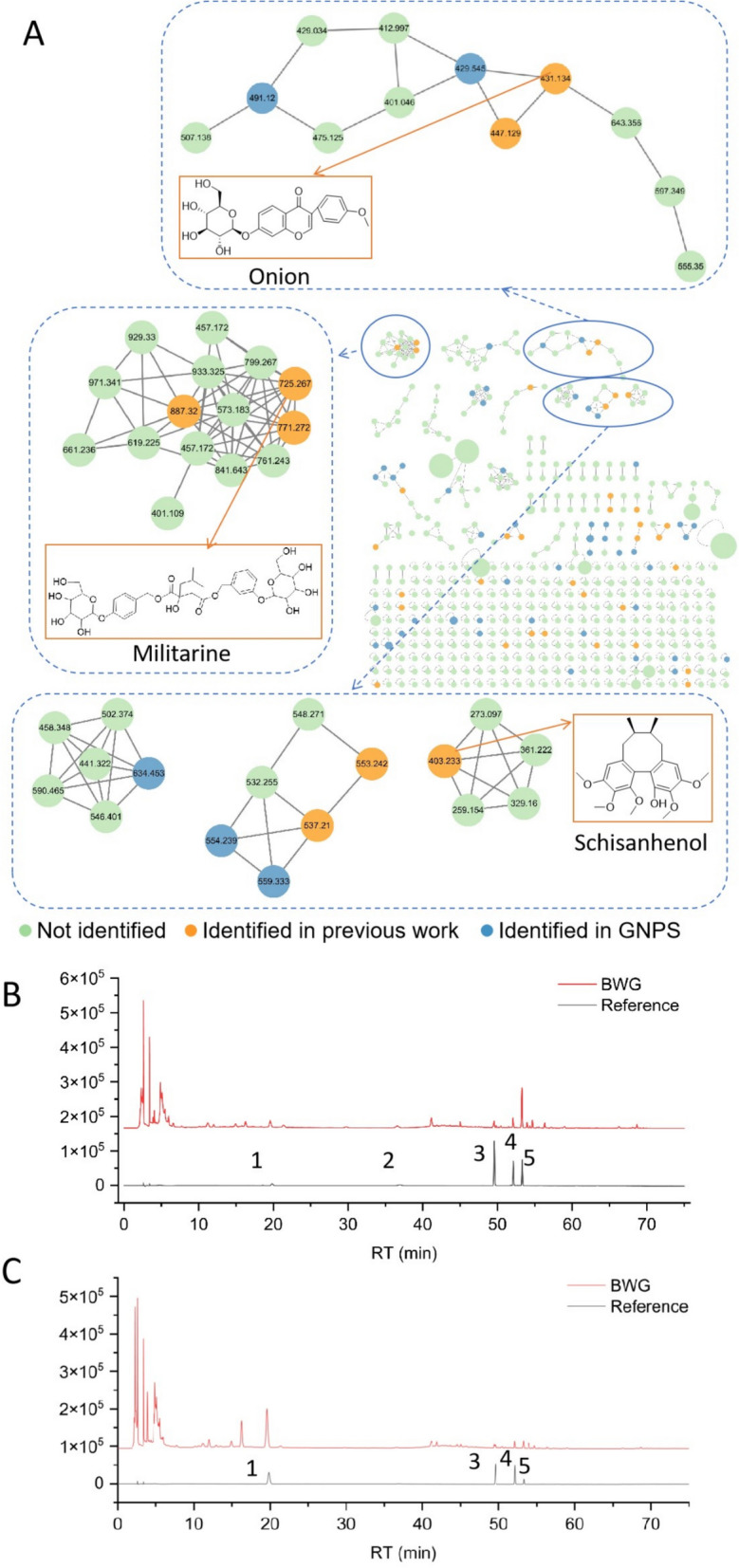
**Main components of BWG. A** Molecular network analysis. Characteristic components of main substances included militarine, onion, and schisanhenol. Green nodes stood for the not identified compounds, while orange nodes stood for the identified compounds in team’s previous work, and blue nodes stood for the identified compounds in GNPS. **B** HPLC chromatogram of content determination for five compounds of BWG in 254 nm. Peak No.1 ~ peak No.5 were hesperidin, militarine, formononetin, nobiletin, and schisandrin, respectively. **C **HPLC chromatogram of content determination for five compounds of BWG in 283 nm. Peak No.1 ~ peak No.5 were hesperidin, militarine, formononetin, nobiletin, and schisandrin, respectively

Molecular network of BWG was generated by GNPS based on the similarity of compound fragmentation patterns. This network consisted 17 clusters (number of nodes > 3) and 267 single nodes. A total of 82 compounds (35 compounds were identified in team’s previous work) were identified in molecular network analysis. All nodes were categorized according to their compound classes which were flavonoids, lignans, glucosyloxybenzyl 2-isobutylmalates, and phenolics. The representative chemical structures of clusters of flavonoids, lignans and glucosyloxybenzyl 2-isobutylmalates were shown in Fig. 3A.

To quantify the main components of BWG, content of total phenolics, total flavonoids, and five compounds were measured. Additionally, total sugar and total triterpenoids were also measured. The previous studies indicated that saccharides and triterpenoids exhibited a wide spectrum of in vitro and in vivo pharmacological effects [36, 37], which were selected for further investigation in the present study. The content of main components was shown in Table 1 and Fig. 3B, C.

**Table 1:** The main compounds of BWG and their contents

Conponent	Content
Total phenolics (mg GAE/mL)	0.0306 ± 0.0003
Total flavonoids (mg RE/mL)	0.0426 ± 0.0020
Total sugar (mg Glc/mL)	0.0283 ± 0.0004
Total triterpenoids (mg OA/mL)	0.0614 ± 0.0056
Compounds	
Hesperidin (mg/g)	3.26 ± 0.01
Militarine (mg/g)	9.45 ± 0.27
Formononetin (mg/g)	0.11 ± 0.00
Nobiletin (mg/g)	0.24 ± 0.01
Schisandrin (mg/g)	1.23 ± 0.06

### BWG alleviates chronic alcohol-induced liver injury

**Fig. 4:**
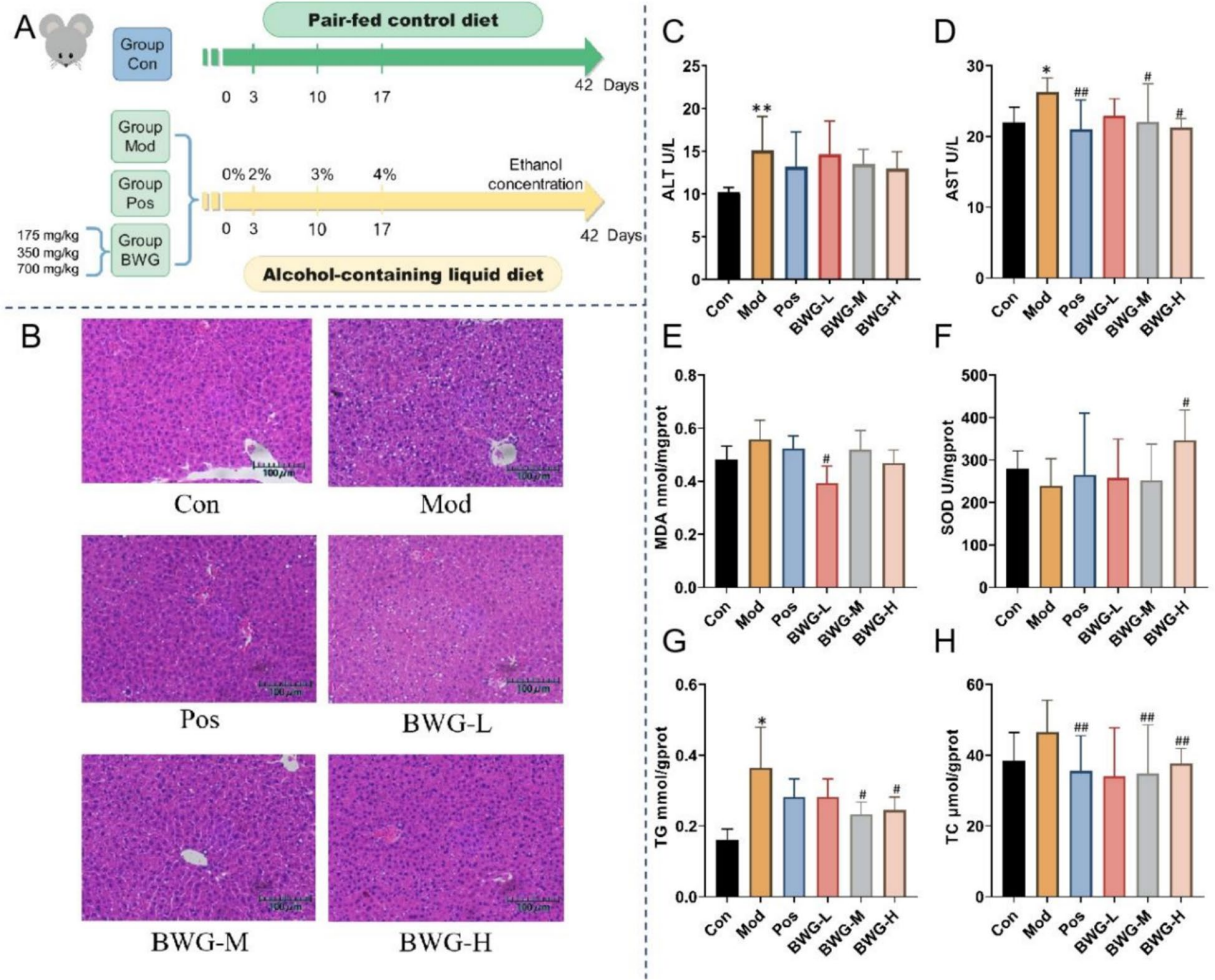
Protective effect of BWG on ALD. **A** Schematic diagram of the animal experimental procedure. **B** H&E staining (scale bar = 100 μm). **C**, **D** Serum ALT and AST. (*n* = 6) **E**–**H** Liver tissue biochemical indicators, which was MDA, SOD, TG, TC, respectively. (*n* = 6). All results were expressed as the mean ± SD. ^*^p < 0.05, ^**^p < 0.01, significantly different from Con group (Pair-fed); ^#^*p* < 0.05, ^##^*p* < 0.01, significantly different from Mod group

The chronic ALD mice model was established as team’s previous work and was concluded as Fig. 4A shown. Because of long-termed alcohol diet fed, body weight, the index of spleen and thymus were increasing (*p* < 0.05) in Pos group, while BWG group had the even value with Con group (Table S5). That indicated BWG may have little effect in immune system. Serum ALT and AST levels as well as H&E stains were examined to determine the degree of liver damage. The H&E staining showed the macroscopically observation of liver tissue in each group, and also provided the most intuitive evidence for ALD (Fig. 4B). Liver cells of Con group were typical normal liver cell morphology with uniform cytoplasm and clear nuclei. While the Mod group showed disordered arrangement of perivascular hepatocytes, shrunken hepatic sinusoids and cytoplasmic loosing of liver surface cells after intaking alcohol. According to the observation of these groups, the size of liver sinusoids of Pos group and BWG-L, BWG-M, and BWG-H groups were more normal in contrast with the Mod group, and the liver cells were arranged regularly. That proved BWG had the ability for alleviating ALD. According to that concept of preventive treatment of disease, TCM formula is able to strengthen body and preventing body from disease before it is able to take significant effect. Many changes could happen with alcohol intake, including the elevated level of ALT and AST in serum (Fig. 4C, D). Similarly, the increase of serum AST by alcohol expose were recovered by BWG (*p* < 0.05). Unfortunately, change of serum ALT was not as significant as AST, even though the clear downward trend could be observed. All these results of biochemical indexes firmed the concludes of the ALD model was successfully established and the protective effect of BWG. To further explore the potential mechanism of BWG on ALD, the relating biochemical indexes of lipid metabolism and antioxidant activity, such as TG, TC, MDA, and SOD, were tested either. As the results shown (Fig. 4E–H). Just same as the change trend in previous histopathological examination results, the levels of TG, TC and MDA of liver tissues were increased. BWG group were able to significantly decrease the changing trend (*p* < 0.05), except in MDA of liver tissue.

### Analysis of serum metabolites profiling and identification of biomarker of serum metabolite

#### Multivariate statistical analysis of metabolites profiling

Methodological examination was performed before the sample test. Under the negative and positive ion mode, the precision, repeatability and stability all met the requirements, which ensured this method suitable for the analysis. The results of methodological examination were shown in Table S6.

**Fig. 5:**
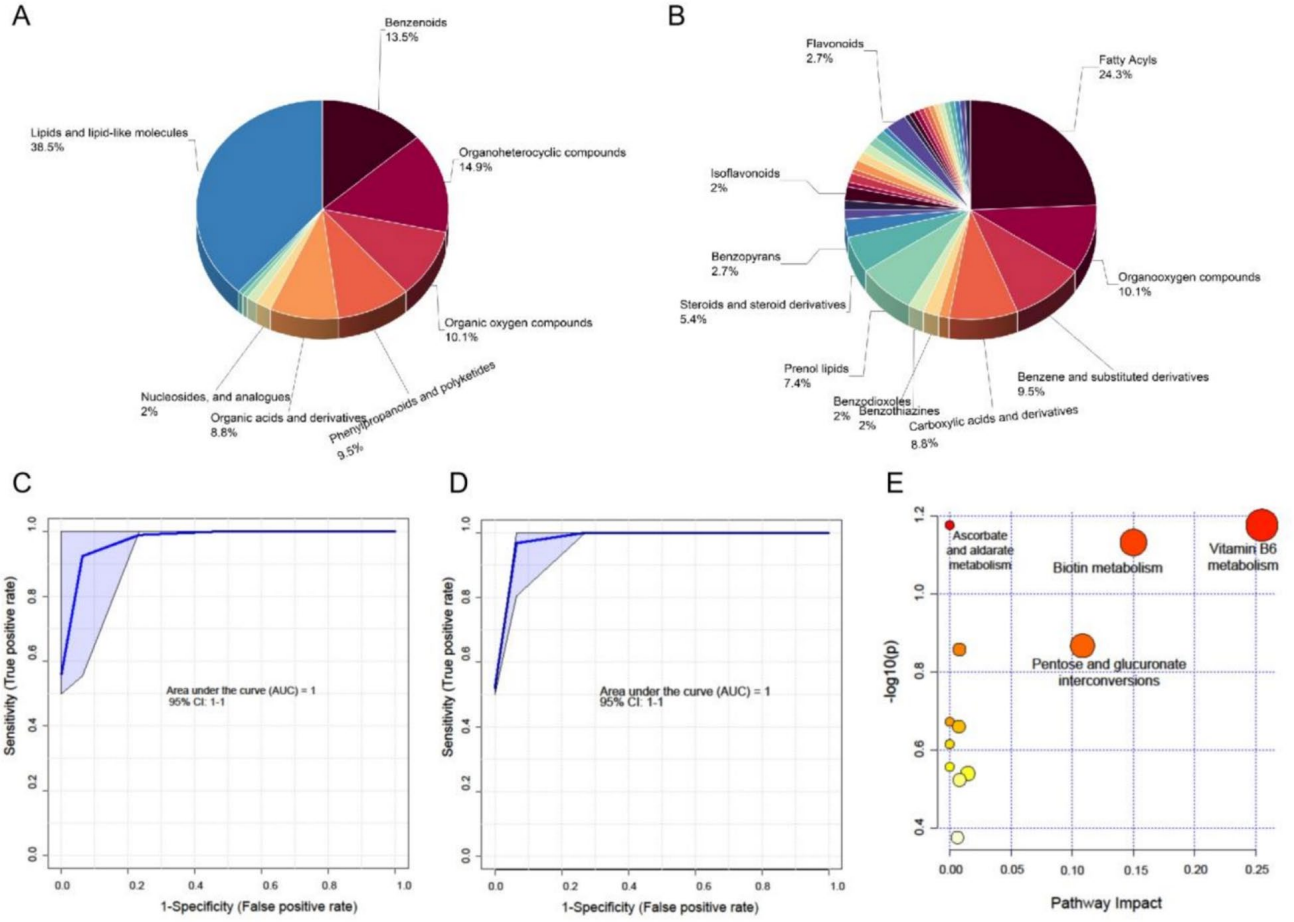
Analysis of metabolite profiling, identification of metabolite biomarker, and KEGG enrichment of differential metabolites. **A** Super chemical class of differential metabolites. **B** Main chemical class of differential metabolites. **C** ROC curve of 10 predicted metabolites biomarkers for distinguishing Mod group from Con group. **D** ROC curve of 10 predicted metabolites biomarkers for distinguishing BWG intervention from Mod group. **E** Results of enrichment pathways

Total 118,539 peaks in positive ion mode and 52,281 peaks in negative mode were detected. PCA and PLS-DA analysis were performed after data pre-processing, for example, deleting outlies and standardization. The results of PCA, PLS-DA showed that BWG intervention significantly affected serum metabolite profiles of ALD mouse (Fig. S8). With analysis for annotation of differential metabolites, 148 metabolites in the positive and negative ion mode were yielded (Table S7, Fig. S9–11). Among these differential metabolites, 78 metabolites were downgrade while 39 meta bolites were up graded. Moreover, with counting the types of these differential metabolites, lipids and lipid-like molecules, organoheterocyclic compounds, and benzenoids were the abundant type of metabolites (Fig. 5A, B).

#### Identification of biomarker metabolites

To further analyze the metabolites closely associating with ALD, the identification of biomarker was carried out by random forest (RF) analysis. The well-established RF analysis demonstrated excellent separation of Mod group from Con group (Accuracy = 98.5%, AUC = 1, 10 features), as well as the Mod group from BWG group (Accuracy = 100.0%, AUC = 1, 10 features). This result demonstrated the difference in serum metabolites that existed between Mod group and Con group, also between Mod group and BWG group. With more statistical analysis, 7 metabolites of the comparison different metabolites of previous groups in common were identified (Table S8).

#### Enrichment of metabolic pathways

All differential metabolites were performed functional analyses using KEGG metabolite library in order to understand how BWG influenced serum metabolite profiling better. Metabolic pathways with *p* < 0.05 and impact value > 0.1 were selected as potential metabolic pathways. Three related metabolic pathways were screened out (Fig. 5E), including Vitamin B6 metabolism, biotin metabolism, and pentose and glucuronate interconversions.

### Selection of key compounds and targets from shortest path of “compound-target-pathway-disease” network

#### Identification of nodes and edges of CTPDN

**Fig. 6:**
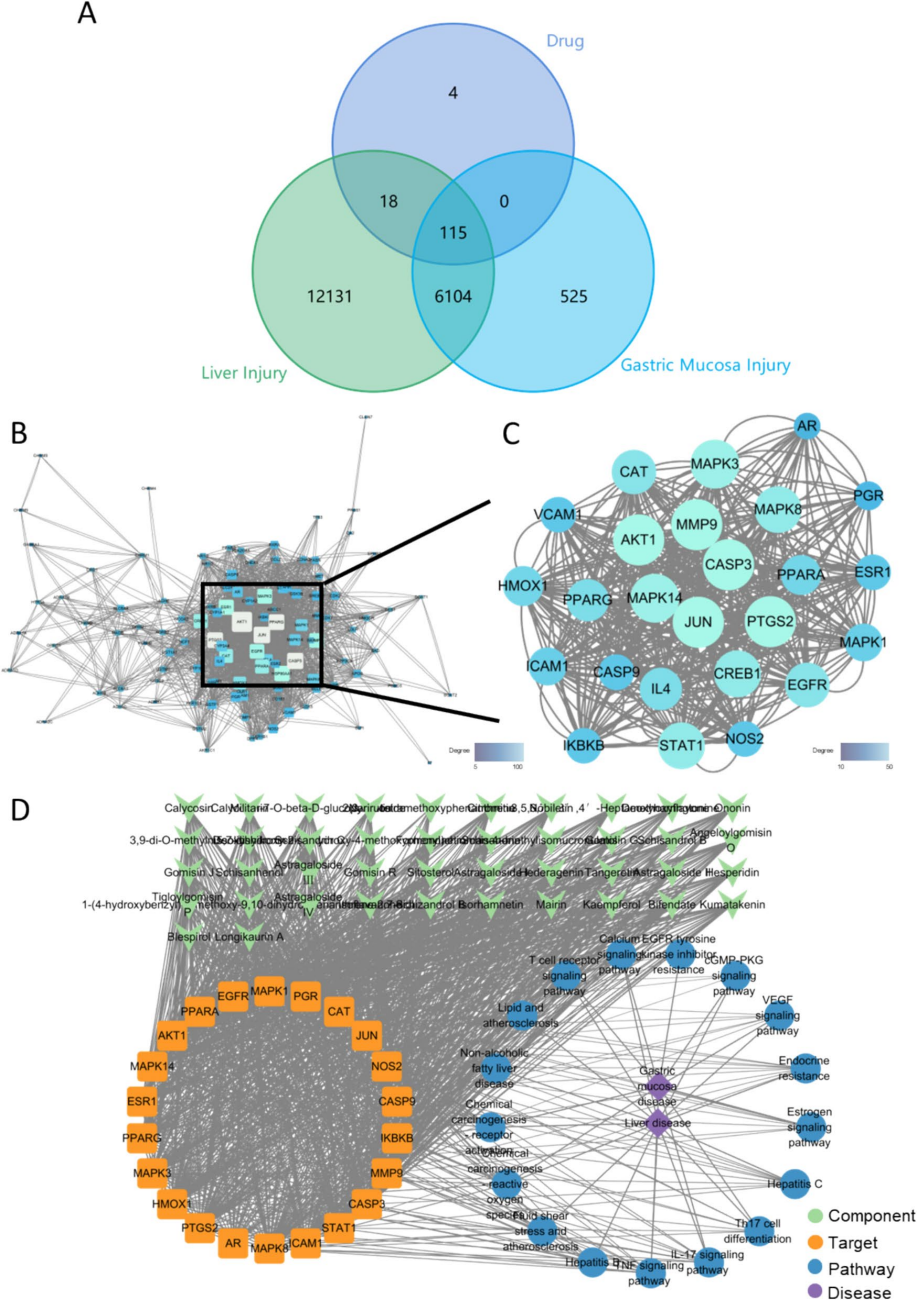
Basic analysis of CTPDN. **A** Venn diagram of diseases and TCM formula. **B** PPI analysis of intersection target points from Venn diagram. **C** Hub target group. **D** Graphic expression of CTPDN. Edges were weighted based on computational methods, shown by the width of lines in graph in addition

As for the bi-function of BWG, liver disease and gastric mucosa injury were all taken into CTPDN construction. Nodes of CTPDN were divided into 4 types, including compound, target, pathway and disease. A total of 42 compounds were screened from databases and publications, 145 relevant targets were identified at the same time. Further, with 18,368 targets from liver disease and 6744 targets from gastric mucosa, the Venn diagram of intersection targets in drug and disease was identified (Fig. 6A). 115 targets in same were collected as potential therapeutic targets as a result. PPI analysis showed that there were 1080 edges around 114 relative targets (Fig. 6B). Then the targets were divided into different sizes by their degree value, the brighter and bigger the node performing, the more important the node stood for. MCODE app was employed to determine highly interconnected subnets. 6 core groups were clustered eventually. In case of the score of cluster one was much higher than others, as well as to simplify the problem, the cluster one was chosen to be the only cluster standing for the hub target group (Fig. 6C). As the clustering showed (Table S9), MMP9 was believed to play an indispensable role in this sub-network, AKT1 and JUN were even vital to the sub-network as well according to their remarkable score.

GO functional and KEGG signal enrichment were performed by “Bioconductor” package so as to know about relative targets better, and also to collect the pathway node. The results were screened according to the *p* value (*p* < 0.01) also taking effect on various pathways. In summary, 1249 on biological process, 59 on cell component, 140 on molecular function, and 139 pathways were enriched, and top 10 of them were summarized (Table S10). Themes for discussion, the particularly significant pathways (*p* < 1 × 10^–8^) were chosen to analysis further, then those pathways without clustering gene were deleted. The contact between pathways and disease was established by the publications, 17 pathways were finally screened to conduct the network (Table S11). Chemical carcinogenesis-receptor activation (KEGG ID: hsa05207) and chemical carcinogenesis-reactive oxygen species (KEGG ID: hsa05208) had very close relations with liver disease and gastric mucosa focus on how the vital target and important pathways influence the BWG hepatoprotective effect. Taken together, the findings demonstrate that BWG could influence the body by multi-compound and multi-target acting, while it was still be a trouble in deeper and clearer articulation of mechanisms.

Talking about the edges collection was simpler than nodes collection. There were 4 types of node relation, which were CTI, TTI, TPI, and PDI. CTI was conducted and weighted by the results from molecular docking. In molecular docking, target proteins without unique ligands were deleted for further analysis in advance, otherwise could lead a tough docking process and failing docking result, which including CREB1, IL4 and VCAM1. Total 43 compounds and 22 targets were participated in molecular docking analysis for the conduction of CTPDN eventually. Even though some compounds, like bibenzyls from *Bletillae Rhizoma *etc*.*, were hard to build interaction with the target better than the origin unique ligands, they were still kept to verify the possibility for the interaction. It was worthy to discuss that most dockings with EGFR, CASP9, IAM1 and JUN were better than the original one. At the same time, the success rate was around 20% to 35% in whole, while narirutin and nobiletin both had the highest rate of 36.36%. Benefiting from the PPI, TTI of this study were weighted. As for the TPI and PDI in this study, it seemed to be a exist or not question. All in all, CTPDN was formed to dig how BWG take effect exactly.

#### Value key nodes from CTPDN according to shortest path method

**Fig. 7:**
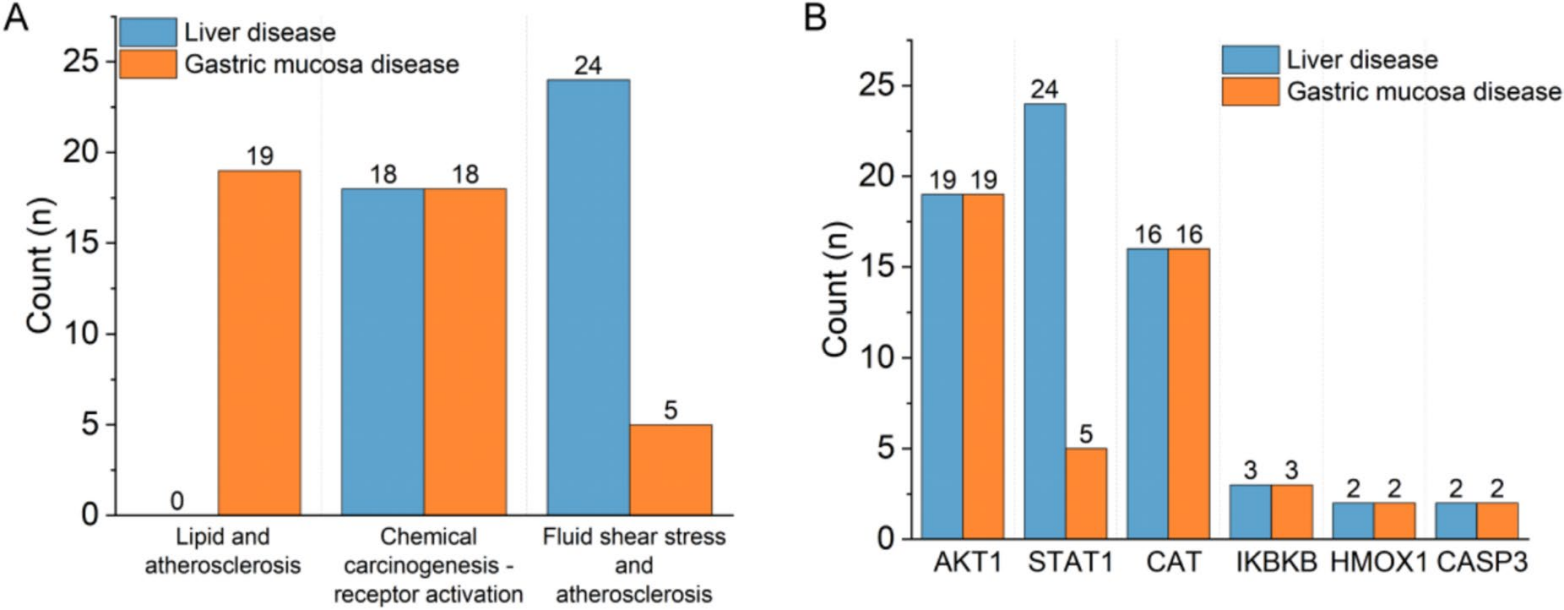
Count of key pathways and targets. **A** Associated pathways. **B** associated targets

Compounds take effects mainly by combined with targets, which mostly are proteins. Then targets together make a difference on molecular level to perform on the body, and taking effect eventually. Therefore, CTPDN was modeled for valuing compound, target as well as the pathway along the dynamic gridded path. CTPDN was a bi-directed network, herein the Floyd-Warshall algorithm was served to finding the shortest weighted way, that was the reverse direction of how compounds taking effects on body. CTPDN of BWG taking effect on liver disease and gastric mucosa was shown as Fig. 6D. The shortest path length of each compound was shown in Table S12, also the count of key pathways and targets were shown in Fig. 7.

The shortest way in CTPDN mainly divided into three types, AKT1, lipid and atherosclerosis, CAT and chemical carcinogenesis—receptor activation, and AKT1 or HMOX1 with STAT1 together acting on fluid shear stress and atherosclerosis. This indicated AKT1 was a vital target to BWG treating liver disease, which was corresponded to the MCODE clustering result previous. The shortest path length participating in following analysis was the average length of compound to each disease. The length of shortest path of each compound was about 1.60 in BWG. It was interesting that the similar chemical structure the compound shared, the no significant difference the shortest path was tend to be. It may be caused by the molecular docking mode that achieved mostly based on the chemical structure of each compound [9]. Moreover, some targets connected to the pathway were not the core targets of the pathway, so that more validation experiments about core target of pathway were needed for prove the acting pathway to clarify the mechanism.

Talking about the compounds having close relation with liver disease, they could only act at chemical carcinogenesis-receptor activation and fluid shear stress and atherosclerosis pathway according to the CTPDN. To efficient discovery and evaluate the medicinal possibility of compounds from BWG, ADMET prediction was performed. Prediction of ADMET properties were performed on DS 4.0, and the results shown (Table S12 and Fig. S12). Moreover, potential effective compounds were selected according to the results of compounds consisted in BWG, which were identified by UHPLC-MS/MS from our previous work [11]. Six effective compounds including 2,3,4,7-tetramethoxyphenanthrene, narirutin, astragaloside IV, gomisin J, schisandrin, and schisandrol B were picked out as potential effective compounds.

### Effective compounds of BWG alleviates ALD by modulating PI3K-AKT1 signaling pathway

#### Establishment of compound-target interaction predicting model

As indicated above, we envisioned and managed a model for predicting compound-target interaction. In this model, relation between compound from BWG and core targets of pathway were predicted. Feature-based method is one of the most important type for predicting CTI based on machine learning [38]. It was represented by vectors of descriptors which were encoded as corresponding features.

Chemical structure of compounds is believed to have high relativity with their bioactivity, which consequently influences their physiological functions in both herbs and human body [39]. MACCS and ECFP6 are the useful molecular fingerprint descriptors for the presence or absence of certain molecular substructures. That are also thought to be the foundation in compound similarity searches and clustering, then to predict target [40]. In this part, total 966 compound-target pairs from previous molecular docking part, including positive and negative samples, were taken into account for generating compound-target interaction dataset. The features of dataset mainly generated by compound descriptors (fingerprint descriptors, and ADMET properties) and protein descriptors (sequence and protein properties). Even though compounds got their functional value in network, more drug-likeness properties, like ADMET, should be considered into valuing and ranking compounds. Taken together, the compound fingerprint described by MACCS were finally formed dataset with 201 features, the one described by ECFP6 was formed with 2082 features.

**Fig.8:**
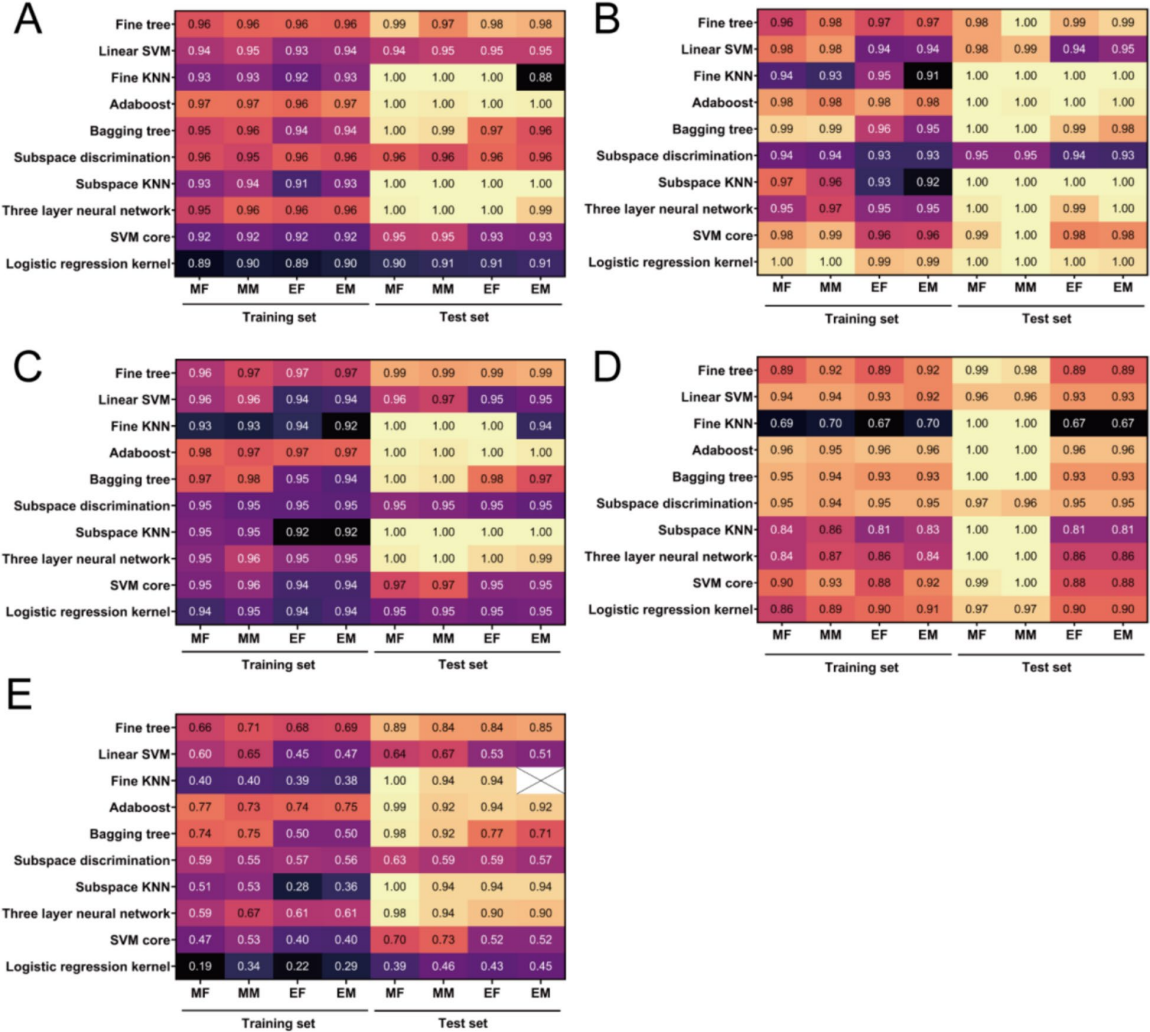
Evaluation results of classification model. **A** Precision value. **B** Recall value. **C** F1 score. **D** AUC. **E** MCC

It is a common practice to take CTI as a classifier issue. Therefore, AdaBoost was employed for solving this kind of issue. AdaBoost is the first practical boosting classifier which combines multiple base classifiers to produce a strong classifier that achieves accurate classification [41]. With comparison the classification model developed by different algorithms of these dataset, the best model was supposed to be EF trained by Adaboost. It was obvious that model developed by Adaboost performed best among all the models because of the five values for evaluating the model (Fig. 8). That precision, recall, F1 score, AUC and MCC of this model in training set were 0.96, 0.98, 0.97, 0.96 and 0.74, respectively. While in test set the value were 1.00, 1.00, 1.00, 0.96, and 0.94, respectively. The outstanding performance of prediction model provided a firm base for further study about targets from potential acting pathway.

#### Screening potential mechanisms via compound-target interaction prediction

Having demonstrated the well-trained CTI prediction model, we next tested core targets of key pathways (Fig. 7) to lock down the actual acting pathway of BWG among the many possible pathways as well as the targets. The core targets of signaling pathways were selected according to KEGG records. Since PI3K-AKT signaling pathway is essential and primary to chemical carcinogenesis-receptor activation as well as fluid shear stress and atherosclerosis pathway, seven targets consisted of upstream and downstream pathways were taken into considered to provide another view of mechanism of BWG on liver disease. As the same process of predicting the compound-target interaction, the binding relations of compound and 7 core targets were tested by the previous model. The results in Table 2 showed that BWG had great potential in effecting PI3K-AKT signaling pathway due to their higher rate of success CTI.

**Table 2:** Summary of targets from potential acting pathways

Target	Uniprot ID	Rate of successful CTI (%)
NF-κB	Q8N5F7	78.57
IL-1	P01583	83.33
IL-6	P05231	100.0
TNF-α	P01375	85.71
IL-1β	P01584	95.24
PI3K	Q8NEB9	88.10
AMPK	Q9UGI9	88.09

#### Binding mode of effective compounds with key targets

Molecular docking was taken for understanding the binding mode of effective compounds with targets. This method was to be the validation of results of serum metabolomics and also the compound-target interaction prediction. Therefore, PI3K (PDB: 3LS8), ATK1 (PDB: 1UNQ), CAT (PDB: 1DGF), and STAT1 (PDB: 1YVL) were used to calculate the binding affinity of six effective compounds.

**Fig. 9:**
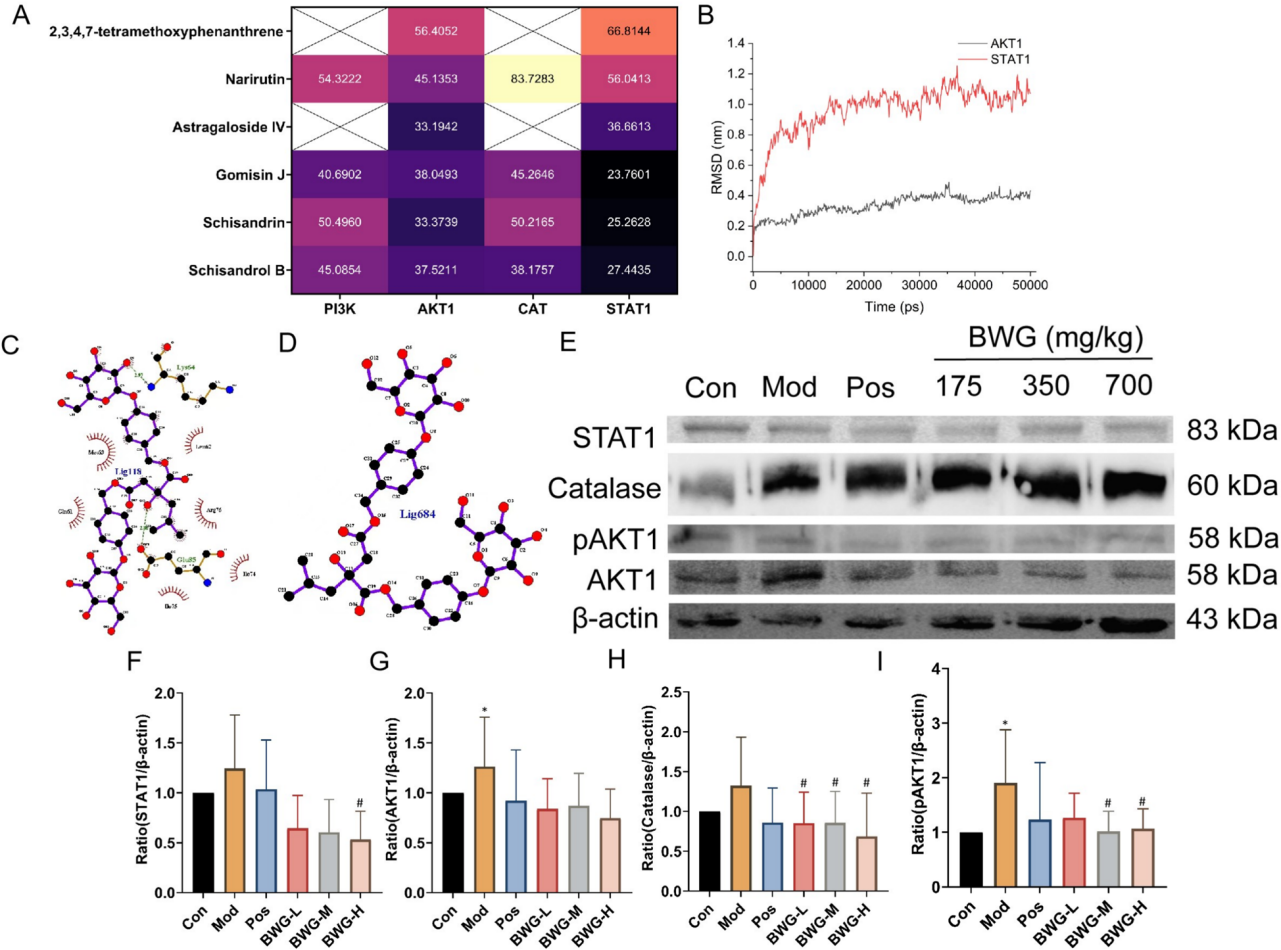
Results of molecular docking and western blotting of key targets. **A** Value of “-CDOCKER INTERACTION ENERGY” of effective compounds with key targets. “-CDOCKER INTERACTION ENERGY” only contains the interaction energy between compound and target, while the intramolecular energy of the compound is not included. **B** RMSD of AKT1—2,3,4,7-tetramethoxyphenanthrene complex (black) and STAT1—2,3,4,7-tetramethoxyphenanthrene complex (red). **C** Interaction of AKT1 and 2,3,4,7-tetramethoxyphenanthrene at 50 ns of dynamic simulation. **D** Interaction of STAT1 and 2,3,4,7-tetramethoxyphenanthrene at 50 ns of dynamic simulation. **E**–**I** The protein expression and corresponding statistical analysis of STAT1, AKT1, Catalase, and pAKT1, respectively. (*n* = 3) All results were expressed as the mean ± SD. ^*^p < 0.05, ^**^p < 0.01, significantly different from Con group (Pair-fed); ^#^*p* < 0.05, ^##^*p* < 0.01, significantly different from Mod group

The docked results revealed that all compounds fitted via a minor groove with binding affinity over −23 kJ/mol (Fig. 9A, Fig. S13–S16). It was found that hydrogen bonds were the most significant feature of bonding type, and some other interactions, for example, the hydrophobic bond and salt bridge, were also observed to be supportive bonding type for the effective compound and target.

To understand the protein–ligand interaction of key compounds and protein, 2,3,4,7-tetramethoxyphenanthrene, which had the prominent binding energy to the valuable targets, was chosen to perform dynamics simulation with AKT1 and STAT1. Root mean standard deviation (RMSD) is the important profile for monitoring the equilibrium process of system and the stability of protein structure after ligand binding [42]. As the results shown in Fig. 9B, the AKT1—2,3,4,7-tetramethoxyphenanthrene complex exhibited an RMSD range of 0.0050 to 0.4886 nm (average 0.3365 nm), while the STAT1—2,3,4,7-tetramethoxyphenanthrene complex showed an RMSD range of 0.00050 to 1.2525 nm (average 0.9619 nm). These results showed consistent RMSD patterns, which suggested the relatively stable interaction of two complex. The results also indicated that AKT1—2,3,4,7-tetramethoxyphenanthrene complex was more stable compared to the STAT1—2,3,4,7-tetramethoxyphenanthrene complex. To be specific, 2,3,4,7-tetramethoxyphenanthrene complex had a strong hydrogen bond with amino acid residue of AKT1 (Lys(64) and Gul(85)), while hydrophobic interactions were the another characteristic of this complex. Meanwhile, there was none interactions of 2,3,4,7-tetramethoxyphenanthrene with amino acid residue from STAT1.

#### Expression result of key target

AKT1, CAT and STAT1 had the highest count among key targets selected by the shortest path of CTPDN. To demonstrate the generality of this predictive analysis strategy and activation of approach, the expression of high appearance target from shortest path were verified. The results showed that the expression of AKT1, pAKT1, Catalase and STAT1 were significantly decreased with the effect of BWG. These observations suggested that the prediction strategy applied in BWG could confirm the effectiveness on predicting at core target and the pathway. That indicated BWG was more likely acting at PI3K-AKT signaling pathway.

## Discussion

BWG has been designed according to the real-world data, which is thought to have bi-function [11, 43]. Just after the modification of extraction methods, and content determination of characteristic components, the protective effects and identification of effective compounds were carried out in this study. Here, we performed in vivo as well as in silico experiments to select effective compounds and to clarify the protective effect of BWG on ALD.

To have a better understanding of the chemical composition of BWG, molecular network and quantification of main components were performed. Compared with previous analysis results, 47 new compounds were identified by molecular network analysis. Whereas, some compounds, including 2,3,4,7-tetramethoxyphenanthrene, were not visualized in the network, and some compounds of same class did not cluster. These results indicated molecular network was a powerful assisting tool to discover new compounds, yet traditional analyses were need as well. The composition and the content of main components were determined which was capable to provide basis for the mechanism study for BWG on ALD.

Defining the target and mechanism of BWG action is a fundamental and critical step in screening effective compounds. Inflammation, disorders of lipid metabolism and oxidative stress are the typical characteristic of ALD, which fatty liver disease accompanying with inflammation is the very early pathological feature [44]. In the part of pharmacodynamic evaluation, BWG has been proved to effect on maintaining the relative stable state of indicators of liver damage (ALT, AST), antioxidant (MDA, SOD), and lipid metabolism-relating (TG, TC). Meanwhile, this effect of BWG led to a reduction in the degree of hepatocyte damage, which was also reflected in the reduction of liver organ index. As BWG may has bi-function of protective of liver and gastric mucosa, the condition of stomach was observed. The stomach organ index was significantly reduced when BWG intervened (*p* < 0.05) (Table S5), that indicated the potential protective effect of gastric mucosa. Yet the Mod group showed no difference in stomach organ index compared with Con group. This results may contribute to the animal model established by Liber-DeCarli liquid feed [45, 46], that need very long-term experiment period. Considering about the protective effect of BWG on ALD as well as the potential protective effect on gastric mucosa, we then took serum metabolomics and network pharmacology analysis to demonstrate the bi-function of BWG.

Metabolites in peripheral blood are able to act on all organs of the body, including the liver and stomach. Changes in abundance of serum metabolites can accelerate the recovery of tissue [47]. On the one hand, the abundance of serum metabolites is influenced by the effect of the drug acting on metabolic enzymes and affecting their production or degradation. On the other hand, the drug may competitively bind to the target, allowing metabolites to accumulate. Therefore, analyses of differential serum metabolites are essential for clarifying BWG perturbations. To extract well-behaved peak features and stable peak groups from untargeted metabolomics with massive information, reliable data preprocessing methods were adapted [48]. As a result, total 148 differential metabolites, 7 potential biomarkers, and 3 metabolism pathways were identified. Random forest was performed to select features from differential metabolites which may provide insights to multiplexed serum metabolites biomarker for facile screening in diagnostic classification of BWG intervention on ALD [49]. Acetyl tributyl citrate and calcitroic acid were the most valuable differential metabolites in biomarker classification. They are recognized to relate lipid metabolism which was normally occurred in liver and gastritis that supports the accuracy and reasonableness RF classification model [50–52]. Though differential metabolites and related metabolism pathways were identified, more evidences are needed to fulfil the proposal of BWG acting mechanism.

Because of the several enrichments of metabolism pathway and differential metabolites with vary functional roles, it remains a challenge to decide the most valuable pathway of BWG intervention. Combination with computational methods like network pharmacology offers a rapid way to study about mechanism of drug acting. Degree value and other indicators like closeness centrality are the most common evaluation for network topological analysis. These indicators kind of overlook the importance of edges. Besides, previous study is likely to focus on the full list results, lacking of the attention of local information about specific compound, target or pathway [12]. Herein, shortest path of CTPDN has been described based on the nodes and weighted edges instead of just caring about the position of nodes in the network [53]. This ensure the relation of different types of nodes (compound, target, pathway) be fully investigated. In this study, we have developed CTPDN for comprehensive evaluating key nodes (compound, target, pathway) and edges by the shortest path via Floyd-Warshall algorithm. As our expected, key nodes of CTPDN consisting of 6 effective compounds, 6 targets, and 3 pathways. Then the joint analysis of serum metabolomics and CTPDN focused on the PI3K-AKT signaling pathway [54]. Yet vital target of this signaling pathway like PI3K was not been screened out throughout the analysis process. To elucidate the effect of targets with superior value to PI3K-AKT signaling pathway, which was not identified in CTPDN, we then established a feature-based CTI prediction model assisted by machine learning. The results of model performance (Precious = 0.96, AUC = 0.96) showed it was reliable and accurate to predict the compound-target interaction. This also supported the method of construction dataset by combining physicochemical parameters and sequence. The prediction results indicated BWG may influence the expression of target from PI3K-AKT signaling pathway. With regret that we haven’t expanded the range of applications for firming the generality of this model. Inflammatory response is a key pathogenesis of alcohol-related disease. Disorders of lipid metabolism and mocusal damage could be alleviated through PI3K-AKT signaling pathway [55, 56]. According to our findings, BWG and its effective compounds (including 2,3,4,7-tetramethoxyphenanthrene and so on) were able to activate AKT1 instead of taking PI3K as central regulator. It has been reported that AKT relating signaling is able to activate activity of STAT1 and CAT [57, 58]. This may also induce apoptosis and autophagy in tissue cells that resisting to antioxidant and inflammatory responses due to alcohol ingestion [59–61].

To verify the whole process of discovery of effective compounds and protective effective on ALD, analysis coupled with molecular docking, dynamics simulation and western blotting were proceeded. The results of molecular docking showed hydrogen bonds as well as hydrophobic bonds were the main binding modes of 6 key effective compounds and key targets. Despite of 2,3,4,7-tetramethoxyphenanthrene and astragaloside IV were failed to dock with PI3K and CAT, these varied docking energies demonstrated that the effective compounds could bind to multiple targets. Yet well-binding to all pathway-related targets would not always happen. This kind of issue just consistent with the characteristic of multi-compound and multi-target which cheer us up to study about the docking ability and the expression of key targets following. Dynamics simulation was performed on the two couples having higher binding energy. The results of stable RMSD demonstrated AKT1 was the direct target for BWG, especially 2,3,4,7-tetramethoxyphenanthrene, and AKT1 had possibility to be the direct acting target of BWG to ALD. The expression of selected targets from CTPDN were tested by western blotting. As our delightful expected, significant difference were observed which key targets, including AKT1 and pAKT1, were influenced in the expression levels after BWG intervention.

The study’s limitations can be attributed to the absence of validation methods as well as the discuss about the effect of polysaccharides. The application of surface plasmon resonance, cellular thermal shift assay and multiple spectroscopy is hypothesised to provide stronger evidence of effective compounds and targets. Proper combination these experiments with computational results is one of our future task. Additionally, the study fails to address the role of polysaccharides, despite their relatively high content in BWG and significant pharmacodynamic potential. In that case, improving the screening criteria of the compounds in order to incorporate polysaccharide into the CTPDN is the possible approach for overcoming this shortage.

## Conclusion

In this study, we demonstrated the integration network-based approach assisting by machine learning for identifying effective compounds of herbal formula. This approach reduces the workload of identifying key compounds from a complex system and focuses on the actual acting pathway as well as relating targets through multidimensional high-throughput data. Application of machine learning provide a reliable convenient workflow to understand the mechanism of herbal formula. Moreover, this study also revealed AKT1 was the notable target for BWG, and that 2,3,4,7-tetramethoxyphenanthrene was the marker compound for BWG against ALD. In conclusion, this approach provides a view of understanding the mechanism of the characteristic which are multiple compounds and multiple targets of a complex compound system.

## Supplementary Information

Additional file 1

## Data Availability

The datasets used and/or analysed during the current study are available from the corresponding author on reasonable request. The code of the model and dataset were available in github (https://github.com/Lexie0926/Code-for-CTPDN.git).

## Abbreviations

ADMET: Absorption, distribution, metabolism, excretion and toxicity
AKT1: Threonine kinase 1
ALD: Alcoholic liver disease
ALT: Alanine aminotransaminase
ANOVA: One-way analysis of variance
AS: Aqueous solubility
AST: Aspartate aminotransferase
AUC: Area under the curve
BBB: Blood–brain barrier
BCA: Bicinchoninic acid assay
BWG: Baiji Wuweizi Granules
BWG-H: High dose of BWG
BWG-L: Low dose of BWG
BWG-M: Medium dose of BWG
CAT: Catalase
Con: Control group
CTI: Compound Target interaction
CTPDN: Compound Target-pathway-disease” network
CYP2D6: Cytochrome P450 2D6 inhibition
DL: Drug-likeness value
ECFP6: Extended connectivity fingerprints 6
EF: ECFP6-target full length sequence
EM: ECFP6-target functional length sequence
FC: Fold change
GO: Gene ontology
H&E: Hematoxylin and eosin
HMDB: Human metabolome database
KEGG: Kyoto encyclopedia of genes and genomes
LSD: Least significant difference
MACCS: Molecular ACCess system
MCC: Matthews correlation coefficient
MCODE: Molecular complex detection
MDA: Malondialdehyde
MF: MACCS-target full length sequence
MM: MACCS-target functional length sequence
Mod: Model group
OB: Oral bio-availability
OMIM: Online mendelian inheritance in man
OPLS-DA: Orthogonal partial least squares discriminant analysis
PDI: Pathway-disease interaction
PI3K: Phosphatidylin-ositol-3-kinase
PLS-DA: Partial least squares discriminant analysis
Pos: Positive group
PPB: Plasma protein binding
PPI: Protein–protein interaction
PVDF: Polyvinylidene difluoride
QC: Quality control samples
RF: Random forest
RIPA: Radioimmunoprecipitation assay buffer
ROC: Receiver operating characteristic
SDS-PAGE: Sodium dodecyl sulfate–polyacrylamide gel electrophoresis
SMILES: Simplified molecular input line entry system
SOD: Superoxide dismutase
STAT1: Signal transducerand activator of transcription 1
TBST: Tris buffered saline with tween
TC: Total cholesterol
TCM: Traditional Chinese medicine
TCMID: Traditional Chinese medicine integrated database
TCMSP: Traditional Chinese medicine systems pharmacology database and analysis platform
TG: Total triglyceride
TPI: Target-pathway interaction
TTI: Target-target interaction
UPLC: Ultra performance liquid chromatography
VIP: Variable importance projection
