# Supplementary material for: Machine learning-assisted analysis of serum metabolomics and network pharmacology reveals the effective compound from herbal formula against alcoholic liver injury
Source: Chin Med. 2025 Apr 11;20:48. doi: 10.1186/s13020-025-01094-1 (PMC11992827; doi:10.1186/s13020-025-01094-1)
Supplement: Supplementary file 1 — Additional file 1 [file 13020_2025_1094_MOESM1_ESM.docx]

**Supplementary information**

**Machine learning-assisted analysis of serum metabolomics and network pharmacology reveals the effective compound from herbal formula against alcoholic liver injury**

Jiamu Ma^a^, Peng Wei^a^, Xiao Xu^b^, Ruijuan Dong^a^, Xixi Deng^a^, Feng Zhang^a^, Mengyu Sun^a^, Mingxia Li^a^, Wei Liu^a^, Jianling Yao^a^, Yu Cao^a^, Letian Ying^a^, Yuqing Yang^a^, Yongqi Yang^a^, Xiaopeng Wu^b*^, Gaimei She^a^^*^

a Beijing University of Chinese Medicine, Fangshan District, 100029 Beijing, China

b Analysis and Test Center, Chinese Academy of Tropical Agricultural Sciences, 571101 Haikou, China

^*^Corresponding author

Xiaopeng Wu, Analysis and Test Center, Chinese Academy of Tropical Agricultural Sciences, 571101 Haikou, China

Tel.: +86-898-66895968; Fax: +86-898-66895968.

E-mail address: dygxzx@126.com (X.P. Wu).

Gaimei She, School of Chinese Meteria Medica, Beijing University of Chinese Medicine, Fangshan District, 100029 Beijing, China

Tel.: +86-10-5391-2129; Fax: +86-10-5391-2129.

E-mail address: shegaimei@126.com (G.M. She).

# Contents

**1 Materials and methods**

**2 Results**

**3 Tables**

**Table S1** Verification of optimal color development conditions.

**Table S2** Results of total sugar content in three batches of BWG.

**Table S3** Results of methodological investigation of determination of total sugar.

**Table S4** Results of methodological investigation for content determination by HPLC.

**Table S5** Effects on mouse’s body weight and visceral indexes of BWG

**Table S6** Results of methodological examination of serum metabolite.

**Table S7** Identification and annotation of differential serum metabolite.

**Table S8** Biomarkers of distinguishing Con, Mod, and BWG group from serum metabolite.

**Table S9** Summary network parameters of cluster one based on MCODE.

**Table S10** Enrichment of GO and KEGG analysis for relative target of BWG.

**Table S11** Selected pathways to participate in the conduction of CTPDN.

**Table S12** ADMET prediction results and important parameters in CTPDN of candidate components.

**4 Figures**

**Fig. S1** Full-wavelength scanning image of BWG aqueous solution.

**Fig. S2** Full wavelength UV scanning diagram of phenol sulfuric acid method and anthrone sulfuric acid method.

**Fig. S3** Investigation of phenol dosage.

**Fig. S4** Investigation of sulfuric acid consumption.

**Fig. S5** Investigation of reaction temperature.

**Fig. S6** Investigation of reaction time.

**Fig. S7** Standard curve for total sugar content determination.

**Fig. S8** PCA and PLS-DA analysis for serum metabolites.

**Fig S9** Heatmap of metabolites detected under negative ion mode and positive ion mode.

**Fig. S10** Analyses for identifying the differential metabolites.

**Fig. S11** Heatmap of abundance of differential metabolites.

**Fig. S12** Prediction of functional components from BWG.

**Fig. S13** Molecular docking model of PI3K and effective compounds.

**Fig. S14** Molecular docking model of AKT1 and effective compounds.

**Fig. S15** Molecular docking model of CAT and effective compounds.

**Fig. S16** Molecular docking model of STAT1 and effective compounds.

**Fig. S17** Western blotting full scan of STAT1.

**Fig. S18** Western blotting full scan of AKT1.

**Fig. S19** Western blotting full scan of Catalase.

**Fig. S20** Western blotting full scan of β-actin.

**5 References**

## 1 Materials and methods

### 1.1 Total sugar content

According to the literature, the selection of content determination method (phenol-sulfuric acid method or anthrone-sulfuric acid method), detection wavelength, dosage of phenol and sulfuric acid, reaction temperature, and reaction time were examined during the optimization process of determination of total sugar content. Meanwhile, the standard curve and methodological contents such as linearity, precision, stability, repeatability, and sample recovery rate were also examined to establish a stable and precious content determination method for total sugar.

### 1.2 Quantification of five compounds by HPLC-DAD

The LC-20A Prominence HPLC system (Shimadzu Corp, Kyoto, Japan) was applied. Analyses were carried out on Kromasil C18 column (4.6 mm × 250 mm, 5 μm). The mobile phase was: 0.1% formic acid water (solvent A) and acetonitrile (solvent B). The gradient conditions were set as following: 0-10 min, 85% - 78% A; 10-30 min, 78% A; 30-33 min, 78%-75% A; 33-45 min, 75%-50% A; 45-50 min, 50%- 30% A; 50-60 min, 30% A; 60-65 min, 30%-10% A; 65-75 min, 10% A. The injection volume was 5.0 μL, while the flow rate was 1.0 mL/min and the column temperature was 30℃. The detection wavelength was set as 254 nm (for militarine, formononetin, nobiletin, and schisandrin) and 283 nm (for hesperidin).

### 1.3 Chromatographic and mass spectrometric conditions for matabolomic analysis

The mobile phase was 0.1% formic acid in water (A) and acetonitrile (B). The gradient elution program was set as below: 0.00~3.00 min, 5.00%~10.00% B; 3.00~5.00 min, 20.00%~70.00% B; 5.00~10.00 min, 70.00%~95.00% B; 10.00~15.00 min, 95.00% B; 15.00~17.00, 5.00% B. The column temperature was 40 ℃, the flow rate was 0.30 mL/min, and the injection volume was 5.00 μL.

The mass spectrometry conditions were performed using an electrospray ionization source (ESI) in simultaneous positive and negative ion detection modes. The sheath gas flow rate was 35 arb, the auxiliary gas flow rate was 15 arb, while the capillary temperature was 350 ℃, and the spray voltages were 3.5 kV (+) and 3.8 kV (-). The SIM and MS^2^ scans were acquired with a resolution of 17500 and the AGC target was 3 × 10^6^. The scan range of full mass was set at *m/z* 80~1050. The step-normalized collision energies were 30, 40 and 50 V.

## 2 Results

### 2.1 Establishment of method for determination of total sugar

#### 2.1.1 Identification of method and wavelength

Firstly, the sample solution was proceeded by UV full wavelength scanning. As shown in **Fig. S1**, there was no significant absorption peak in the range of 200 - 800 nm, except at 278 nm. These results indicated that the original color of the sample solution would not affect the determination of polysaccharide content. Therefore, the blank control was chosen as water.

To identify the proper measure method, the reference solution of glucose, the reagent blank solution, and the sample solution were subjected to scan full wavelength for comparing phenol-sulfuric acid method and anthrone-sulfuric acid method (**Fig. S2**). The maximum absorption wavelengths of the reagent blank, glucose reference solution, and BWG polysaccharide content determination test solution in the phenol-sulfuric acid method appear at 484.0, 486.0, and 486.0 nm, respectively. While the maximum absorbance wavelengths of the blank reagent, glucose reference solution, and BWG polysaccharide content determination test solution in the anthrone-ulfuric acid method appear at 586.0, 582.0, and 591.0 nm, respectively. It was obvious that the content determination method developed by the phenol sulfuric acid had a more stable maximum absorption wavelength, and stable absorbance values could be measured to obtain stable polysaccharide content. Therefore, the phenol-sulfuric acid method was chosen as the method for determining the polysaccharide content of BWG, and 485 nm was selected as the detection wavelength.

#### 2.1.2 Optimization of color rendering conditions

##### (1) Investigation of phenol dosage

The absorbance values of the test solution were investigated by adding different volumes of 5% phenol solution. The results, as shown in **Fig. S3**, showed that with the increase of phenol dosage, the absorbance showed an overall trend of first increasing and then decreasing. When the addition amount was 1.2 mL, the absorbance reached its maximum value (0.367 ± 0.011). Therefore, the addition amount of phenol was 1.2 mL.

##### (2) Investigation of sulfuric acid dosage

By adding different volumes of concentrated sulfuric acid to investigate the absorbance values of the test solution, as shown in **Fig. S4**, the absorbance showed a trend of first increasing and then decreasing with the increase of concentrated sulfuric acid dosage. When the dosage was 5.5 mL, the absorbance reached its maximum value (0.421 ± 0.011). Therefore, the amount of phenol added was 5.5 mL.

##### (3) Investigation of reaction temperature

In the phenol-sulfuric acid method, the reaction between sulfuric acid and phenol is exothermic, so the influence of ambient temperature on the entire system is largely dependent on maintaining the reaction temperature. Research has shown that when the ambient temperature is low, the measured absorbance fluctuates greatly. And the absorbance measurement value tends to stabilize when the temperature is above 80℃. Therefore, the range of reaction temperature investigated in this investigation was set to 70 - 100℃. The results shown in **Fig. S5** showed that as the water bath temperature increases, the absorbance first increases and then decreases. When the addition amount is 90 ℃, the absorbance reaches its maximum value (0.421 ± 0.011). Therefore, the colorimetric reaction temperature is selected as 90℃.

##### (4) Investigation of reaction time

The absorbance values of the test solution were investigated by different reaction times of the colorimetric reaction. The result was shown in Fig. S6, which showed that with the increase of reaction time, the absorbance first increases and then decreases. When the reaction time is 15 min, the absorbance reached its maximum value (0.421 ± 0.011). Therefore, the colorimetric reaction temperature was selected as 15 min.

##### (5) Verification of the besr colormetric condition

The results are shown in **Table S1**. The average absorbance of parallel samples was 0.424, with an RSD of 0.49%. The optimal color development conditions were screened and found to be feasible.

#### 2.1.3 Methodological investigation

The results of methodological investigation of content determination method for total sugar were shown in **Fig. S7** and **Table S2**. The results indicated the established content determination method was applicable to BWG.

#### 2.1.4 Content determination of total sugar

Three batches of samples were prepared in parallel (batch number: 20220312, 20220313, 20220314). 2.0 mL of the BWG test solution was accurately measured in a stoppered test tube. The absorbance was determined using the optimized method, and the total sugar content was calculated. The results were shown in **Table S2**.

### 2.1 ADMET prediction

We used computer simulations to evaluate pharmacokinetics properties of components. The results are shown in **Fig. S12**. Two important chemical descriptions (PSA_2D, AlogP98) were chosen to be coordinate axis of ADMET prediction. Four ellipses were drawn to define the component physical and chemical properties, the dots were supposed to be components in addition. The results of the ADMET properties are shown in Table 3. Those results were used for evaluate components by following radar graph estimation.

## 3 Tables

**Table S1** Verification of optimal color development conditions.

| Test | Absorbance（A） | Average absorbance（A） | RSD（%） |
| --- | --- | --- | --- |
| 1 | 0.422 | 0.424 | 0.49 |
| 2 | 0.426 |  |  |
| 3 | 0.423 |  |  |

**Table S2** Results of total sugar content in three batches of BWG.

| Batch No. | Sampling weight（g） | Absorbance（A） | Total sugar content（mg/g） | Average value（mg/g） | RSD（%） | Average（mg/g） | RSD（%） |
| --- | --- | --- | --- | --- | --- | --- | --- |
| 20220312  20220312 | 0.5002 | 0.440 | 35.1025 | 34.5194 | 2.39 | 33.7681 | 2.4 |
|  | 0.4997 | 0.425 | 33.9362 |  |  |  |  |
| 20220313 | 0.5008 | 0.410 | 32.6629 | 33.0960 | 1.85 |  |  |
| 20220313 | 0.4998 | 0.420 | 33.5291 |  |  |  |  |
| 20220314 | 0.5003 | 0.425 | 33.8955 | 33.6889 | 0.87 |  |  |
| 20220314 | 0.5005 | 0.420 | 33.4822 |  |  |  |  |

**Table S3** Results of methodological investigation of determination of total sugar.

| Investigation items | Value |
| --- | --- |
| Standard curve | *Y* = 13.0132 *X*+0.0013 (R^2^=0.9994) |
| Linear range | 0.010~0.100 mg/mL |
| Precision (RSD %) | 1.40 |
| Repeatability (RSD %) | 1.10 |
| Stability in 90 min (RSD %) | 1.10 |
| Sample recovery rate (%) | 99.00 |
| Sample recovery rate (RSD %) | 2.50 |

**Table S4** Results of methodological investigation for content determination by HPLC.

| Compound | Precision (%) | Repeatability (%) | Stability in 24 h (%) | Regression equation | Range (mg/mL) | *R*^2^ | Average recovery (%) |
| --- | --- | --- | --- | --- | --- | --- | --- |
| Hesperidin | 1.59 | 2.72 | 1.50 | Y = 1016900 X - 24350.7 | 0.0100 ~ 0.1000 | 0.9999 | 103.97 |
| Militarine | 0.79 | 2.74 | 1.98 | Y = 315660 X – 2149.3 | 0.0936 ~ 0.9360 | 0.9993 | 100.57 |
| Formononetin | 2.50 | 2.86 | 1.16 | Y = 2386800 X + 651.01 | 0.0034 ~ 0.0336 | 0.9997 | 99.18 |
| Nobiletin | 0.48 | 2.80 | 2.75 | Y = 1326000 X + 567.77 | 0.0032 ~ 0.0320 | 0.9998 | 97.36 |
| Schisandrin | 0.48 | 1.27 | 0.82 | Y = 13016700 X + 16044.5 | 0.0188 ~0.1880 | 0.9998 | 100.88 |

**Table S5** Effects on mouse’s body weight and visceral indexes of BWG.

| Group | Initial weight (g) | Final weight (g) | Organ index (%) | | | | | |
| --- | --- | --- | --- | --- | --- | --- | --- | --- |
|  |  |  | Liver | Heart | Spleen | Kidney | Thymus | Stomach |
| Con | 21.32 ± 1.71 | 23.08 ± 1.41 | 3.49 ± 0.21 | 0.57 ± 0.03 | 0.18 ± 0.02 | 1.21 ± 0.07 | 0.05 ± 0.01 | 0.29 ± 0.04 |
| Mod | 20.37 ± 0.82 | 22.69 ± 1.18 | 3.81 ± 0.11^*^ | 0.51 ± 0.03^*^ | 0.18 ± 0.06 | 1.26 ± 0.08 | 0.06 ± 0.02 | 0.30 ± 0.08 |
| Pos | 20.29 ± 0.61 | 23.07 ± 2.14 | 3.88 ± 0.48^*^ | 0.43 ± 0.08^**##^ | 0.24 ± 0.06 | 1.28 ± 0.26^**##^ | 0.15 ± 0.07^**##^ | 0.26 ± 0.04 |
| BWG-L | 20.51 ± 0.40 | 24.47 ± 1.29 | 3.89 ± 0.61^*^ | 0.50 ± 0.02^**##^ | 0.19 ± 0.01 | 1.22 ± 0.05 | 0.04 ± 0.02 | 0.16 ± 0.14^* ##^ |
| BWG-M | 20.64 ± 0.74 | 23.70 ± 1.62 | 3.83 ± 0.44^*^ | 0.55 ± 0.02^*^ | 0.17 ± 0.02 | 1.29 ± 0.06 | 0.07 ± 0.02 | 0.20 ± 0.15^* ##^ |
| BWG-H | 20.64 ± 0.76 | 23.50 ± 0.76 | 4.16 ± 0.10^*^ | 0.48 ± 0.04^**##^ | 0.17 ± 0.03 | 1.19 ± 0.05^* #^ | 0.06 ± 0.02 | 0.35 ± 0.02 |

^*^*P* <0.05, ^**^ *P* <0.01, significantly different from Con group (Pair-fed)；^#^ *P* < 0.05, ^##^ *P* < 0.01, significantly different from Mod group.

**Table S6** Results of methodological examination of serum metabolite.

| Methodological examination | Percentage of characteristic peaks with peak area (RSD < 30%) (%) | Correlation coefficient between samples |
| --- | --- | --- |
| Precision | 81.78 | 0.92~1.00 |
| Repeatability | 80.73 | 0.98~1.00 |
| Stability | 80.48 | 0.84~1.00 |

**Table S7** Identification and annotation of differential serum metabolite.

| Metabolite | Retention time (min) | *m*/*z* | Molecular formula | Ion mode | HMDB ID | Changing trend | |
| --- | --- | --- | --- | --- | --- | --- | --- |
|  |  |  |  |  |  | Mod vs Con | Mod vs BSW |
| 1-Oleoylglycerophosphoserine | 6.298 | 563.2006 | C_24_H_44_NO_9_P | [M-H]^-^ | HMDB0061694 | ↓ | ↓ |
| 6-Hydroxypentadecanedioic acid | 14.475 | 299.7306 | C_15_H_28_O_5_ | [M-H]^-^ | HMDB0031885 | ↓ | ↓ |
| Melleolide H | 6.397 | 456.2438 | C_24_H_30_O_7_ | [M-H]^-^ | HMDB0038788 | ↑ | ↓ |
| Stearic acid | 14.696 | 295.7352 | C_18_H_36_O_2_ | [M-H]^-^ | HMDB0000827 | ↓ | ↓ |
| Corchorifatty acid F | 7.536 | 344.1528 | C_18_H_32_O_5_ | [M-H]^-^ | HMDB0035919 | ↑ | ↓ |
| (-)-Pyrifolidine | 8.688 | 406.2228 | C_20_H_36_N_2_O_3_S | [M-H]^-^ | HMDB0033523 | ↓ | ↑ |
| Glyinflanin H | 10.007 | 415.2248 | C_19_H_16_O_4_ | [M-H]^-^ | HMDB0041303 | ↑ | ↑ |
| Heptadecanoic acid | 13.611 | 280.9399 | C_17_H_34_O_2_ | [M-H]^-^ | HMDB0002259 | ↑ | ↑ |
| Methyl (9Z)-6'-oxo-6,5'-diapo-6-carotenoate | 8.063 | 415.2256 | C_19_H_37_O_6_P | [M-H]^-^ | HMDB0031977 | ↑ | ↑ |
| 3'-Deaminofusarochromanone | 6.218 | 288.0591 | C_15_H_19_NO_4_ | [M-H]^-^ | HMDB0041328 | ↓ | ↑ |
| Porson | 5.808 | 408.234 | C_22_H_26_O_6_ | [M-H]^-^ | HMDB0030810 | ↓ | ↑ |
| Bopindolol | 0.957 | 401.9767 | C_23_H_28_N_2_O_3_ | [M-H]^-^ | HMDB0015696 | ↓ | ↓ |
| Pyridoxal 5'-phosphate | 7.501 | 255.8218 | C_8_H_10_NO_6_P | [M-H]^-^ | HMDB0001491 | ↓ | ↓ |
| 19,20-DiHDPA | 1.361 | 381.0303 | C_22_H_34_O_4_ | [M-H]^-^ | HMDB0010214 | ↑ | ↑ |
| 2-Dodecylbenzenesulfonic acid | 1.897 | 342.1189 | C_21_H_26_O_3_ | [M-H]^-^ | HMDB0031031 | ↓ | ↓ |
| Heptaethylene glycol | 0.943 | 342.1186 | C_18_H_30_O_3_S | [M-H]^-^ | HMDB0061835 | ↓ | ↓ |
| 17alpha-Estradiol | 7.519 | 283.1013 | C_18_H_24_O_2_ | [M-H]^-^ | HMDB0000429 | ↓ | ↓ |
| Desacetyl-nitazoxanide | 13.441 | 274.7884 | C_10_H_7_N_3_O_4_S | [M-H]^-^ | HMDB0060597 | ↑ | ↓ |
| Glycocholic acid | 7.164 | 498.2886 | C_26_H_43_NO_6_ | [M-H]^-^ | HMDB0000138 | ↓ | ↓ |
| Monascoflavin | 2.241 | 376.1007 | C_21_H_26_O_5_ | [M-H]^-^ | HMDB0030792 | ↓ | ↓ |
| 3-Oxohexadecanoic acid | 3.56 | 280.8662 | C_16_H_30_O_3_ | [M-H]^-^ | HMDB0010733 | ↓ | ↑ |
| Deoxyinosine | 0.612 | 259.9334 | C_10_H_12_N_4_O_4_ | [M-H]^-^ | HMDB0000071 | ↓ | ↑ |
| Irilone | 7.374 | 311.1151 | C_16_H_10_O_6_ | [M-H]^-^ | HMDB0033820 | ↓ | ↑ |
| Dihydrocortisol | 8.578 | 383.2229 | C_21_H_32_O_5_ | [M-H]^-^ | HMDB0003259 | ↓ | ↑ |
| 4-Dodecylbenzenesulfonic Acid | 5.651 | 341.9601 | C_18_H_30_O_3_S | [M-H]^-^ | HMDB0059915 | ↑ | ↑ |
| 4-[(2,4-Dihydroxyphenyl)azo]benzenesulfonic acid | 13.916 | 305.9271 | C_12_H_10_N_2_O_5_S | [M-H]^-^ | HMDB0037835 | ↑ | ↑ |
| Acitretin | 1.897 | 342.1189 | C_21_H_26_O_3_ | [M-H]^-^ | HMDB0014602 | ↑ | ↓ |
| (10Z,14E,16E)-10,14,16-Octadecatrien-12-ynoic acid | 7.259 | 285.1532 | C_18_H_26_O_2_ | [M-H]^-^ | HMDB0035963 | ↓ | ↑ |
| 7Z,10Z-Hexadecadienoic acid | 6.624 | 260.1324 | C_16_H_28_O_2_ | [M-H]^-^ | HMDB0000477 | ↓ | ↓ |
| Sulfamethizole | 13.424 | 279.9204 | C_9_H_10_N_4_O_2_S_2_ | [M-H]^-^ | HMDB0014715 | ↑ | ↑ |
| Atenolol | 0.925 | 276.1084 | C_14_H_22_N_2_O_3_ | [M-H]^-^ | HMDB0001924 | ↓ | ↓ |
| Fenamiphos | 7.511 | 317.2121 | C_13_H_22_NO_3_PS | [M-H]^-^ | HMDB0031787 | ↑ | ↑ |
| 1-(3-Furanyl)-6,7-dihydroxy-4,8-dimethyl-1-nonanone | 4.249 | 278.1144 | C_15_H_24_O_4_ | [M-H]^-^ | HMDB0038153 | ↓ | ↓ |
| (6beta,8betaOH)-6,8-Dihydroxy-7(11)-eremophilen-12,8-olide | 1.515 | 275.9966 | C_15_H_22_O_4_ | [M-H]^-^ | HMDB0035644 | ↑ | ↑ |
| Acetyl tributyl citrate | 8.244 | 426.1888 | C_20_H_34_O_8_ | [M-H]^-^ | HMDB0034159 | ↓ | ↓ |
| 3-Hydroxytetradecanedioic acid | 5.754 | 285.134 | C_14_H_26_O_5_ | [M-H]^-^ | HMDB0000394 | ↑ | ↑ |
| (1(10)E,4E,6a,9b)-9-(3-Methylbutanoyloxy)-1(10),4,11(13)-germacratrien-12,6-olide | 16.511 | 348.8122 | C_20_H_28_O_4_ | [M-H]^-^ | HMDB0031375 | ↑ | ↑ |
| Cyclandelate | 5.701 | 287.1615 | C_17_H_24_O_3_ | [M-H]^-^ | HMDB0015586 | ↑ | ↑ |
| Blennin D | 1.639 | 276.0171 | C_15_H_22_O_4_ | [M-H]^-^ | HMDB0031902 | ↓ | ↓ |
| Oxybuprocaine | 2.094 | 323.071 | C_17_H_28_N_2_O_3_ | [M-H]^-^ | HMDB0015029 | ↓ | ↓ |
| Panaquinquecol 2 | 7.565 | 287.169 | C_17_H_24_O_3_ | [M-H]^-^ | HMDB0038938 | ↓ | ↑ |
| C.I. Natural Red 20 | 0.777 | 299.169 | C_16_H_16_O_5_ | [M-H]^-^ | HMDB0030579 | ↓ | ↑ |
| Valproic acid glucuronide | 6.476 | 335.1505 | C_14_H_24_O_8_ | [M-H]^-^ | HMDB0000901 | ↓ | ↑ |
| 14alpha-Hydroxypaxilline | 12.332 | 481.2363 | C_27_H_33_NO_5_ | [M-H]^-^ | HMDB0040978 | ↓ | ↓ |
| Sotalol | 6.945 | 283.0413 | C_12_H_20_N_2_O_3_S | [M-H]^-^ | HMDB0014632 | ↑ | ↑ |
| Gingerol | 8.742 | 306.1087 | C_17_H_26_O_4_ | [M-H]^-^ | HMDB0005783 | ↓ | ↑ |
| (gamma-Glutamyl-gamma-glutamyl)-S-methylcysteine | 7.441 | 416.2126 | C_14_H_23_N_3_O_8_S | [M-H]^-^ | HMDB0039424 | ↑ | ↑ |
| Domesticoside | 8.299 | 361.2376 | C_15_H_20_O_9_ | [M-H]^-^ | HMDB0029647 | ↓ | ↑ |
| 12S-HHT | 6.561 | 291.1604 | C_17_H_28_O_3_ | [M-H]^-^ | HMDB0012535 | ↓ | ↓ |
| 3-Hydroxyhexadecanoic acid | 10.746 | 283.1374 | C_16_H_32_O_3_ | [M-H]^-^ | HMDB0061658 | ↑ | ↑ |
| Ascorbic acid 2-sulfate | 11.055 | 264.0879 | C_6_H_8_O_9_S | [M-H]^-^ | HMDB0060649 | ↑ | ↑ |
| Linolenelaidic acid | 5.815 | 289.1297 | C_18_H_30_O_2_ | [M-H]^-^ | HMDB0030964 | ↑ | ↑ |
| Bisacurone epoxide | 0.935 | 278.1243 | C_15_H_24_O_4_ | [M-H]^-^ | HMDB0038503 | ↓ | ↓ |
| (9S,10S)-9,10-dihydroxyoctadecanoate | 8.55 | 331.2279 | C_18_H_36_O_4_ | [M-H]^-^ | HMDB0059633 | ↑ | ↑ |
| 12 Hydroxy arachidonic acid | 7.5 | 335.2227 | C_20_H_32_O_3_ | [M-H]^-^ | HMDB0060101 | ↑ | ↑ |
| 11-Hydroxyhexadecanoic acid | 10.896 | 283.141 | C_16_H_32_O_3_ | [M-H]^-^ | HMDB0112189 | ↑ | ↑ |
| Asparaginylarginine | 13.36 | 299.2593 | C_10_H_20_N_6_O_4_ | [M-H]^-^ | HMDB0028725 | ↓ | ↓ |
| Panaquinquecol 7 | 6.749 | 287.15 | C_17_H_24_O_3_ | [M-H]^-^ | HMDB0041201 | ↓ | ↑ |
| 16a-Hydroxyestrone | 10.793 | 297.1536 | C_18_H_22_O_3_ | [M-H]^-^ | HMDB0000335 | ↓ | ↑ |
| gamma-L-Glutamyl-S-(2-carboxy-1-propyl)cysteinylglycine | 7.441 | 416.2126 | C_14_H_23_N_3_O_8_S | [M-H]^-^ | HMDB0029394 | ↑ | ↑ |
| gamma-L-Glutamyl-L-methionine sulfoxide | 6.536 | 306.0208 | C_10_H_18_N_2_O_6_S | [M-H]^-^ | HMDB0038613 | ↓ | ↓ |
| 4',7-Di-O-methylcatechin | 6.889 | 333.1374 | C_17_H_18_O_6_ | [M-H]^-^ | HMDB0030663 | ↑ | ↑ |
| Nb-Feruloyltryptamine | 0.885 | 352.1214 | C_20_H_20_N_2_O_3_ | [M-H]^-^ | HMDB0041519 | ↓ | ↑ |
| [6]-Dehydrogingerdione | 3.351 | 302.0557 | C_17_H_22_O_4_ | [M-H]^-^ | HMDB0029474 | ↓ | ↑ |
| gamma-Glutamylvaline | 0.803 | 254.9166 | C_10_H_18_N_2_O_5_ | [M-H]^-^ | HMDB0011172 | ↓ | ↑ |
| (E,E)-Trichostachine | 13.116 | 281.248 | C_16_H_17_NO_3_ | [M-H]^-^ | HMDB0029374 | ↑ | ↑ |
| 15-Epi-lipoxin B5 | 5.833 | 367.1035 | C_20_H_30_O_5_ | [M-H]^-^ | HMDB0012588 | ↓ | ↓ |
| Nandrolone | 7.259 | 285.1532 | C_18_H_26_O_2_ | [M-H]^-^ | HMDB0002725 | ↓ | ↑ |
| 2-Phenylethanol glucuronide | 9.918 | 311.2225 | C_14_H_18_O_7_ | [M-H]^-^ | HMDB0010350 | ↓ | ↓ |
| 10-Hydroxymelleolide | 6.564 | 417.1907 | C_23_H_28_O_7_ | [M+H]^+^ | HMDB0038786 | ↑ | ↑ |
| Leukotriene E3 | 6.782 | 442.2632 | C_23_H_39_NO_5_S | [M+H]^+^ | HMDB0002355 | ↓ | ↓ |
| Theogallin | 6.829 | 345.0866 | C_14_H_16_O_10_ | [M+H]^+^ | HMDB0039287 | ↓ | ↓ |
| 11S,15S-dihydroxy-14R-(S-glutathionyl)-5Z,8Z,12E-eicosatrienoic acid | 6.124 | 644.3235 | C_30_H_49_N_3_O_10_S | [M+H]^+^ | HMDB0062478 | ↓ | ↓ |
| 10,11-Dihydro-12R-hydroxy-leukotriene E4 | 6.167 | 458.2566 | C_23_H_39_NO_6_S | [M+H]^+^ | HMDB0012501 | ↓ | ↓ |
| Neoacrimarine E | 5.664 | 614.2476 | C_35_H_35_NO_9_ | [M+H]^+^ | HMDB0040767 | ↑ | ↑ |
| Flavoxate | 6.327 | 392.176 | C_24_H_25_NO_4_ | [M+H]^+^ | HMDB0015279 | ↓ | ↓ |
| Enalaprilat | 6.366 | 349.1765 | C_18_H_24_N_2_O_5_ | [M+H]^+^ | HMDB0041886 | ↓ | ↓ |
| Dabigatran etexilate | 6.627 | 628.3261 | C_34_H_41_N_7_O_5_ | [M+H]^+^ | HMDB0015641 | ↓ | ↓ |
| Pipernonaline | 4.82 | 342.2141 | C_21_H_27_NO_3_ | [M+H]^+^ | HMDB0030339 | ↓ | ↓ |
| Semilicoisoflavone B | 6.178 | 353.1058 | C_20_H_16_O_6_ | [M+H]^+^ | HMDB0035184 | ↓ | ↓ |
| Tryptophyl-Glutamine | 5.848 | 333.1561 | C_16_H_20_N_4_O_4_ | [M+H]^+^ | HMDB0029081 | ↓ | ↓ |
| Mandelonitrile rutinoside | 5.903 | 442.1638 | C_20_H_27_NO_10_ | [M+H]^+^ | HMDB0032803 | ↓ | ↑ |
| Cycloartanyl ferulate | 8.805 | 605.4557 | C_40_H_60_O_4_ | [M+H]^+^ | HMDB0036295 | ↓ | ↓ |
| Imidaprilat | 6.288 | 378.1615 | C_18_H_23_N_3_O_6_ | [M+H]^+^ | HMDB0041908 | ↓ | ↓ |
| Curcumin II | 1.888 | 367.15 | C_22_H_22_O_5_ | [M+H]^+^ | HMDB0039610 | ↑ | ↑ |
| Glyceollin I | 0.789 | 339.1166 | C_20_H_18_O_5_ | [M+H]^+^ | HMDB0033901 | ↑ | ↑ |
| 3'-N'-Acetylfusarochromanone | 6.295 | 335.1603 | C_17_H_22_N_2_O_5_ | [M+H]^+^ | HMDB0037499 | ↓ | ↓ |
| 4-(2-Nitroethyl)phenyl primeveroside | 5.858 | 462.1554 | C_19_H_27_NO_12_ | [M+H]^+^ | HMDB0031742 | ↓ | ↓ |
| Lafutidine | 8.603 | 432.1913 | C_22_H_29_N_3_O_4_S | [M+H]^+^ | HMDB0240216 | ↑ | ↑ |
| Aflatoxin B1 dialcohol | 0.889 | 331.1109 | C_18_H_18_O_6_ | [M+H]^+^ | HMDB0011672 | ↑ | ↑ |
| Biocytin | 5.919 | 373.1884 | C_16_H_28_N_4_O_4_S | [M+H]^+^ | HMDB0003134 | ↓ | ↓ |
| Prostaglandin A1 | 7.114 | 337.2374 | C_20_H_32_O_4_ | [M+H]^+^ | HMDB0002656 | ↓ | ↓ |
| Avenestergenin A1 | 9.755 | 638.4026 | C_38_H_55_NO_7_ | [M+H]^+^ | HMDB0035264 | ↓ | ↑ |
| Leukotriene E4 | 6.14 | 440.2409 | C_23_H_37_NO_5_S | [M+H]^+^ | HMDB0002200 | ↑ | ↑ |
| Nb-trans-p-Coumaroylserotonin glucoside | 6.183 | 485.1913 | C_25_H_28_N_2_O_8_ | [M+H]^+^ | HMDB0032760 | ↓ | ↓ |
| Retrofractamide D | 5.567 | 342.213 | C_21_H_27_NO_3_ | [M+H]^+^ | HMDB0033450 | ↓ | ↓ |
| Cotinine glucuronide | 0.789 | 353.1301 | C_16_H_20_N_2_O_7_ | [M+H]^+^ | HMDB0001013 | ↑ | ↑ |
| Oxomemazine | 0.782 | 331.1471 | C_18_H_22_N_2_O_2_S | [M+H]^+^ | HMDB0240230 | ↑ | ↑ |
| Mukurozidiol | 1.617 | 335.1187 | C_17_H_18_O_7_ | [M+H]^+^ | HMDB0030651 | ↓ | ↓ |
| Sulfinpyrazone sulfone | 0.791 | 421.1179 | C_23_H_20_N_2_O_4_S | [M+H]^+^ | HMDB0060944 | ↑ | ↑ |
| Nicotine glucuronide | 5.761 | 339.1548 | C_16_H_22_N_2_O_6_ | [M+H]^+^ | HMDB0001272 | ↓ | ↓ |
| Nevskin | 6.907 | 401.2295 | C_24_H_32_O_5_ | [M+H]^+^ | HMDB0030162 | ↓ | ↓ |
| Glisoxepide | 5.792 | 450.1875 | C_20_H_27_N_5_O_5_S | [M+H]^+^ | HMDB0015406 | ↑ | ↑ |
| Carphenazine | 5.873 | 412.209 | C_23_H_29_N_3_O_2_S | [M+H]^+^ | HMDB0015172 | ↑ | ↑ |
| 11H-14,15-EETA | 6.895 | 337.2373 | C_20_H_32_O_4_ | [M+H]^+^ | HMDB0004693 | ↓ | ↓ |
| N2-Fructopyranosylarginine | 5.945 | 337.176 | C_12_H_24_N_4_O_7_ | [M+H]^+^ | HMDB0041541 | ↓ | ↓ |
| Garcimangosone C | 6.456 | 413.1614 | C_23_H_24_O_7_ | [M+H]^+^ | HMDB0036984 | ↓ | ↓ |
| Ethyl icosapentate | 9.506 | 331.2626 | C_22_H_34_O_2_ | [M+H]^+^ | HMDB0039530 | ↓ | ↓ |
| Sphinganine 1-phosphate | 7.206 | 382.271 | C_18_H_40_NO_5_P | [M+H]^+^ | HMDB0001383 | ↓ | ↓ |
| Tolmetin glucuronide | 7.399 | 434.1515 | C_21_H_23_NO_9_ | [M+H]^+^ | HMDB0042044 | ↓ | ↓ |
| Mollicellin A | 6.287 | 383.1162 | C_21_H_18_O_7_ | [M+H]^+^ | HMDB0033339 | ↓ | ↓ |
| Garcinone C | 8.438 | 415.1763 | C_23_H_26_O_7_ | [M+H]^+^ | HMDB0029511 | ↓ | ↓ |
| Ivacaftor | 6.61 | 393.2243 | C_24_H_28_N_2_O_3_ | [M+H]^+^ | HMDB0015705 | ↓ | ↓ |
| 2-Hydroxymyristoylcarnitine | 6.773 | 388.305 | C_21_H_41_NO_5_ | [M+H]^+^ | HMDB0013166 | ↓ | ↓ |
| Glycitin | 5.914 | 447.1281 | C_22_H_22_O_10_ | [M+H]^+^ | HMDB0002219 | ↓ | ↓ |
| Melleolide G | 7.434 | 433.22 | C_24_H_32_O_7_ | [M+H]^+^ | HMDB0038793 | ↓ | ↓ |
| Isoyatein | 8.692 | 401.1609 | C_22_H_24_O_7_ | [M+H]^+^ | HMDB0033258 | ↓ | ↓ |
| Mollicellin C | 6.378 | 413.127 | C_22_H_20_O_8_ | [M+H]^+^ | HMDB0033341 | ↓ | ↓ |
| 3-hydroxydecanoyl carnitine | 6.251 | 332.2441 | C_17_H_33_NO_5_ | [M+H]^+^ | HMDB0061636 | ↓ | ↓ |
| 3-Hydroxy-cis-5-tetradecenoylcarnitine | 6.673 | 386.2902 | C_21_H_39_NO_5_ | [M+H]^+^ | HMDB0013330 | ↓ | ↓ |
| Rubroskyrin | 1.6 | 575.1269 | C_30_H_22_O_12_ | [M+H]^+^ | HMDB0030865 | ↓ | ↑ |
| Nicardipine | 7.424 | 480.2122 | C_26_H_29_N_3_O_6_ | [M+H]^+^ | HMDB0014760 | ↑ | ↑ |
| Chlorogenoquinone | 1.627 | 353.0925 | C_16_H_16_O_9_ | [M+H]^+^ | HMDB0029383 | ↓ | ↓ |
| 3-Hydroxy-9-hexadecenoylcarnitine | 6.91 | 414.3206 | C_23_H_43_NO_5_ | [M+H]^+^ | HMDB0013333 | ↓ | ↓ |
| Pipotiazine | 6.089 | 476.2121 | C_24_H_33_N_3_O_3_S_2_ | [M+H]^+^ | HMDB0015558 | ↓ | ↓ |
| 7alpha-hydroxy-3-oxochol-4-en-24-oic Acid | 7.313 | 389.2682 | C_24_H_36_O_4_ | [M+H]^+^ | HMDB0062744 | ↓ | ↓ |
| Indacaterol | 6.532 | 393.2244 | C_24_H_28_N_2_O_3_ | [M+H]^+^ | HMDB0015608 | ↑ | ↑ |
| Urobilinogen | 6.131 | 591.3168 | C_33_H_42_N_4_O_6_ | [M+H]^+^ | HMDB0004158 | ↑ | ↑ |
| 2-Hydroxyhexadecanoylcarnitine | 7.131 | 416.3374 | C_25_H_47_NO_4_ | [M+H]^+^ | HMDB0013337 | ↓ | ↓ |
| Semilepidinoside A | 4.367 | 337.1398 | C_16_H_20_N_2_O_6_ | [M+H]^+^ | HMDB0033107 | ↓ | ↓ |
| Buspirone | 6.017 | 611.1978 | C_21_H_31_N_5_O_2_ | [M+H]^+^ | HMDB0014633 | ↓ | ↓ |
| (4E,15Z)-Bilirubin | 6.473 | 585.2714 | C_33_H_36_N_4_O_6_ | [M+H]^+^ | HMDB0000488 | ↓ | ↓ |
| Tetracosahexaenoic acid, n-3 | 7.137 | 357.279 | C_24_H_36_O_2_ | [M+H]^+^ | HMDB0060117 | ↑ | ↓ |
| Calcitroic acid | 13.642 | 375.2601 | C_23_H_34_O_4_ | [M+H]^+^ | HMDB0006472 | ↓ | ↓ |
| Tangeritin | 6.995 | 373.1284 | C_20_H_20_O_7_ | [M+H]^+^ | HMDB0030539 | ↓ | ↓ |
| N-Acetyl-7-O-acetylneuraminic acid | 6.616 | 352.1186 | C_13_H_21_NO_10_ | [M+H]^+^ | HMDB0000785 | ↓ | ↓ |
| 3,3',4',5,6,8-Hexamethoxyflavone | 7.224 | 403.138 | C_21_H_22_O_8_ | [M+H]^+^ | HMDB0035415 | ↓ | ↓ |
| 3-Hydroxyhexadecadienoylcarnitine | 6.742 | 412.3061 | C_23_H_41_NO_5_ | [M+H]^+^ | HMDB0013335 | ↓ | ↓ |
| 3-Hydroxy-11Z-octadecenoylcarnitine | 7.28 | 442.3532 | C_25_H_47_NO_5_ | [M+H]^+^ | HMDB0013339 | ↓ | ↓ |
| 7-beta-D-Glucopyranosyloxybutylidenephthalide | 6.241 | 367.1327 | C_18_H_22_O_8_ | [M+H]^+^ | HMDB0034752 | ↑ | ↓ |
| Validamycin B | 6.239 | 514.2188 | C_20_H_35_NO_14_ | [M+H]^+^ | HMDB0036593 | ↑ | ↑ |
| trans-Zeatin-O-glucoside riboside | 8.807 | 418.1884 | C_21_H_31_N_5_O_10_ | [M+H]^+^ | HMDB0032880 | ↓ | ↓ |
| 1,1'-[1,12-Dodecanediylbis(oxy)]bisbenzene | 7.076 | 355.2628 | C_24_H_34_O_2_ | [M+H]^+^ | HMDB0039760 | ↑ | ↓ |
| (E)-Casimiroedine | 2.136 | 418.1932 | C_21_H_27_N_3_O_6_ | [M+H]^+^ | HMDB0030274 | ↓ | ↓ |
| (-)-Aspidospermine | 6.672 | 355.2446 | C_22_H_30_N_2_O_2_ | [M+H]^+^ | HMDB0030361 | ↑ | ↓ |
| 18-carboxy dinor Leukotriene B4 | 6.178 | 339.1783 | C_18_H_26_O_6_ | [M+H]^+^ | HMDB0062301 | ↓ | ↓ |
| Prostaglandin B2 | 6.531 | 335.2216 | C_20_H_30_O_4_ | [M+H]^+^ | HMDB0004236 | ↓ | ↓ |
| Nabilone | 6.669 | 373.2736 | C_24_H_36_O_3_ | [M+H]^+^ | HMDB0014629 | ↑ | ↓ |

**Table S8** Biomarkers of distinguishing Con, Mod, and BWG group from serum metabolite.

| Metabolite | HMDB ID | Mod vs Con | | Mod vs BWG | |
| --- | --- | --- | --- | --- | --- |
|  |  | Rank Frequency | Importance (×10^-5^) | Rank Frequency. | Importance (×10^-5^) |
| 2-Phenylethanol glucuronide | HMDB0010350 | 0.68 | 24.3147 | 0.04 | 4.16 |
| Acetyl tributyl citrate | HMDB0034159 | 0.34 | 18.3768 | 0.46 | 19.8856 |
| Glyinflanin H | HMDB0041303 | 0.34 | 18.0451 | 0.26 | 13.8801 |
| Calcitroic acid | HMDB0006472 | 0.26 | 16.5886 | 0.64 | 28.8236 |
| Ivacaftor | HMDB0015705 | 0.24 | 16.3154 | 0.1 | 14.4428 |
| 4-Dodecylbenzenesulfonic Acid | HMDB0059915 | 0.24 | 15.1849 | 0.12 | 13.9179 |
| Melleolide G | HMDB0038793 | 0.24 | 14.2169 | 0.02 | 3.61 |

**Table S9** Summary network parameters of cluster one based on MCODE

| Target Name | Degree Layout | MCODE Score | MCODE Node Status | Clustering Coefficient | Closeness Centrality | Degree | Betweenness Centrality |
| --- | --- | --- | --- | --- | --- | --- | --- |
| AKT1 | 69 | 16.7532 | Clustered | 0.8188 | 1.0000 | 24 | 0.0150 |
| AR | 31 | 15.0000 | Clustered | 1.0000 | 0.6857 | 13 | 0.0000 |
| CASP3 | 53 | 16.7532 | Clustered | 0.8188 | 1.0000 | 24 | 0.0150 |
| CASP9 | 29 | 14.1316 | Clustered | 0.9265 | 0.7742 | 17 | 0.0025 |
| CAT | 45 | 14.9053 | Clustered | 0.8667 | 0.8889 | 21 | 0.0072 |
| CREB1 | 45 | 15.2138 | Clustered | 0.8442 | 0.9231 | 22 | 0.0105 |
| EGFR | 47 | 15.2421 | Clustered | 0.8524 | 0.8889 | 21 | 0.0090 |
| ESR1 | 49 | 13.7857 | Clustered | 0.9265 | 0.7742 | 17 | 0.0027 |
| HMOX1 | 40 | 15.0737 | Clustered | 0.9240 | 0.8276 | 19 | 0.0030 |
| ClICAM1 | 31 | 14.7810 | Clustered | 0.9346 | 0.8000 | 18 | 0.0022 |
| IKBKB | 22 | 14.1228 | Clustered | 0.9265 | 0.7742 | 17 | 0.0023 |
| IL4 | 29 | 15.9476 | Clustered | 0.9053 | 0.8571 | 20 | 0.0044 |
| JUN | 54 | 16.7532 | Clustered | 0.8188 | 1.0000 | 24 | 0.0150 |
| MAPK1 | 36 | 14.6147 | Clustered | 0.9085 | 0.8000 | 18 | 0.0038 |
| MAPK14 | 34 | 15.2138 | Clustered | 0.8261 | 0.9600 | 23 | 0.0132 |
| MAPK3 | 48 | 15.2138 | Clustered | 0.8300 | 0.9600 | 23 | 0.0127 |
| MAPK8 | 34 | 15.4545 | Clustered | 0.8442 | 0.9231 | 22 | 0.0105 |
| MMP9 | 47 | 16.7532 | Seed | 0.8188 | 1.0000 | 24 | 0.0150 |
| NOS2 | 27 | 15.0737 | Clustered | 0.9559 | 0.7742 | 17 | 0.0012 |
| PGR | 28 | 14.8897 | Clustered | 0.9890 | 0.7059 | 14 | 0.0003 |
| PPARA | 44 | 15.7857 | Clustered | 0.9064 | 0.8276 | 19 | 0.0039 |
| PPARG | 52 | 15.6017 | Clustered | 0.8947 | 0.8571 | 20 | 0.0049 |
| PTGS2 | 52 | 16.7532 | Clustered | 0.8188 | 1.0000 | 24 | 0.0150 |
| STAT1 | 30 | 15.8667 | Clustered | 0.8398 | 0.9231 | 22 | 0.0104 |
| VCAM1 | 28 | 14.9895 | Clustered | 0.9485 | 0.7742 | 17 | 0.0015 |

**Table S10** Enrichment of GO and KEGG analysis for relative target of BWG.

| Source | ID | Description | p value | q value | Gene ID | Count |
| --- | --- | --- | --- | --- | --- | --- |
| BP | GO:0009410 | response to xenobiotic stimulus | 1.78×10^-27^ | 3.22×10^-24^ | GSTP1, EGFR, CREB1, CASP3, PTGS2, STAT1, CAT, ABCC1, SLC6A4, BCL2, AHR, CYP1A2, JUN, HMOX1, GSTM1, CYP1B1, BAD, CDK1, CYP1A1, ADRA1A, UGT1A1, AKR1C1, SREBF1, GSTM2, DRD1, KCNH2, ICAM1, NOS2, SOD1, MAOB, RELA, NR1I2, CYP3A4, SLC6A3 | 34 |
|  | GO:0010038 | response to metal ion | 8.26×10^-23^ | 7.47×10^-20^ | EGFR, CREB1, CASP3, PTGS2, MAPK1, MAPK8, CAT, CA2, BCL2, CYP1A2, AKT1, JUN, HMOX1, BAD, MMP9, CDK1, CYP1A1, NCF1, CASP9, ICAM1, MAPK3, SOD1, MAOB, AKR1C3, PPP3CA, CALM1, SLC6A3, VCAM1 | 28 |
|  | GO:0031667 | response to nutrient levels | 2.74×10^-22^ | 1.66×10^-19^ | GSTP1, LDLR, EGFR, PTGS2, STAT1, MAPK1, MAPK8, CAT, SLC6A4, PPARG, BCL2, F7, AKT1, JUN, HMGCR, HMOX1, CYP1A1, UGT1A1, SREBF1, ADRB2, ICAM1, MAPK3, OPRM1, PPARD, SOD1, RELA, AKR1C3, ADRB1, PPARA, VCAM1 | 30 |
|  | GO:0007568 | aging | 1.98×10^-21^ | 7.19×10^-19^ | CREB1, MAPK14, PTGS2, MAPK1, MAPK8, CAT, BCL2, AKT1, JUN, TIMP1, HMGCR, TP63, CDK1, CYP1A1, ADRA1A, CHEK1, CASP9, SREBF1, ICAM1, MAPK3, SOD1, CDK2, RELA, PPP3CA, SLC6A3, VCAM1 | 26 |
|  | GO:0048545 | response to steroid hormone | 1.98×10^-21^ | 7.19×10^-19^ | GSTP1, EGFR, PGR, CASP3, PTGS2, CA2, BCL2, AR, BAD, TP63, RXRB, UGT1A1, ESR1, CASP9, SREBF1, ICAM1, PPARD, RXRA, MAOB, RELA, AKR1C3, NR3C2, NCOA2, CALM1, ESR2, PPARA | 26 |
|  | GO:0097305 | response to alcohol | 2.21×10^-19^ | 6.68×10^-17^ | GSTP1, SLC2A4, CAT, F7, AHR, AKT1, HMGCR, BAD, CDK1, HTR3A, UGT1A1, SREBF1, ICAM1, OPRM1, SOD1, MAOB, AKR1C3, CES1, CALM1, SLC6A3, PPARA, VCAM1 | 22 |
|  | GO:0000302 | response to reactive oxygen species | 2.72×10^-19^ | 7.03×10^-17^ | GSTP1, EGFR, CASP3, STAT1, MAPK1, MAPK8, CAT, BCL2, AKT1, JUN, HMOX1, CYP1B1, BAD, MMP9, CDK1, NCF1, MAPK3, MET, SOD1, RELA, AKR1C3 | 21 |
|  | GO:0062197 | cellular response to chemical stress | 4.68×10^-19^ | 1.06×10^-16^ | EGFR, CASP3, SLC2A4, PTGS2, MAPK1, MAPK8, ABCC1, BCL2, AKT1, JUN, HMOX1, CYP1B1, BAD, MMP9, CDK1, NCF1, GSR, AKR1B1, MAPK3, MET, SOD1, RELA, AKR1C3, ALOX5 | 24 |
|  | GO:0006979 | response to oxidative stress | 1.92×10^-18^ | 3.81×10^-16^ | GSTP1, EGFR, CASP3, PTGS2, STAT1, MAPK1, MAPK8, CAT, ABCC1, BCL2, AKT1, JUN, HMOX1, CYP1B1, BAD, PTGS1, MMP9, CDK1, NCF1, GSR, MAPK3, MET, SOD1, RELA, AKR1C3, ALOX5 | 26 |
|  | GO:0008202 | steroid metabolic process | 2.10×10^-18^ | 3.81×10^-16^ | LDLR, CAT, CYP19A1, SOAT2, CYP1A2, HMGCR, CYP1B1, CYP1A1, SOAT1, UGT1A1, ESR1, AKR1B1, AKR1C1, SREBF1, APOB, PPARD, SOD1, RXRA, AKR1C3, NR1I2, CES1, CYP3A4, IL4 | 23 |
| CC | GO:0045121 | membrane raft | 9.13×10^-14^ | 8.17×10^-12^ | EGFR, CASP3, SLC2A4, PTGS2, MAPK1, IKBKB, SLC6A4, HMOX1, DPP4, KDR, ADRA1A, SELE, ICAM1, MAPK3, OPRM1, OLR1, OPRD1, SLC6A3, ADRA1B | 19 |
|  | GO:0098857 | membrane microdomain | 9.13×10^-14^ | 8.17×10^-12^ | EGFR, CASP3, SLC2A4, PTGS2, MAPK1, IKBKB, SLC6A4, HMOX1, DPP4, KDR, ADRA1A, SELE, ICAM1, MAPK3, OPRM1, OLR1, OPRD1, SLC6A3, ADRA1B | 19 |
|  | GO:0045211 | postsynaptic membrane | 9.39×10^-10^ | 5.60×10^-8^ | CHRM3, EPHB2, SLC6A4, CHRM2, CHRM4, ADRA2C, HTR3A, CHRM1, ADRA1A, CHRM5, GABRA1, OPRM1, OPRD1, SLC6A3 | 14 |
|  | GO:0099055 | integral component of postsynaptic membrane | 1.76×10^-9^ | 7.85×10^-8^ | EPHB2, SLC6A4, ADRA2C, HTR3A, CHRM1, ADRA1A, GABRA1, OPRM1, OPRD1, SLC6A3 | 10 |
|  | GO:0098936 | intrinsic component of postsynaptic membrane | 2.65×10^-9^ | 9.47×10^-8^ | EPHB2, SLC6A4, ADRA2C, HTR3A, CHRM1, ADRA1A, GABRA1, OPRM1, OPRD1, SLC6A3 | 10 |
|  | GO:0099056 | integral component of presynaptic membrane | 4.84×10^-9^ | 1.44×10^-7^ | EPHB2, SLC6A4, HTR3A, CHRM1, ADRA1A, OPRM1, OPRD1, SLC6A3 | 8 |
|  | GO:0098889 | intrinsic component of presynaptic membrane | 1.36×10^-8^ | 3.48×10^-7^ | EPHB2, SLC6A4, HTR3A, CHRM1, ADRA1A, OPRM1, OPRD1, SLC6A3 | 8 |
|  | GO:0099699 | integral component of synaptic membrane | 1.96×10^-8^ | 4.38×10^-7^ | EPHB2, SLC6A4, ADRA2C, HTR3A, CHRM1, ADRA1A, GABRA1, OPRM1, OPRD1, SLC6A3 | 10 |
|  | GO:0005901 | caveola | 3.37×10^-8^ | 6.70×10^-7^ | PTGS2, MAPK1, HMOX1, ADRA1A, SELE, MAPK3, SLC6A3, ADRA1B | 8 |
|  | GO:0099240 | intrinsic component of synaptic membrane | 4.33×10^-8^ | 7.74×10^-7^ | EPHB2, SLC6A4, ADRA2C, HTR3A, CHRM1, ADRA1A, GABRA1, OPRM1, OPRD1, SLC6A3 | 10 |
| MF | GO:0004879 | nuclear receptor activity | 9.12×10^-20^ | 1.29×10^-17^ | PGR, NR1I3, PPARG, AHR, AR, RXRB, ESR1, SREBF1, PPARD, RXRA, NR1I2, NR3C2, ESR2, PPARA | 14 |
|  | GO:0098531 | ligand-activated transcription factor activity | 9.12×10^-20^ | 1.29×10^-17^ | PGR, NR1I3, PPARG, AHR, AR, RXRB, ESR1, SREBF1, PPARD, RXRA, NR1I2, NR3C2, ESR2, PPARA | 14 |
|  | GO:0008227 | G protein-coupled amine receptor activity | 1.37×10^-14^ | 1.29×10^-12^ | CHRM3, CHRM2, CHRM4, ADRA1D, ADRA2C, CHRM1, ADRA1A, CHRM5, ADRB2, ADRB1, ADRA1B | 11 |
|  | GO:0003707 | steroid hormone receptor activity | 1.25×10^-12^ | 8.83×10^-11^ | PGR, RXRB, ESR1, PPARD, RXRA, NR3C2, ESR2, PPARA | 8 |
|  | GO:0099528 | G protein-coupled neurotransmitter receptor activity | 1.09×10^-11^ | 6.15×10^-10^ | CHRM3, CHRM2, CHRM4, CHRM1, CHRM5, ADRB1 | 6 |
|  | GO:0001223 | transcription coactivator binding | 1.71×10^-11^ | 8.08×10^-10^ | CREB1, PGR, AHR, AR, ESR1, PPARD, RELA, PPARA | 8 |
|  | GO:0019902 | phosphatase binding | 2.94×10^-10^ | 1.19×10^-8^ | EGFR, MAPK14, STAT1, MAPK1, BCL2, AKT1, HMGCR, BAD, MAPK3, MET, SOD1, SLC6A3, PPARA | 13 |
|  | GO:0033218 | amide binding | 4.94×10^-10^ | 1.75×10^-8^ | LDLR, PLA2G4A, ACHE, CAT, EPHB2, PPARG, SOAT2, GSTM1, FASN, SOAT1, GSTM2, ADRB2, OPRM1, RXRA, OPRD1, RELA, PPP3CA | 17 |
|  | GO:0001221 | transcription coregulator binding | 1.37×10^-9^ | 4.30×10^-8^ | CREB1, PGR, STAT1, AHR, AR, ESR1, PPARD, RELA, PPARA | 9 |
|  | GO:0098960 | postsynaptic neurotransmitter receptor activity | 4.71×10^-9^ | 1.33×10^-7^ | CHRM3, CHRM2, CHRM4, CHRM1, CHRM5, GABRA1, DRD1, ADRB1 | 8 |
| KEGG | hsa05417 | Lipid and atherosclerosis | 8.08×10^-23^ | 7.14×10^-21^ | LDLR, MAPK14, CASP3, MAPK1, IKBKB, MAPK8, PPARG, BCL2, MMP1, AKT1, JUN, BAD, BAX, MMP9, CYP1A1, NCF1, SELE, RXRB, CASP9, APOB, HSP90AA1, ICAM1, MAPK3, OLR1, GSK3B, RXRA, RELA, PPP3CA, CALM1, VCAM1 | 30 |
|  | hsa05207 | Chemical carcinogenesis - receptor activation | 1.27×10^-20^ | 5.63×10^-19^ | EGFR, CREB1, PGR, NR1I3, MAPK1, BCL2, AHR, CYP1A2, AKT1, JUN, AR, GSTM1, CYP1B1, BAD, CYP1A1, RXRB, UGT1A1, ESR1, GSTM2, ADRB2, HSP90AA1, MAPK3, RXRA, RELA, CYP3A4, ADRB1, ESR2, PPARA | 28 |
|  | hsa05215 | Prostate cancer | 7.36×10^-15^ | 2.17×10^-13^ | GSTP1, EGFR, CREB1, MAPK1, IKBKB, BCL2, AKT1, AR, INSRR, BAD, MMP9, CASP9, HSP90AA1, MAPK3, GSK3B, CDK2, RELA | 17 |
|  | hsa05208 | Chemical carcinogenesis - reactive oxygen species | 1.36×10^-14^ | 3.01×10^-13^ | EGFR, MAPK14, MAPK1, IKBKB, MAPK8, CAT, AHR, CYP1A2, AKT1, JUN, HMOX1, GSTM1, CYP1B1, BAD, CYP1A1, NCF1, AKR1C1, GSTM2, MAPK3, MET, SOD1, RELA, AKR1C3 | 23 |
|  | hsa05418 | Fluid shear stress and atherosclerosis | 1.97×10^-14^ | 3.48×10^-13^ | GSTP1, MAPK14, IKBKB, MAPK8, BCL2, AKT1, JUN, HMOX1, GSTM1, KDR, MMP9, NCF1, SELE, GSTM2, HSP90AA1, ICAM1, RELA, CALM1, VCAM1 | 19 |
|  | hsa05161 | Hepatitis B | 3.87×10^-12^ | 5.70×10^-11^ | CREB1, MAPK14, CASP3, STAT1, MAPK1, IKBKB, MAPK8, BCL2, AKT1, JUN, BAD, BAX, MMP9, CCNA2, CASP9, MAPK3, CDK2, RELA | 18 |
|  | hsa04668 | TNF signaling pathway | 1.85×10^-11^ | 2.04×10^-10^ | CREB1, MAPK14, CASP3, PTGS2, MAPK1, IKBKB, MAPK8, AKT1, JUN, MMP9, SELE, ICAM1, MAPK3, RELA, VCAM1 | 15 |
|  | hsa05145 | Toxoplasmosis | 1.85×10^-11^ | 2.04×10^-10^ | LDLR, MAPK14, CASP3, STAT1, MAPK1, IKBKB, MAPK8, BCL2, AKT1, BAD, CASP9, MAPK3, NOS2, RELA, ALOX5 | 15 |
|  | hsa04657 | IL-17 signaling pathway | 2.09×10^-11^ | 2.05×10^-10^ | MAPK14, CASP3, PTGS2, MAPK1, IKBKB, MAPK8, MMP1, JUN, MMP9, HSP90AA1, MAPK3, GSK3B, RELA, IL4 | 14 |
|  | hsa04933 | AGE-RAGE signaling pathway in diabetic complications | 4.95×10^-11^ | 4.37×10^-10^ | MAPK14, CASP3, STAT1, MAPK1, MAPK8, BCL2, AKT1, JUN, BAX, SELE, ICAM1, MAPK3, RELA, VCAM1 | 14 |

**Table S11** Selected pathways to participate in the conduction of CTPDN.

| Number | Pathway name | *q* Value | Participate target | Relationship with liver disease | Relationship with gastric mucosa disease | Reference |
| --- | --- | --- | --- | --- | --- | --- |
| 1 | Lipid and atherosclerosis | 7.14×10^-21^ | MAPK14, CASP3, MAPK1, IKBKB, MAPK8, PPARG, AKT1, JUN, MMP9, CASP9, ICAM1, MAPK3 | T | F | [1,2] |
| 2 | Chemical carcinogenesis-receptor activation | 5.63×10^-19^ | EGFR, PGR, MAPK1, AKT1, JUN, AR, ESR1, MAPK3, PPARA | T | T | [3,4] |
| 3 | Chemical carcinogenesis-reactive oxygen species | 3.01×10^-13^ | EGFR, MAPK14, MAPK1, IKBKB, MAPK8, CAT, AKT1, JUN, HMOX1, MAPK3 | T | T | [5,6] |
| 4 | Fluid shear stress and atherosclerosis | 3.48×10^-13^ | MAPK14, IKBKB, MAPK8, AKT1 JUN, HMOX1, MMP9, ICAM1 | T | F | [7] |
| 5 | Hepatitis B | 5.70×10^-11^ | MAPK14, CASP3, STAT1, MAPK1, IKBKB, MAPK8, AKT1, JUN, MMP9, CASP9, MAPK3 | T | F | [8] |
| 6 | TNF signaling pathway | 2.04×10^-10^ | MAPK14, CASP3, PTGS2, MAPK1, IKBKB, MAPK8, AKT1, JUN, MMP9, ICAM1, MAPK3 | T | T | [9,10] |
| 7 | IL-17 signaling pathway | 2.05×10^-10^ | MAPK14, CASP3, PTGS2, MAPK1, IKBKB, MAPK8, JUN, MMP9, MAPK3 | T | T | [11,12] |
| 8 | Th17 cell differentiation | 1.05×10^-9^ | MAPK14, STAT1, MAPK1, IKBKB, MAPK8, JUN, MAPK3 | T | T | [13,14] |
| 9 | Hepatitis C | 1.71×10^-9^ | EGFR, CASP3, STAT1, MAPK1, IKBKB, AKT1, CASP9, MAPK3, PPARA | T | F | [15] |
| 10 | Estrogen signaling pathway | 2.43×10^-9^ | EGFR, PGR, MAPK1, AKT1, JUN, MMP9, ESR1, MAPK3 | F | T | [16] |
| 11 | Endocrine resistance | 2.99×10^-9^ | EGFR, MAPK14, MAPK1, MAPK8, AKT1, JUN, MMP9, ESR1, MAPK3 | T | T | [17,18] |
| 12 | VEGF signaling pathway | 1.96×10^-8^ | MAPK14, PTGS2, MAPK1, AKT1, CASP9, MAPK3 | T | T | [19,20] |
| 13 | cGMP-PKG signaling pathway | 1.34×10^-7^ | MAPK1, AKT1, MAPK3 | F | T | [21] |
| 14 | EGFR tyrosine kinase inhibitor resistance | 2.39×10^-7^ | EGFR, MAPK1, AKT1, MAPK3 | T | T | [22,23] |
| 15 | Calcium signaling pathway | 3.06×10^-7^ | EGFR, NOS2 | T | T | [24,25] |
| 16 | T cell receptor signaling pathway | 3.28×10^-7^ | MAPK14, MAPK1, IKBKB, MAPK8, AKT1, JUN, MAPK3 | T | T | [26,27] |
| 17 | Non-alcoholic fatty liver disease | 3.40×10^-7^ | MAPK14, CASP3, IKBKB, MAPK8, PPARG, AKT1, JUN, PPARA | T | T | [28] |

*T means pathway has relative with that disease, while F means no relatives exist based on previous study.*

**Table S12** ADMET prediction results and important parameters in CTPDN of candidate compounds.

| Component | AS | CYP2D6 | Hepatotoxic | PPB | BBB | Liver disease | | | Gastric mucosa disease | | |
| --- | --- | --- | --- | --- | --- | --- | --- | --- | --- | --- | --- |
|  |  |  |  |  |  | Target | Pathway | Score | Target | Pathway | Score |
| 1-(4-Hydroxybenzyl)-4-Methoxy-9,10-Dihydrophenanthrene-2,7-Diol | -5.254 | 2.4844 | 1.6707 | -1.2565 | 0.318 | AKT1, STAT1 | 4 | 1.6372 | AKT1 | 1 | 1.6372 |
| 2,3,4,7-Tetramethoxyphenanthrene | -3.352 | -9.27505 | -13.4369 | -15.98 |  | CAT | 2 | 1.5971 | CAT | 2 | 1.5971 |
| Blespirol | -3.928 | -2.1507 | -0.9987 | 0.2330 | -0.716 | AKT1, STAT1 | 4 | 1.6429 | AKT1 | 1 | 1.6429 |
| Militarin | -4.472 | 1.8966 | -1.5420 | 1.6829 | 0.033 | AKT1, STAT1 | 4 | 1.6167 | AKT1 | 1 | 1.6167 |
| 3,5,6,7,8,3′,4′-Heptamethoxyflavone | -3.157 | 1.4530 | -1.5289 | -4.8989 | -0.97 | CAT | 2 | 1.6276 | CAT | 2 | 1.6276 |
| 5,7-Dihydroxy-2-(3-Hydroxy-4-Methoxyphenyl)Chroman-4-One | -4.225 | -0.1559 | -0.0058 | 3.9845 | 0.004 | AKT1, STAT1 | 4 | 1.6244 | AKT1 | 1 | 1.6244 |
| Citromitin | -5.673 | -0.3969 | 2.1250 | 3.3642 | -0.146 | AKT1, STAT1 | 4 | 1.6867 | AKT1 | 1 | 1.6867 |
| Hederagenin | -3.707 | -8.2531 | -11.8102 | -21.3962 |  | IKBKB, STAT1 | 4 | 1.6128 | IKBKB, STAT1 | 4 | 1.6128 |
| Hesperidin | -3.777 | -12.291 | -13.5345 | -22.9514 |  | AKT1, STAT1 | 4 | 1.6169 | AKT1 | 1 | 1.6169 |
| Narirutin | -4.19 | -11.5075 | -14.2977 | -22.7065 |  | HMOX1 | 2 | 1.6053 | HMOX1 | 2 | 1.6053 |
| Nobiletin | -4.355 | -8.2531 | -11.8102 | -20.7934 |  | AKT1, STAT1 | 4 | 1.6050 | AKT1 | 1 | 1.6050 |
| Sitosterol | -5.398 | -2.34486 | 2.93641 | 3.23188 | -0.141 | CAT | 2 | 1.6765 | CAT | 2 | 1.6765 |
| Tangeretin | -7.567 | -2.5794 | -10.6516 | 2.2590 |  | AKT1, STAT1 | 4 | 1.6763 | AKT1 | 1 | 1.6763 |
| 3,9-di-O-Methylnissolin | -4.106 | -3.0947 | -0.2145 | 3.7857 |  | AKT1, STAT1 | 4 | 1.6618 | AKT1 | 1 | 1.6618 |
| 7-O-Methylisomucronulatol | -6.778 | -1.3930 | 1.1268 | -1.3902 | 0.117 | AKT1, STAT1 | 4 | 1.6288 | AKT1 | 1 | 1.6288 |
| Astragaloside Ⅲ | -3.179 | -1.0931 | 0.4370 | -3.0587 | -0.638 | IKBKB, STAT1 | 4 | 1.6394 | IKBKB, STAT1 | 4 | 1.6394 |
| Astragaloside I | -2.503 | -6.2878 | -1.3092 | -16.4732 |  | CASP3, STAT1 | 4 | 1.6372 | CASP3, STAT1 | 4 | 1.6372 |
| Astragaloside II | -4.196 | -0.1220 | -1.9149 | 0.9680 | -0.489 | HMOX1 | 2 | 1.6091 | HMOX1 | 2 | 1.6091 |
| Astragaloside IV | -4.491 | -4.8559 | -3.9708 | -0.6124 |  | CAT | 2 | 1.6445 | CAT | 2 | 1.6445 |
| Betulinic acid | -3.767 | -2.0954 | -3.8674 | 0.4756 | -0.264 | IKBKB, STAT1 | 4 | 1.6295 | IKBKB, STAT1 | 4 | 1.6295 |
| Bifendate | -3.564 | -0.3597 | 2.3123 | -2.0066 | -0.234 | CAT | 2 | 1.6392 | CAT | 2 | 1.6392 |
| Calycosin | -6.335 | -2.8084 | -0.6243 | 5.6400 |  | AKT1, STAT1 | 4 | 1.6694 | AKT1 | 1 | 1.6694 |
| Calycosin-7-O-beta-D-Glucopyranoside | -5.651 | -1.4792 | 0.3370 | 1.1251 | 0.11 | CAT | 2 | 1.6605 | CAT | 2 | 1.6605 |
| Formononetin | -5.673 | -0.3969 | 2.1250 | 3.3642 | -0.146 | AKT1, STAT1 | 4 | 1.6867 | AKT1 | 1 | 1.6867 |
| Isoflavanone | -6.253 | -1.4431 | -9.2795 | -0.5783 |  | AKT1, STAT1 | 4 | 1.6922 | AKT1 | 1 | 1.6922 |
| Isorhamnetin | -2.942 | 0.5735 | -2.2081 | -5.9519 | -0.824 | AKT1, STAT1 | 4 | 1.6318 | AKT1 | 1 | 1.6318 |
| Kaempferol | -4.194 | -0.3581 | -4.9426 | 3.3677 | 0.353 | AKT1, STAT1 | 4 | 1.6699 | AKT1 | 1 | 1.6699 |
| Kumatakenin | -2.892 | -1.0625 | -1.0490 | -3.8472 |  | AKT1, STAT1 | 4 | 1.6231 | AKT1 | 1 | 1.6231 |
| Ononin | -2.589 | -2.2409 | 1.4083 | -4.6066 | -1.308 | AKT1, STAT1 | 4 | 1.6366 | AKT1 | 1 | 1.6366 |
| Angeloylgomisin O | -3.336 | -1.6145 | 1.8896 | -2.1574 | -0.792 | CAT | 2 | 1.6209 | CAT | 2 | 1.6209 |
| Besigomsin | -2.914 | -6.1389 | -5.6471 | -0.5646 | -1.155 | CAT | 2 | 1.6971 | CAT | 2 | 1.6971 |
| Deoxyharringtonine | -5.309 | 0.6462 | 3.0800 | 5.2633 | 0.388 | CAT | 2 | 1.6209 | CAT | 2 | 1.6209 |
| Deoxyshikonin | -4.177 | -4.6718 | -4.9151 | -18.4179 |  | AKT1, STAT1 | 4 | 1.6220 | AKT1 | 1 | 1.6220 |
| Gomisin G | -4.228 | -1.3458 | -1.5560 | 3.0973 | -0.478 | CAT | 2 | 1.6123 | CAT | 2 | 1.6123 |
| Gomisin J | -2.291 | -6.4265 | -0.1724 | -14.3378 |  | CAT | 2 | 1.6170 | CAT | 2 | 1.6170 |
| Gomisin R | -6.748 | -0.1166 | 1.5359 | 4.0376 | 0.503 | CAT | 2 | 1.6591 | CAT | 2 | 1.6591 |
| Longikaurin A | -5.234 | -1.2445 | 1.4674 | 0.9060 | -0.079 | CAT | 2 | 1.6779 | CAT | 2 | 1.6779 |
| Schisandrin C | -5.398 | -2.3449 | 2.9364 | 3.2319 | -0.141 | AKT1, STAT1 | 4 | 1.6730 | AKT1 | 1 | 1.6730 |
| Schisandrin | -6.001 | -0.1395 | 1.1644 | 1.6708 | 0.368 | CAT | 2 | 1.6672 | CAT | 2 | 1.6672 |
| Schisandrol B | -8.256 | -2.8260 | -8.2547 | 8.5940 |  | CAT | 2 | 1.6537 | CAT | 2 | 1.6537 |
| Schisanhenol | -4.294 | -2.5374 | 1.0236 | 2.9227 | -0.332 | CAT | 2 | 1.6122 | CAT | 2 | 1.6122 |
| Tigloylgomisin P | -6.227 | -0.9189 | 3.3188 | 3.5282 |  | CASP3, STAT1 | 4 | 1.6539 | CASP3, STAT1 | 4 | 1.6539 |

## 4 Figures


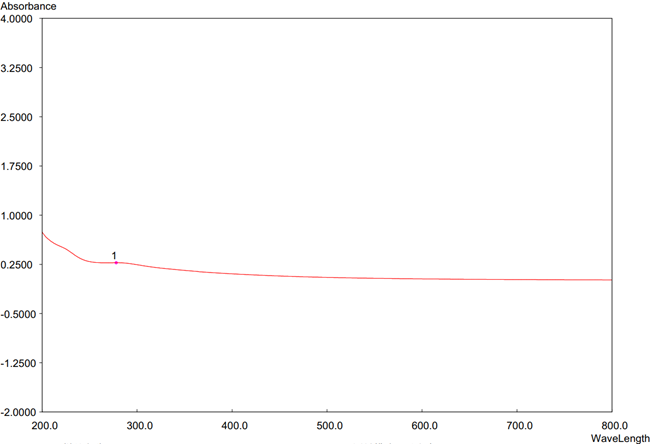


**Fig. S1** Full-wavelength scanning image of BWG aqueous solution.


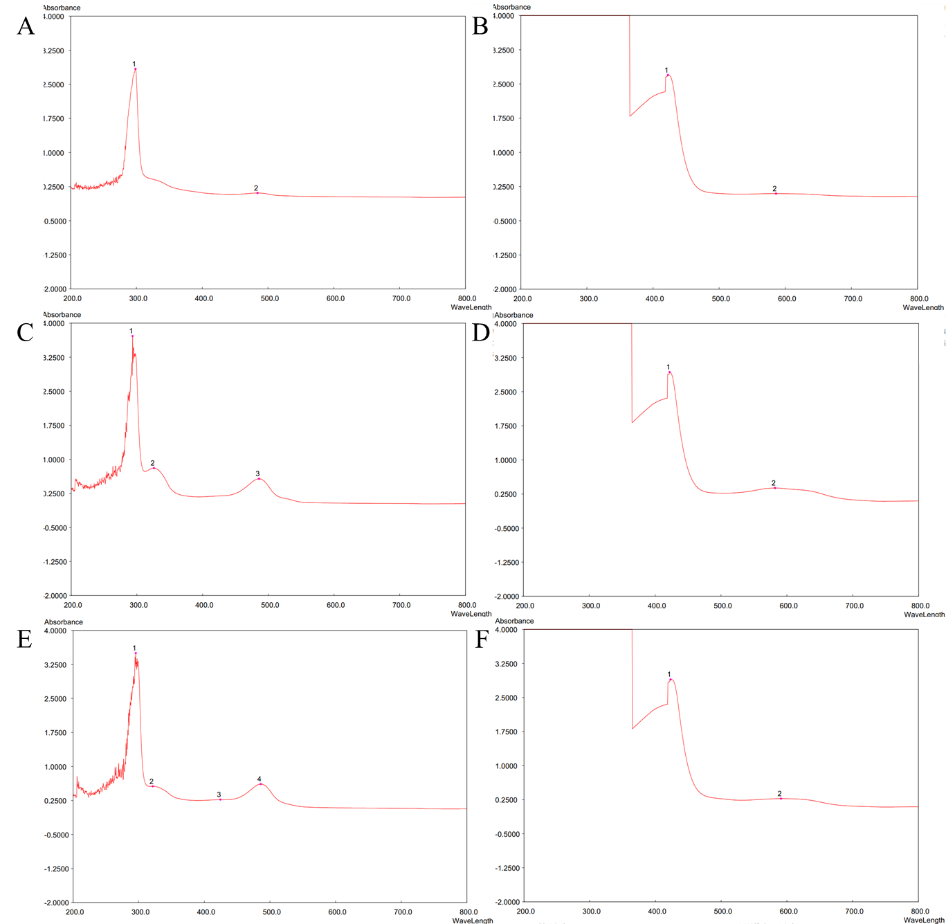


**Fig.S2** Full wavelength UV scanning diagram of phenol sulfuric acid method and anthrone sulfuric acid method. (A) Blank reagent under phenol-sulfuric acid method; (B) Blank reagent under anthrone-sulfuric acid method; (C) Color development of glucose reference under phenol-sulfuric acid method; (D) Color development of glucose reference under anthrone-sulfuric acid method; (E) Color development of BWG sample under phenol-sulfuric acid method; (F) Color development of BWG sample under anthrone-sulfuric acid method.


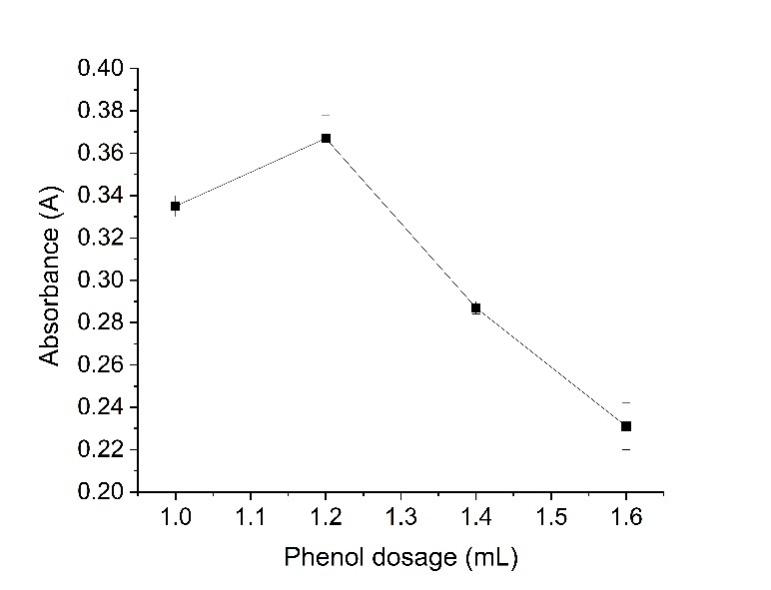


**Fig. S3** Investigation of phenol dosage.


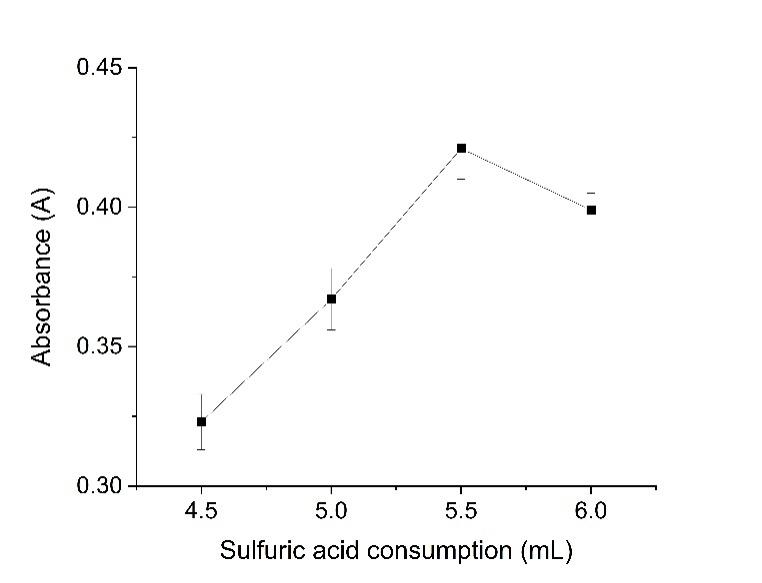


**Fig. S4** Investigation of sulfuric acid consumption.


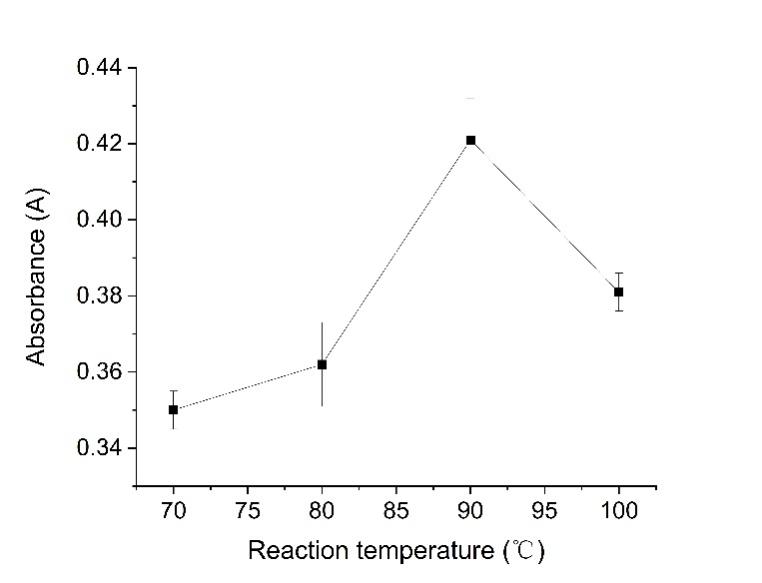


**Fig. S5** Investigation of reaction temperature.


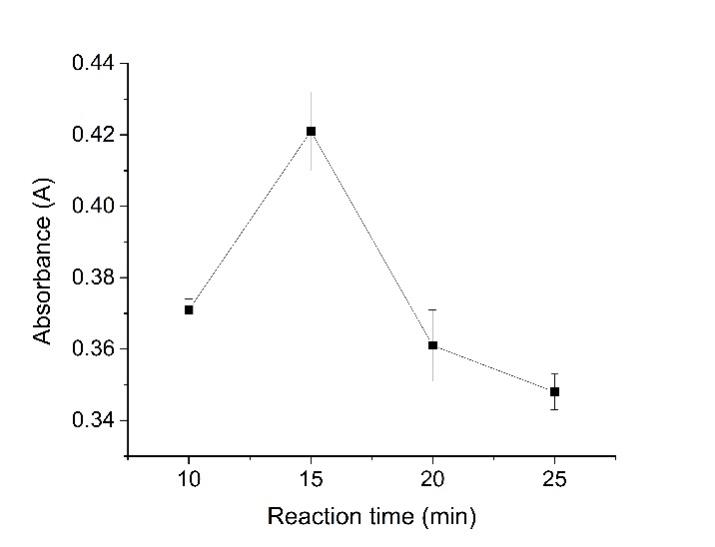


**Fig. S6** Investigation of reaction time.


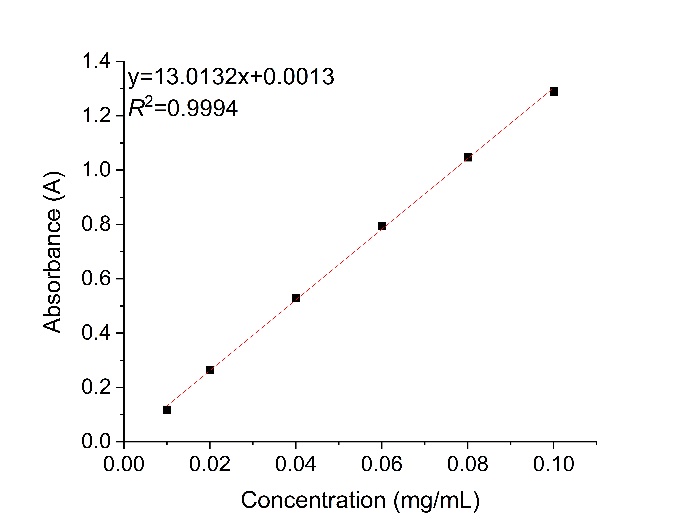


**Fig. S7** Standard curve for total sugar content determination.


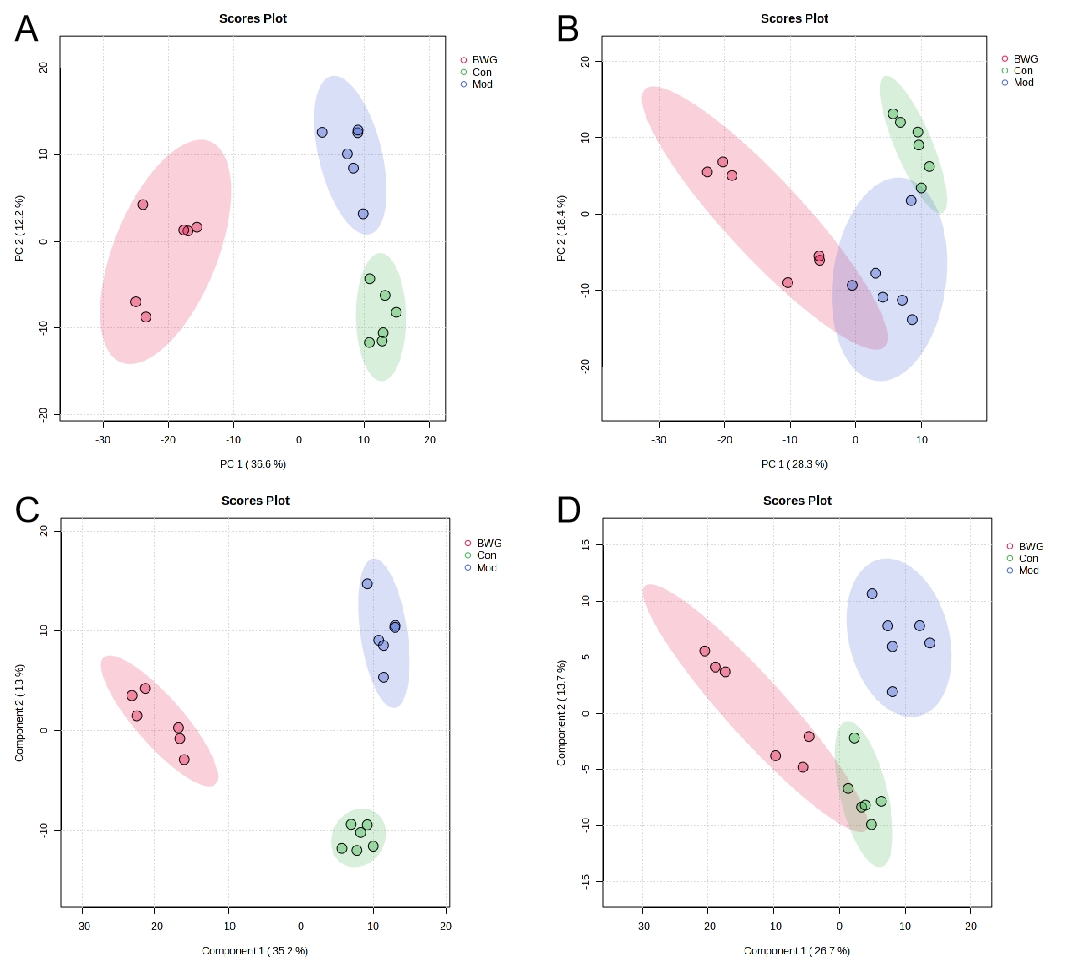


**Fig. S8** PCA and PLS-DA analysis for serum metabolites. (A) PCA analysis under negative ion mode, (B) PCA analysis under positive ion mode, (C) PLS-DA analysis under negative ion mode, (D) PLS-DA analysis under positive ion mode.


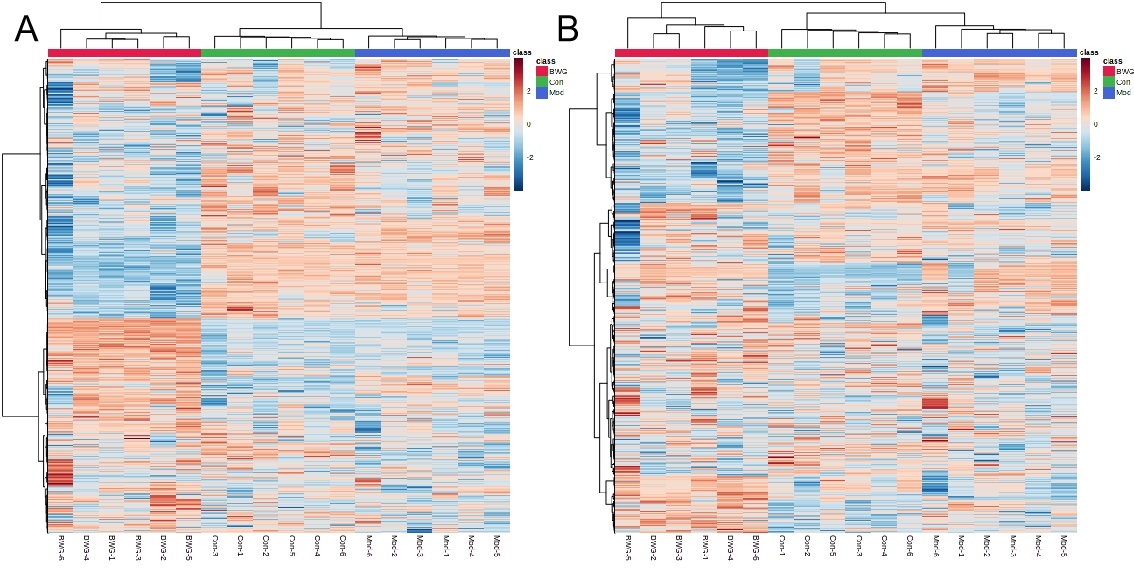


**Fig S9** Heatmap of metabolites detected under negative ion mode (A) and positive ion mode (B).


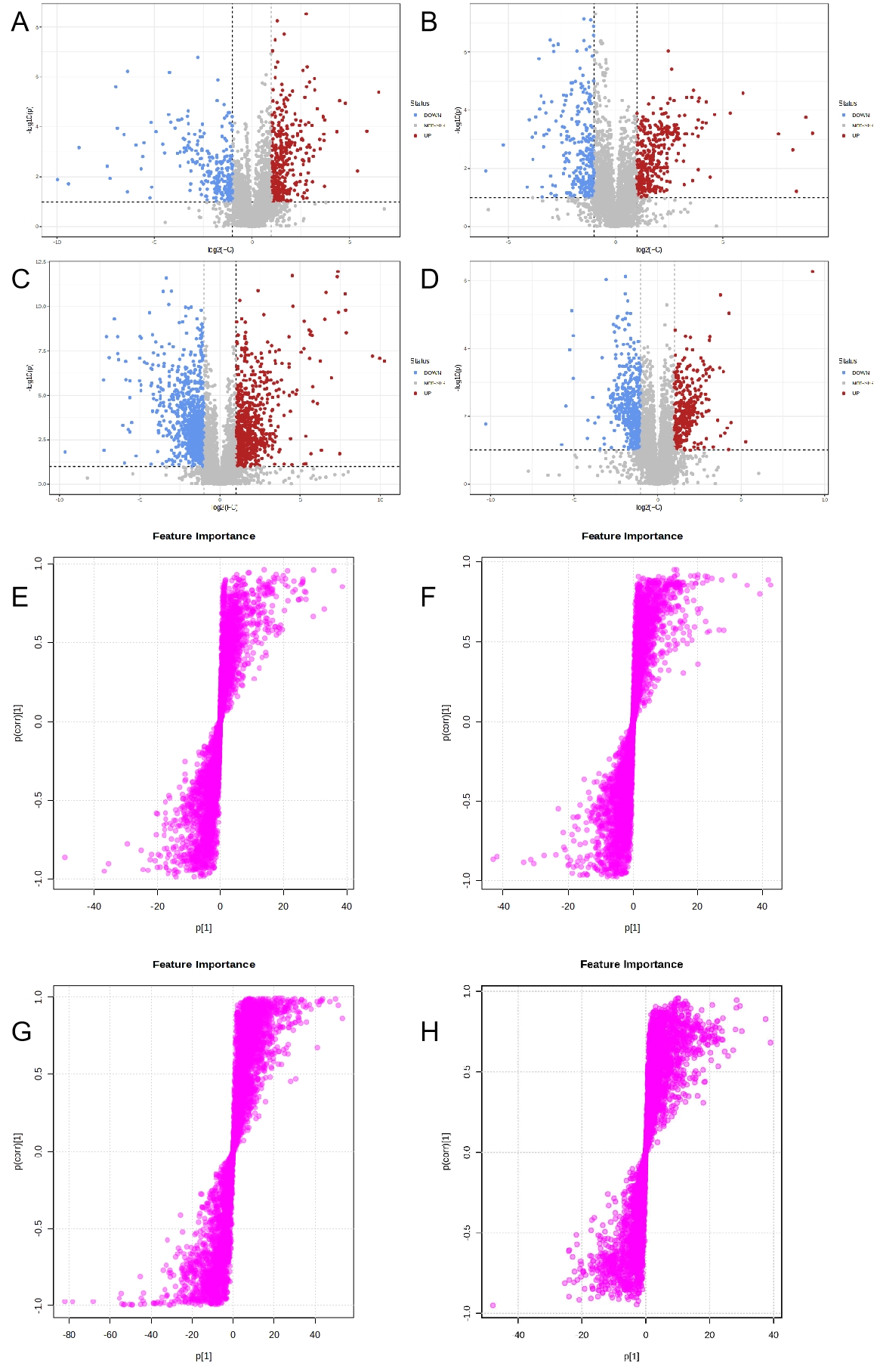


**Fig. S10** Analyses for identifying the differential metabolites. (A)~(D) Volcano diagram of Con vs Mod group under negative and positive ion mode, and Mod vs BWG group under negative and positive ion mode, respectively. (E)~(H) S-plot of OPLS-DA analysis of Con vs Mod group under negative and positive ion mode, and Mod vs BWG group under negative and positive ion mode, respectively.


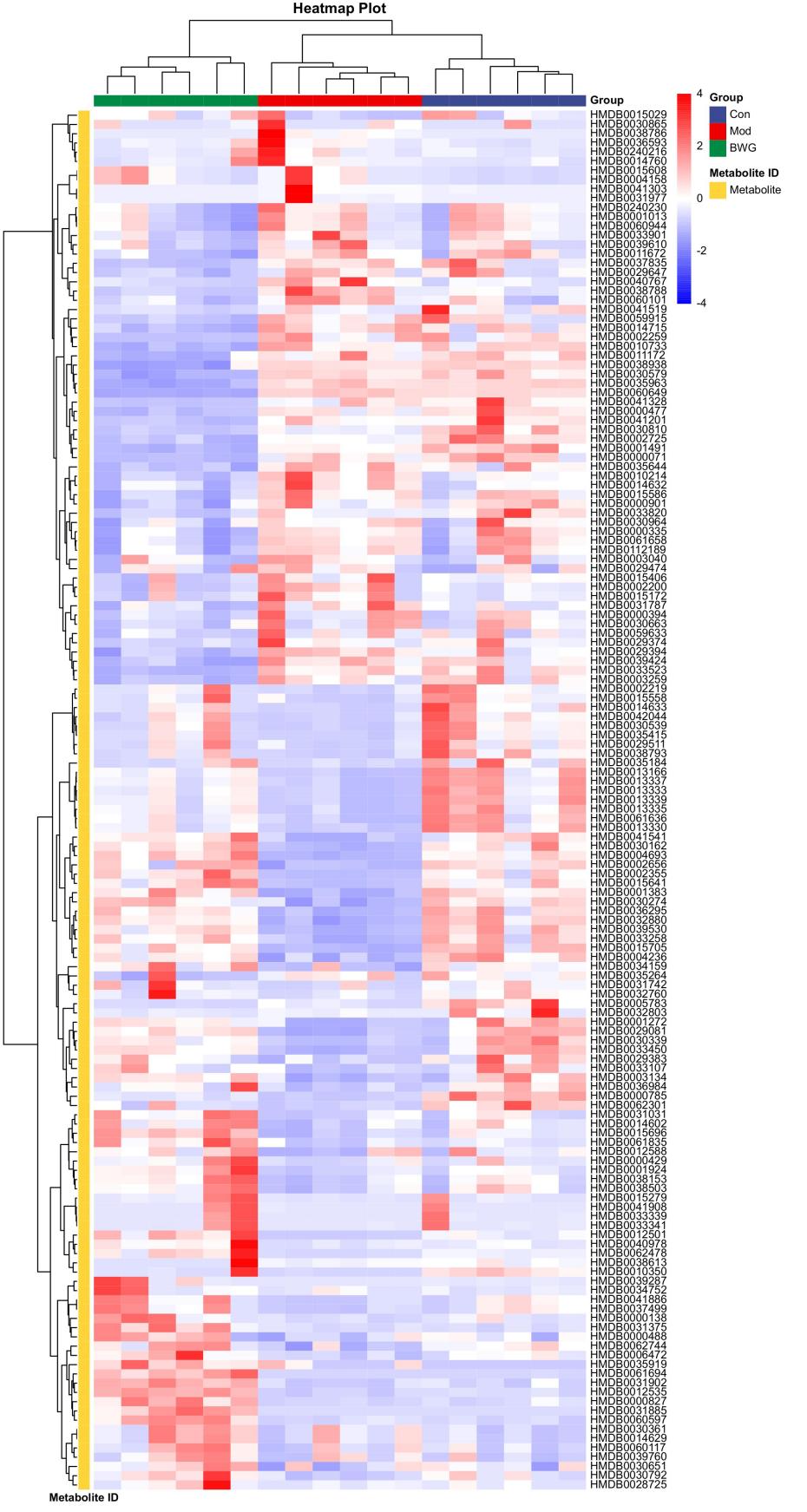


**Fig. S11** Heatmap of abundance of differential metabolites.


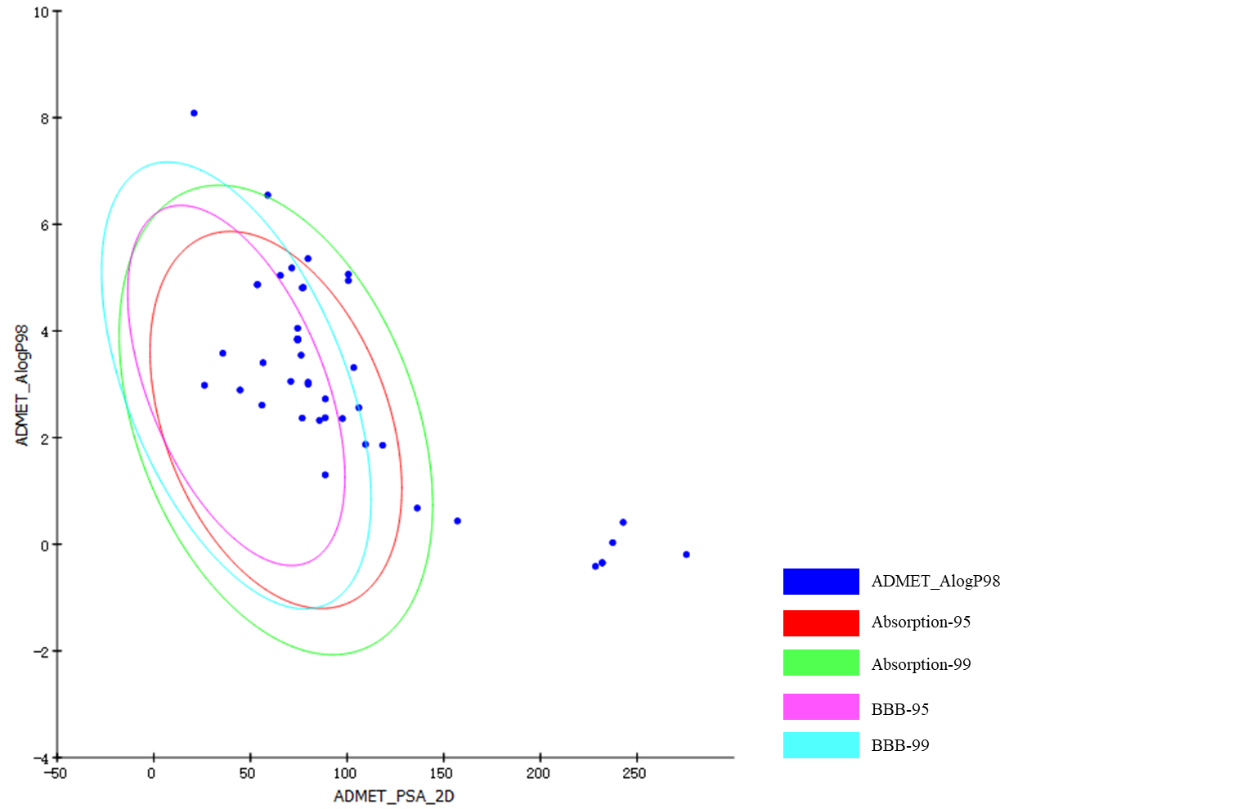


**Fig. S12** Prediction of functional components from BWG. Red and Green ellipse define areas are belong to well-absorbed component. As for the BBB model, the component fails to be in the ellipse, it means the prediction is considered unreliable.


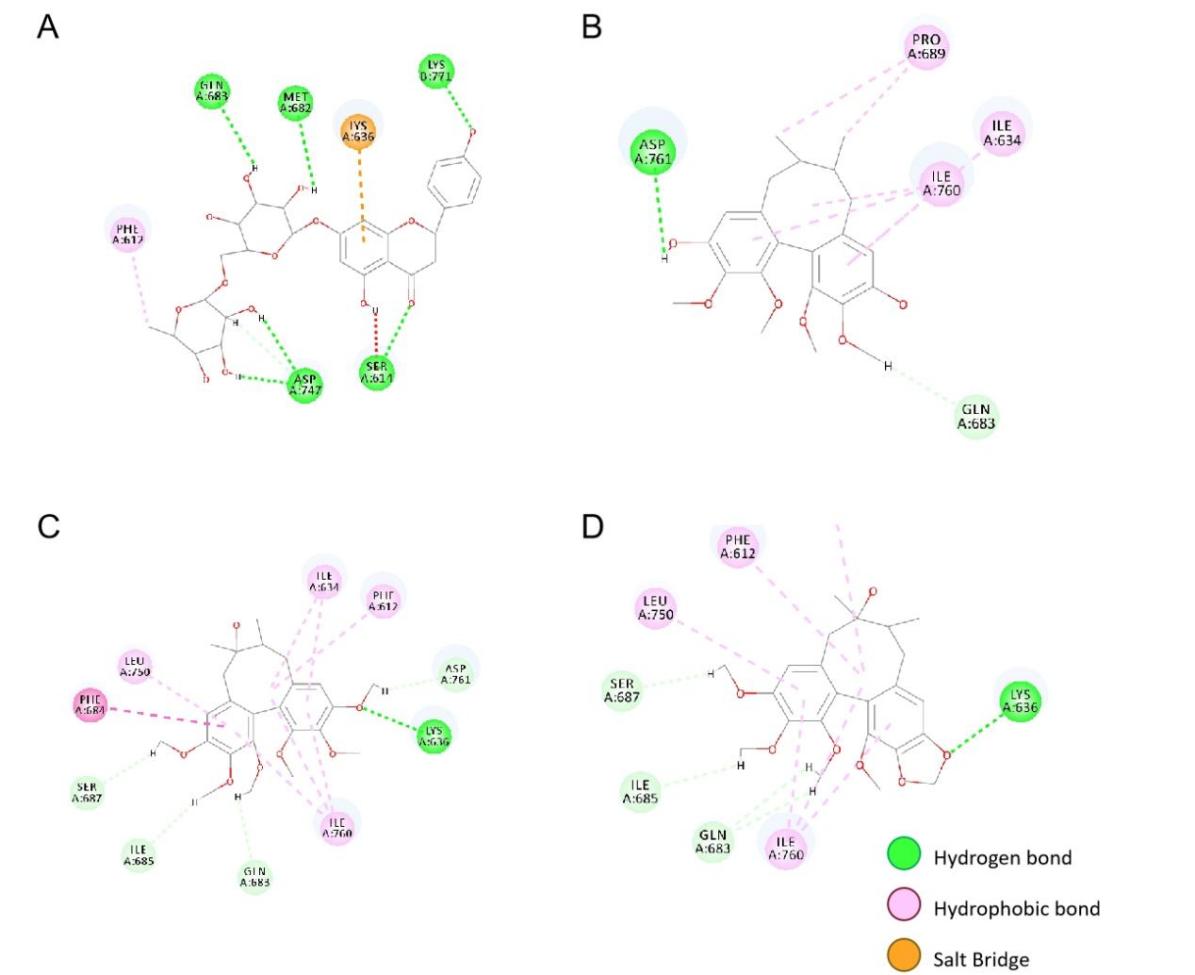


**Fig. S13** Molecular docking model of PI3K and effective compounds. (A) Narirutin, (B) Gomisin J, (C) Schisandrin, (D) Schisandrol B.


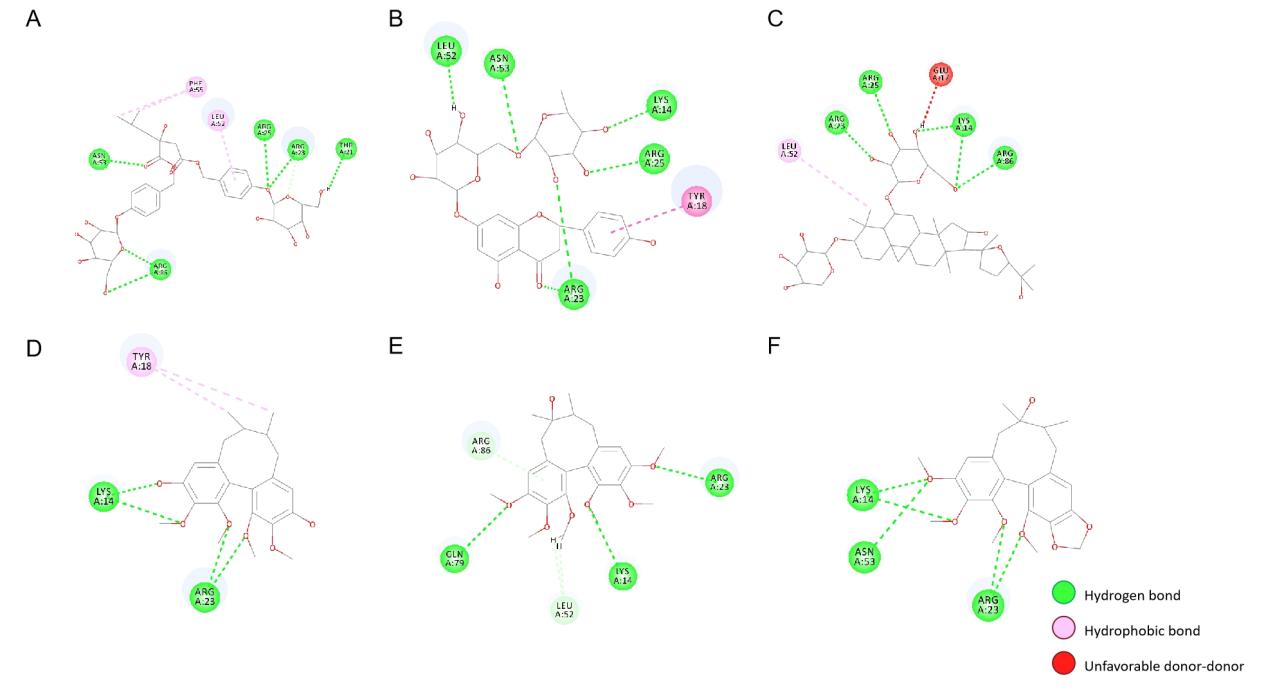


**Fig. S14** Molecular docking model of AKT1 and effective compounds. (A) 2,3,4,7-tetramethoxyphenanthrene, (B) Narirutin, (C) Astragaloside IV, (D) Gomisin J, (E) Schisandrin, (F) Schisandrol B.


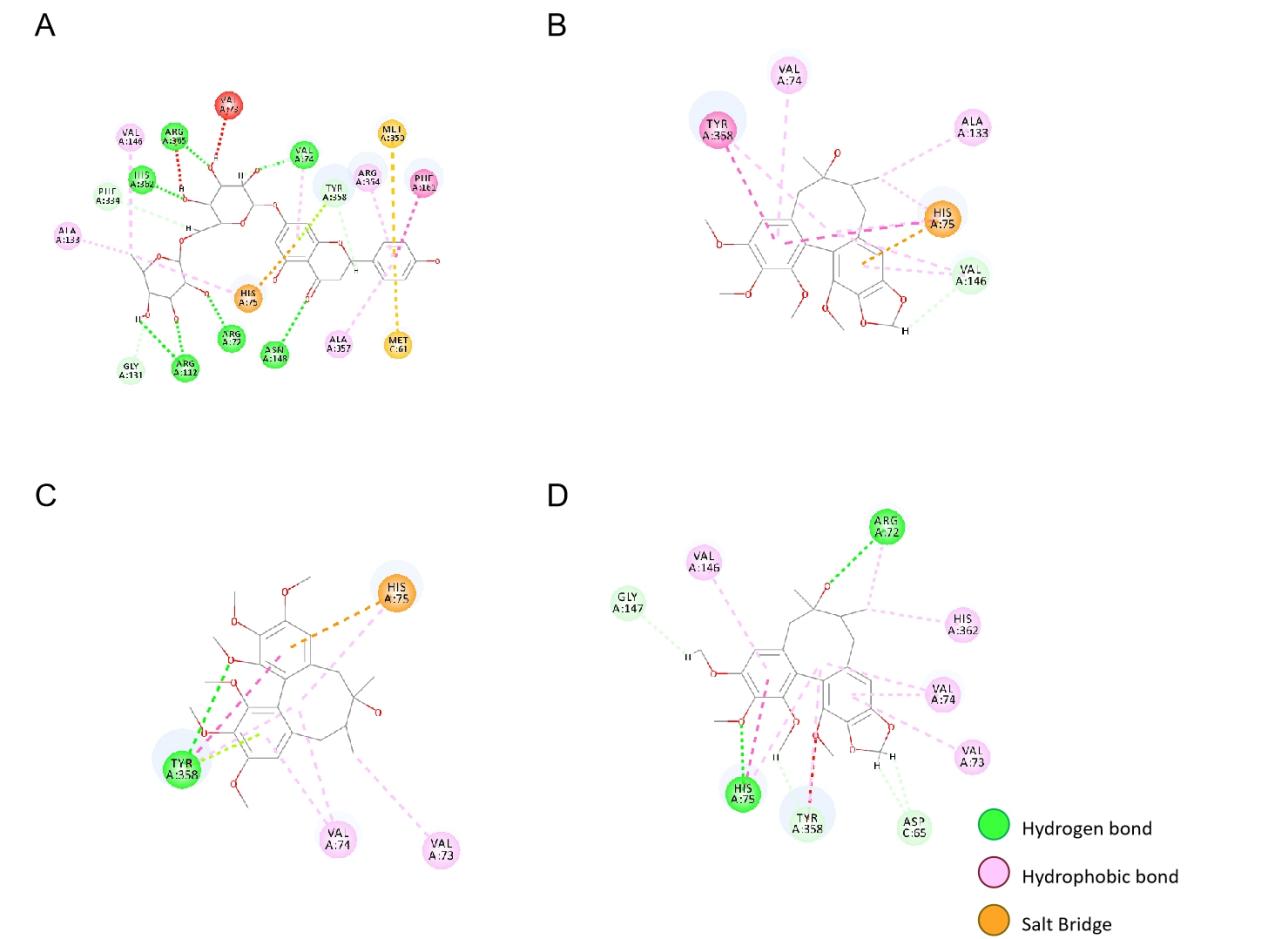


**Fig. S15** Molecular docking model of CAT and effective compounds. (A) Narirutin, (B) Gomisin J, (C) Schisandrin, (D) Schisandrol B.


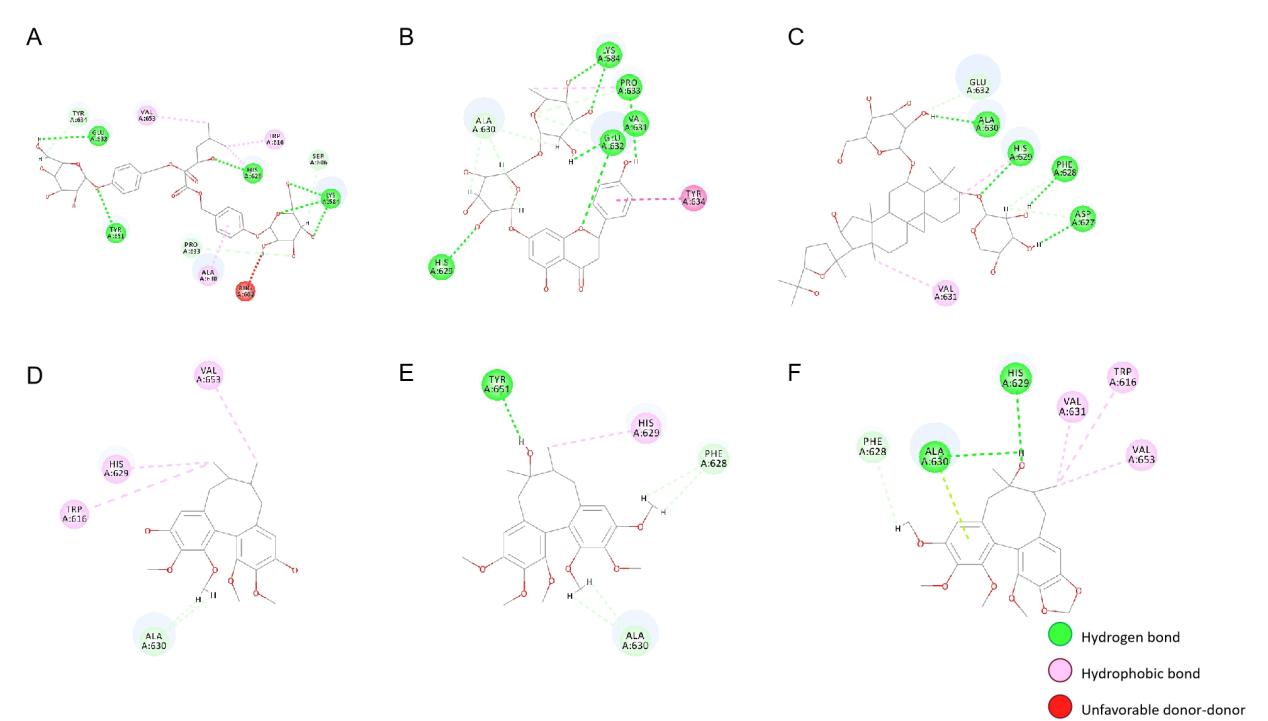


**Fig. S16** Molecular docking model of STAT1 and effective compounds. (A) 2,3,4,7-tetramethoxyphenanthrene, (B) Narirutin, (C) Astragaloside IV, (D) Gomisin J, (E) Schisandrin, (F) Schisandrol B.

Western blot full scan

**Fig. S17** STAT1


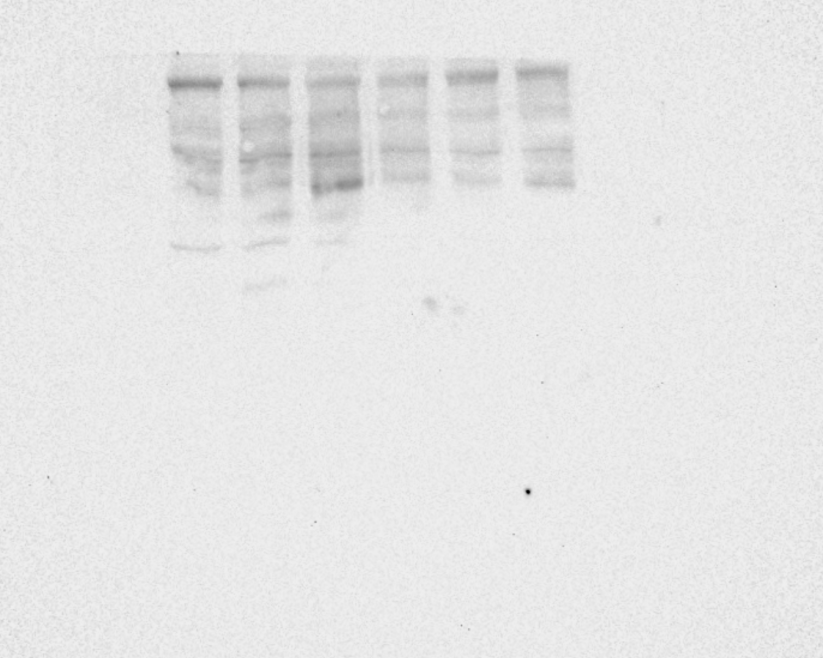


In Chemiluminescence mode


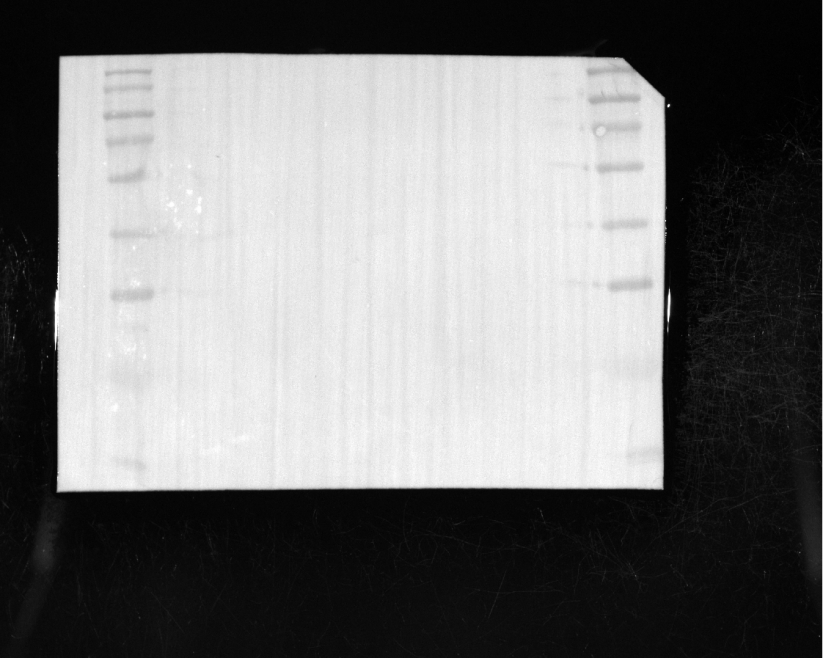


In Colorimetric mode

**Fig. S18** AKT1


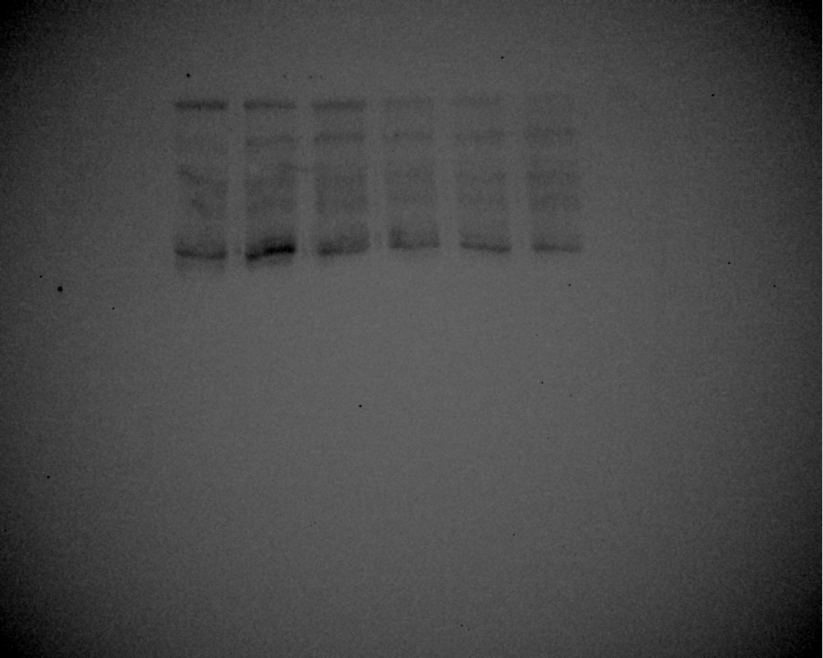


In Chemiluminescence mode


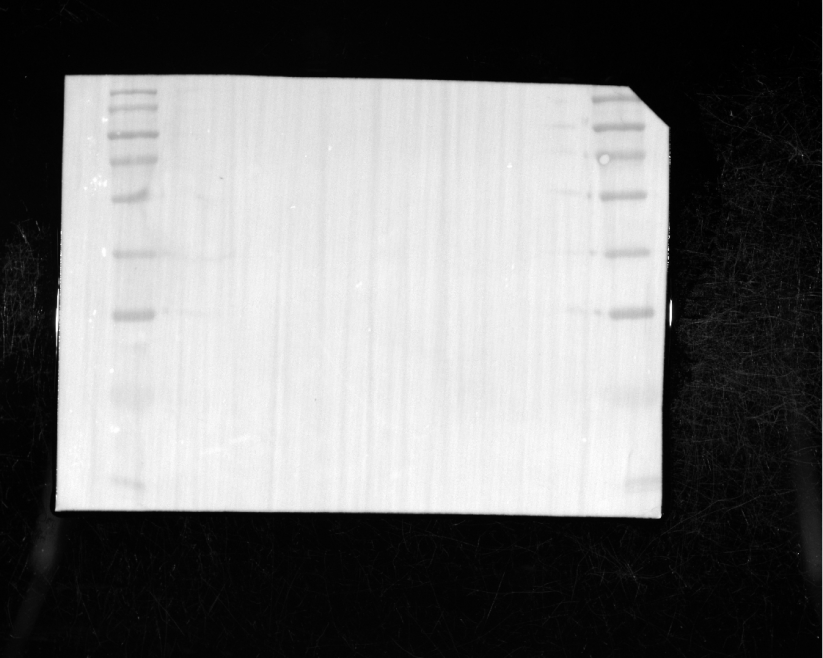


In Colorimetric mode

**Fig. S19** Catalase


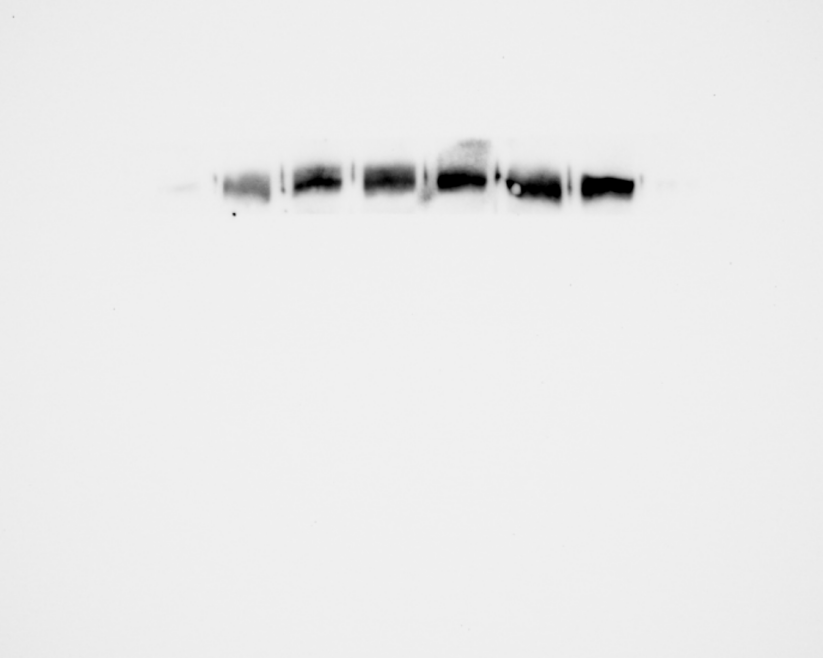


In Chemiluminescence mode


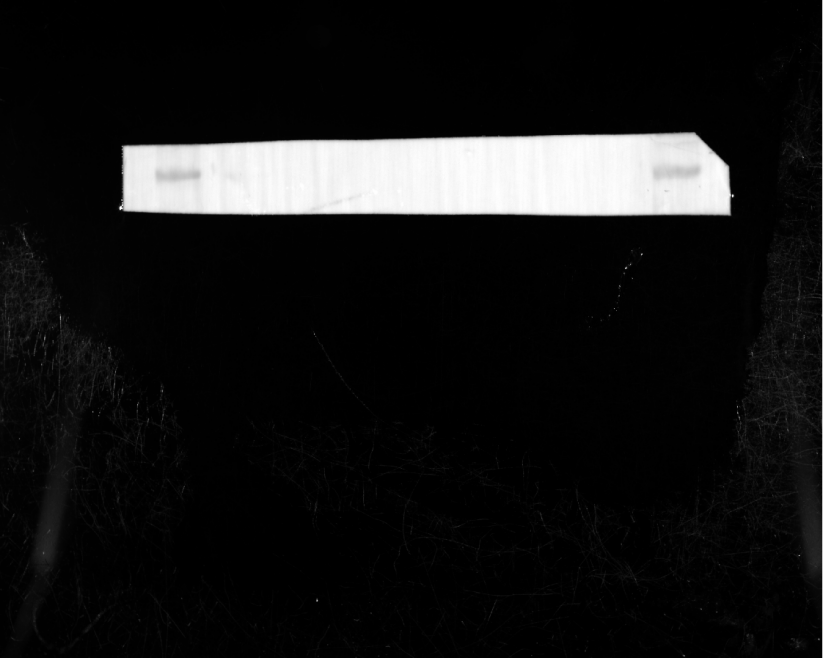


In Colorimetric mode

**Fig. S20** β-actin


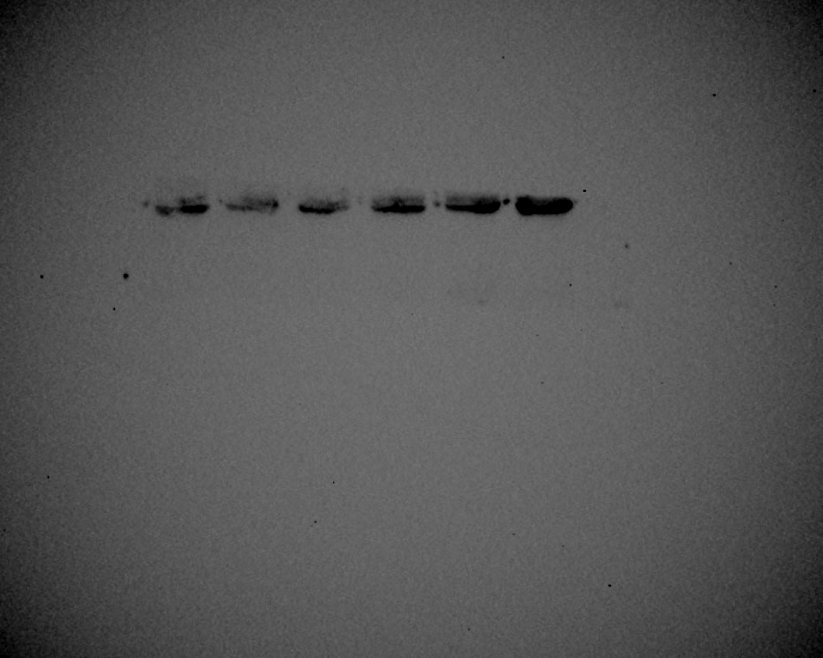


In Chemiluminescence mode


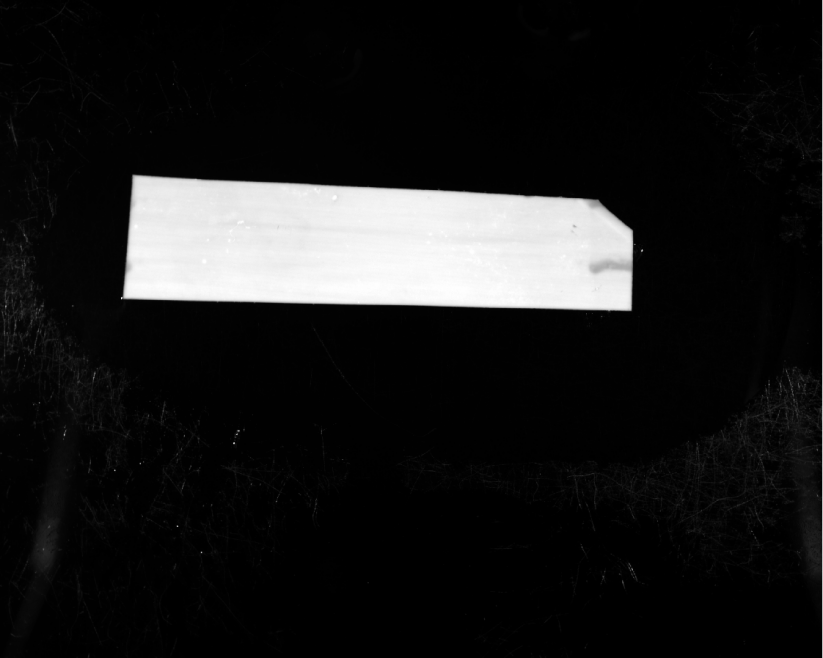


In Colorimetric mode

**Fig. S21** pAKT1 (Ser473)


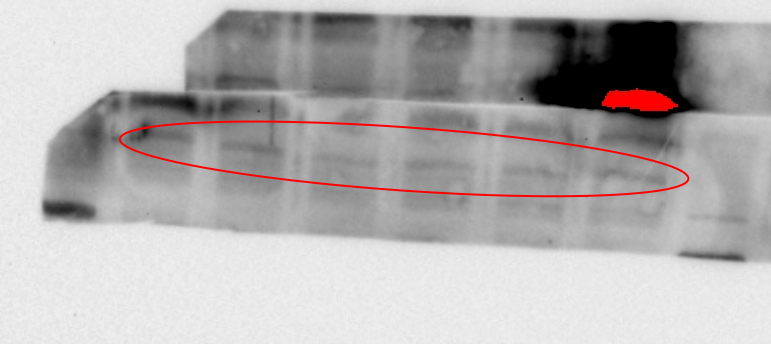


In Chemiluminescence mode


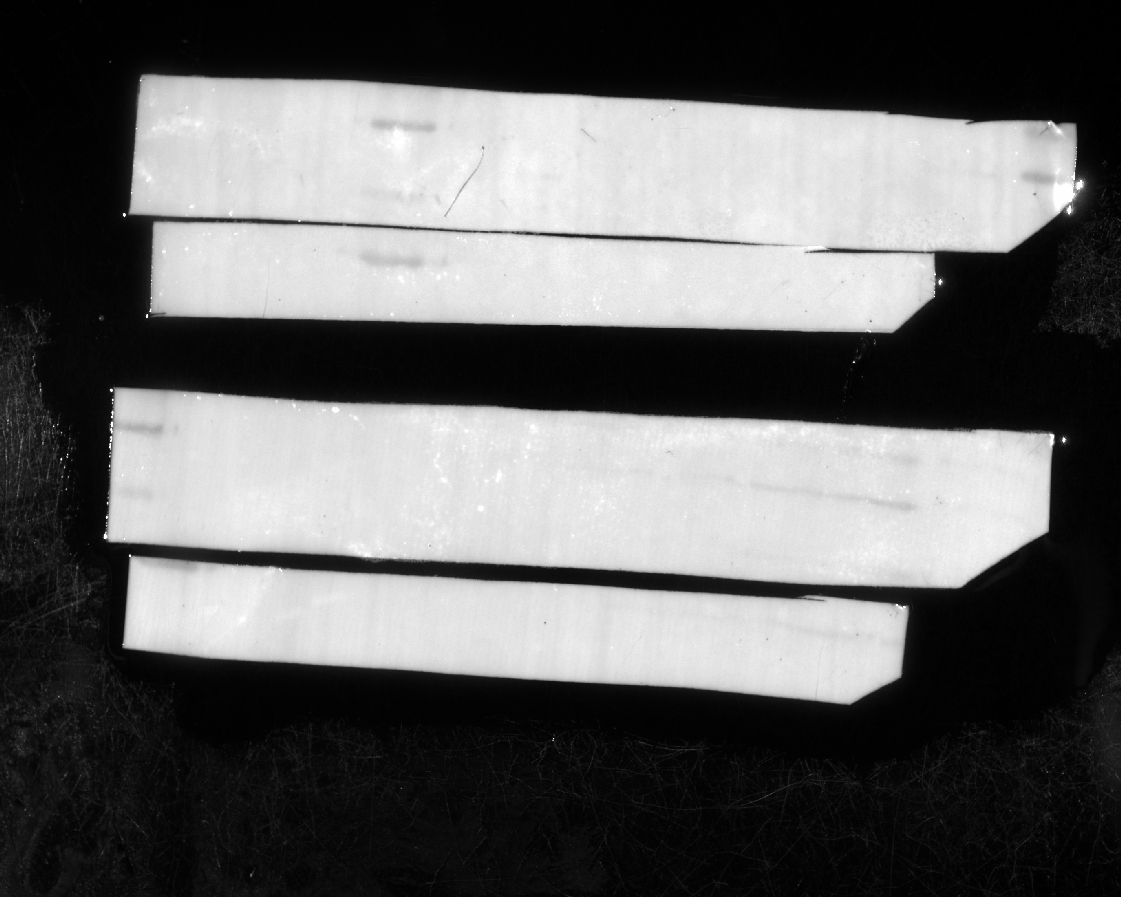


In Colorimetric mode

## References:

1. Roth K, Yang Z, Agarwal M, Liu W, Peng Z, Long Z, et al. Exposure to a mixture of legacy, alternative, and replacement per- and polyfluoroalkyl substances (PFAS) results in sex-dependent modulation of cholesterol metabolism and liver injury. Environment International. 2021;157:106843.

2. Schoeler M, Caesar R. Dietary lipids, gut microbiota and lipid metabolism. Rev Endocr Metab Disord. 2019;20:461–72.

3. Bárta F, Dedíková A, Bebová M, Dušková Š, Mráz J, Schmeiser HH, et al. Co-Exposure to Aristolochic Acids I and II Increases DNA Adduct Formation Responsible for Aristolochic Acid I-Mediated Carcinogenicity in Rats. IJMS. 2021;22:10479.

4. Suarez G, Romero-Gallo J, Piazuelo MB, Wang G, Maier RJ, Forsberg LS, et al. Modification of *Helicobacter pylori* Peptidoglycan Enhances NOD1 Activation and Promotes Cancer of the Stomach. Cancer Res. 2015;75:1749–59.

5. Kaur G, Jabbar Z, Athar M, Alam MS. Punica granatum (pomegranate) flower extract possesses potent antioxidant activity and abrogates Fe-NTA induced hepatotoxicity in mice. Food and Chemical Toxicology. 2006;44:984–93.

6. Maeda S, Shibata W, Hikiba Y, Yanai A, Sakamoto K, Nakagawa H, et al. S2052 c-Jun Nh2-Terminal Kinase 1 Is a Critical Regulator for the Development of Chemical-Induced Gastric Cancer in Mice. Gastroenterology. 2008;134:A-305.

7. Li N, Liu S, Zhang Y, Yu L, Hu Y, Wu T, et al. Transcriptional Activation of Matricellular Protein Spondin2 (SPON2) by BRG1 in Vascular Endothelial Cells Promotes Macrophage Chemotaxis. Front Cell Dev Biol. 2020;8:794.

8. Asrani SK, Devarbhavi H, Eaton J, Kamath PS. Burden of liver diseases in the world. Journal of Hepatology. 2019;70:151–71.

9. Zhang L, Yao X, Ma M, Ding Y, Zhang H, He X, et al. Protective Effect of l -Theanine against DSS-Induced Colitis by Regulating the Lipid Metabolism and Reducing Inflammation via the NF-κB Signaling Pathway. J Agric Food Chem. 2021;69:14192–203.

10. Akar F, Sumlu E, Alçığır ME, Bostancı A, Sadi G. Potential mechanistic pathways underlying intestinal and hepatic effects of kefir in high-fructose-fed rats. Food Research International. 2021;143:110287.

11. Martínez-López M, Iborra S, Conde-Garrosa R, Mastrangelo A, Danne C, Mann ER, et al. Microbiota Sensing by Mincle-Syk Axis in Dendritic Cells Regulates Interleukin-17 and -22 Production and Promotes Intestinal Barrier Integrity. Immunity. 2019;50:446-461.e9.

12. Nguyen PM, Putoczki TL. Could the inhibition of IL-17 or IL-18 be a potential therapeutic opportunity for gastric cancer? Cytokine. 2019;118:8–18.

13. Moreno-Fernandez ME, Giles DA, Oates JR, Chan CC, Damen MSMA, Doll JR, et al. PKM2-dependent metabolic skewing of hepatic Th17 cells regulates pathogenesis of non-alcoholic fatty liver disease. Cell Metabolism. 2021;33:1187-1204.e9.

14. Zhang H, Dai Y, Liu Y, Wu T, Li J, Wang X, et al. Helicobacter pylori Colonization Protects Against Chronic Experimental Colitis by Regulating Th17/Treg Balance. Inflammatory Bowel Diseases. 2018;24:1481–92.

15. Llovet JM, Kelley RK, Villanueva A, Singal AG, Pikarsky E, Roayaie S, et al. Hepatocellular carcinoma. Nat Rev Dis Primers. 2021;7:6.

16. Wang D, Sun Y, Li W, Ye F, Zhang Y, Guo Y, et al. Antiproliferative effects of the CDK6 inhibitor PD0332991 and its effect on signaling networks in gastric cancer cells. Int J Mol Med [Internet]. 2018 [cited 2022 May 13]; Available from: http://www.spandidos-publications.com/10.3892/ijmm.2018.3460

17. Gniuli D, Calcagno A, Dalla Libera L, Calvani R, Leccesi L, Caristo ME, et al. High-fat feeding stimulates endocrine, glucose-dependent insulinotropic polypeptide (GIP)-expressing cell hyperplasia in the duodenum of Wistar rats. Diabetologia. 2010;53:2233–40.

18. Jung U, Choi M-S. Obesity and Its Metabolic Complications: The Role of Adipokines and the Relationship between Obesity, Inflammation, Insulin Resistance, Dyslipidemia and Nonalcoholic Fatty Liver Disease. IJMS. 2014;15:6184–223.

19. Hu L, Shan Z, Wang F, Gao X, Tong Y. Vascular endothelial growth factor B exerts lipid-lowering effect by activating AMPK via VEGFR1. Life Sciences. 2021;276:119401.

20. Tarnawski AS, Ahluwalia A. The Critical Role of Growth Factors in Gastric Ulcer Healing: The Cellular and Molecular Mechanisms and Potential Clinical Implications. Cells. 2021;10:1964.

21. Xiang T, Yuan C, Guo X, Wang H, Cai Q, Xiang Y, et al. The novel ZEB1-upregulated protein PRTG induced by Helicobacter pylori infection promotes gastric carcinogenesis through the cGMP/PKG signaling pathway. Cell Death Dis. 2021;12:150.

22. Xia H, Dai X, Yu H, Zhou S, Fan Z, Wei G, et al. EGFR-PI3K-PDK1 pathway regulates YAP signaling in hepatocellular carcinoma: the mechanism and its implications in targeted therapy. Cell Death Dis. 2018;9:269.

23. Wang L, Yuan H, Li Y, Han Y. The role of HER3 in gastric cancer. Biomedicine & Pharmacotherapy. 2014;68:809–12.

24. Liang JQ, Teoh N, Xu L, Pok S, Li X, Chu ESH, et al. Dietary cholesterol promotes steatohepatitis related hepatocellular carcinoma through dysregulated metabolism and calcium signaling. Nat Commun. 2018;9:4490.

25. Gao N, Yang F, Chen S, Wan H, Zhao X, Dong H. The role of TRPV1 ion channels in the suppression of gastric cancer development. J Exp Clin Cancer Res. 2020;39:206.

26. Sánchez A. Growth factor- and cytokine-driven pathways governing liver stemness and differentiation. WJG. 2010;16:5148.

27. Gambardella V, Castillo J, Tarazona N, Gimeno-Valiente F, Martínez-Ciarpaglini C, Cabeza-Segura M, et al. The role of tumor-associated macrophages in gastric cancer development and their potential as a therapeutic target. Cancer Treatment Reviews. 2020;86:102015.

28. Powell EE, Wong VW-S, Rinella M. Non-alcoholic fatty liver disease. The Lancet. 2021;397:2212–24.
